# Synthesis and Biological Evaluation of Azamacrolide Comprising the Triazole Moiety as Quorum Sensing Inhibitors

**DOI:** 10.3390/molecules23051086

**Published:** 2018-05-04

**Authors:** Bin Zhang, Bingyi Guo, Yunlong Bai, Huizhe Lu, Yanhong Dong

**Affiliations:** Department of Applied Chemistry, College of Science, China Agricultural University, No.2 Yuanmingyuan West Road, Beijing 100193, China; zb309258650@163.com (B.Z.); bingyiguo618@163.com (B.G.); byl142@163.com (Y.B.); luhz@cau.edu.cn (H.L.)

**Keywords:** azamacrolides, quorum sensing inhibitors, molecular docking

## Abstract

Novel azamacrolides comprising the triazole moiety were synthesized and evaluated for their quorum sensing inhibitor activities on the *Agrobacterium tumefaciens*. It was found that the inhibition rate of compound **Z12**-**3** at 200 mg/L (0.45 mM) can reach 67%. The potential binding modes between these molecules and the TraR QS receptor was performed by molecular docking. The results showed that the two nitrogen atoms in the triazole ring of **Z12**-**3** formed hydrogen bonds with GLN-2, and the carbonyl group (C=O) in the amide formed hydrogen bonds with water. It was worth noting that the carbonyl group on the macrolides formed hydrogen bonds with the G-106 base in the DNA. These azamacrolides may block quorum sensing expression through key amino acid residues or DNA bases in the TraR QS receptor by hydrogen-bonded.

## 1. Introduction

The misuse and overuse of antibiotics in traditional therapeutics to treat bacterial infections have given rise to multi-drug resistant pathogens, which pose threats to human health and environmental safety [1].

Quorum sensing (QS) is a process that bacteria regulate biofilm formation and various virulence factors by altering gene expression through the sense of signal concentration [2,3]. Biofilm provides a multi-level protection to microbes against antibiotics [4]. Meanwhile, it also increases microbial resistance by blocking the penetration of antibiotics and reducing the direct contact between antibiotics and cells [5]. Microbial resistance to antibiotics has become a serious problem due to the abuse of antibiotics. The block of QS signaling system may attenuate bacterial pathogenicity, reduce antibiotic use and slow down the emergence of microbial resistance [5]. Therefore, the development of novel QS inhibitors is highly valuable.

The macrolides are classified into groups based on the number of atoms in the macrocyclic rings: 12, 14, 16, or larger, from a chemical viewpoint [6]. From the 1950s, macrolide antibiotics were widely used in both human and veterinary medicine to treat gram-positive and gram-negative bacteria. Macrolides also had the potential for inhibition of QS. For example, azithromycin (AZM), a 15-membered macrolide, is a semi-synthetic azamacrolide drug for the treatment of chronic respiratory infections [7]. AZM is normally not included in the antipseudomonal therapeutic arsenal because of the absence of bactericidal or bacteriostatic activity [8]. However, AZM can inhibit the *Pseudomonas aeruginosa* synthesis of autoinducers, leading to a reduction of virulence factor production [9,10]. In vitro time-kill and checkerboard studies suggested that AZM may enhance killing in combination with the polymyxins via QS [9,11].

Triazole moiety abundantly exists in drugs including β-lactam antibiotic (that is, tazobactam) and the cephalosporine (that is, cefatrizine) [12]. Many compounds containing the triazole moiety have been shown to bind diverse biological targets via hydrogen bonding and dipole interactions [12,13,14]. It has been reported that triazole derivatives showed good anti-QS activity [15,16]. Triazole-containing analogs of natural *N*-acyl l-homoserine lactone were capable of strongly modulating the activity of LasR and AbaR [15]. Gu and co-workers demonstrated that 1,4-disubstituted 1,2,3-triazoles containing isoxazole and thymidine structures can serve as potential lead structures for the development of novel QS inhibitors [16].

Molecular hybridization, the combination of different pharmacophores of bioactive compounds to obtain new molecules with potent activity, is an effective strategy in the design and development of new drugs [17,18].

Bearing this in mind, we synthesized a class of novel azamacrolides bearing the triazole moiety and evaluated their QS inhibitory activities on *Agrobacterium tumefaciens* NT1 (pZLR4) quorum. The X-ray crystal structure of TraR-OOHL (pdb 1L3L, resolution: 1.66 Å) from the *Agrobacterium tumefacien* obtained in 2002 allowed us to attempt to explore the mode of interaction between azamacrolides and the receptor in order to clarify the relationship between structure and azamacrolides activity in the receptor using the molecular docking approach [19].

## 2. Results and Discussion

### 2.1. Chemistry

**Scheme 1:**
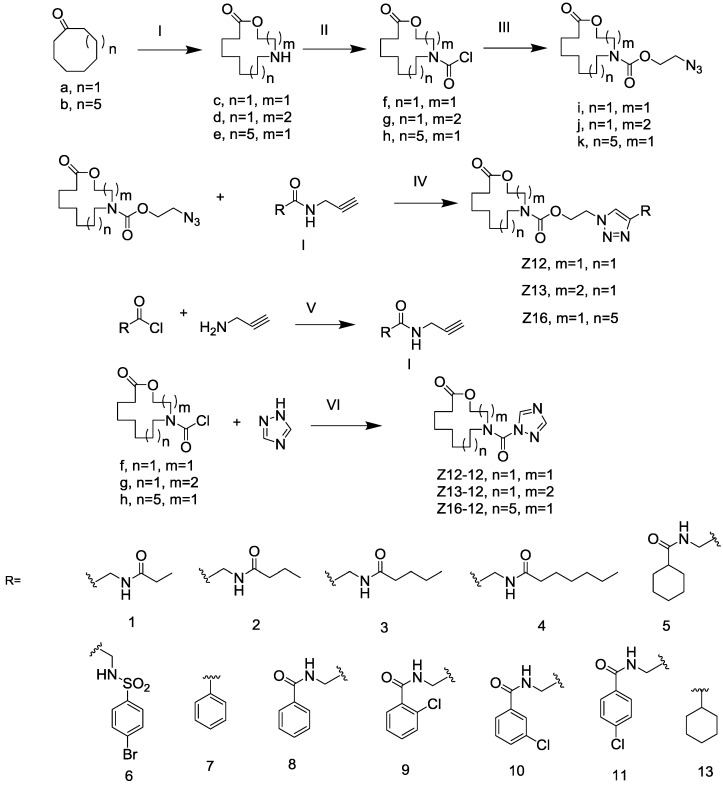
The synthesis of novel triazole containing macrolide compounds. Reagents and condition: (I) (**i**) 2-azidoethanol (**Z12** and **Z16**) or 3-azidopropanol (**Z13**), BF_3_·OEt_2_, reflux, 12 h, (**ii**) NaHCO_3_; 85–91%; (II) triphosgene, triethylamine, 0 °C, 18 h, 69–82%; (III) 2-Azidoethanol, DMAP, trimethylamine, r.t., 75–90%; (IV) CuSO_4_, Vitamin C sodium, r.t., 70–95%; (V) Et_3_N, 0 °C, 1 h, 89–92%; (VI) Et_3_N, r.t., 6 h, 85–89%.

The synthesis of the intermediates and target molecules are shown in Scheme 1. Azamacrolides **c** was prepared from the reaction of **a** and 2-azidoethanol, **d** was prepared from the reaction of **a** and 3-azidopropanol, and **e** was prepared from the reaction of **b** and 2-azidoethanol [20]. Triphosgene was used to obtain carbamic chlorides. Carbamates **i**, **j**, and **k** were prepared by the reaction of 2-azidoethanol with carbamic chloride **f**, **g**, and **h**. *N*-Propargyl amides **l** were synthesized from commercially available acid chloride and prop-2-yn-1-amine. Finally, 1,2,3-triazoles were obtained through a facile copper-catalyzed azide-alkyne click chemistry. The 1,2,4-triazoles derivatives were prepared from the reaction of 1-hydro-1,2,4-triazole with compounds **f**, **g**, and **h**.

**Scheme 2:**
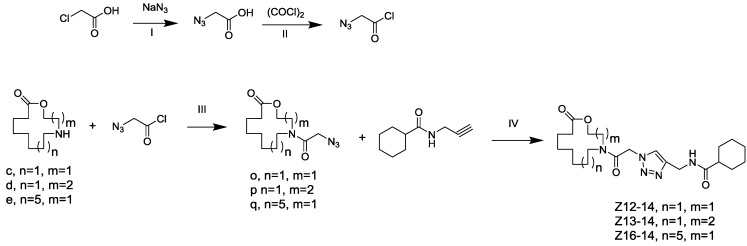
The synthesis of novel triazoles containing macrolide compounds. Reagents and condition: (I) NaN_3_, H_2_O, 36 h, 95%; (II) Oxalyl chloride, 0 °C, 3 h; (III) DMAP, trimethylamine, r.t., 85–92%; (IV) CuSO_4_, Vitamin C sodium, r.t., 80–95%.

The synthesis of the intermediates and target molecules are shown in Scheme 2. 2-azidoacetic acid was prepared from the reaction of 2-chloroacetic acid and NaN_3_. 2-azidoacetyl chloride was prepared from the reaction of 2-azidoacetic acid and Oxalyl chloride. Acylamide **o**, **p**, and **q** were prepared by the reaction of **c**, **d**, **e**, and 2-azidoacetyl chloride. Finally, 1,2,3-triazoles were obtained through a facile copper-catalyzed azide-alkyne click chemistry.

### 2.2. QS Activity.

#### 2.2.1. QS Inhibitory Activity

**Figure 1:**
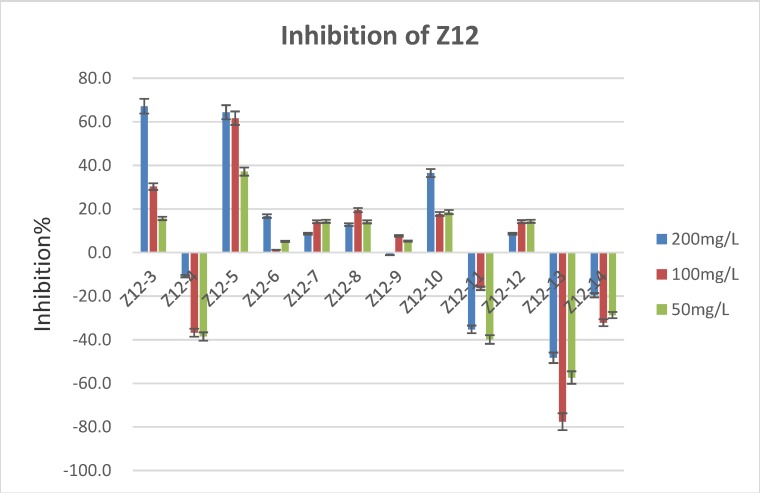
The QS inhibitory activity of the **Z12** series compounds.

**Figure 2:**
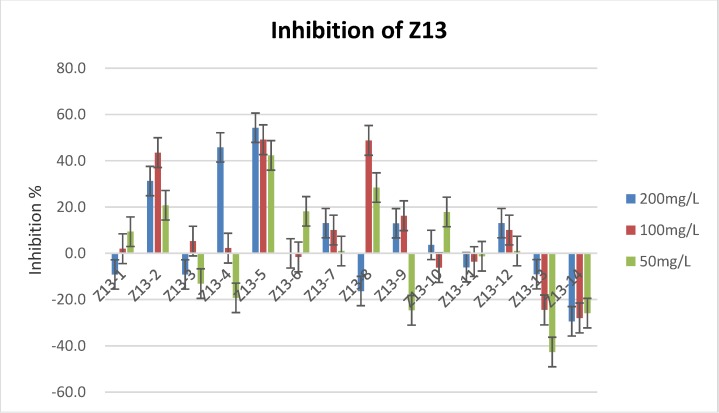
The QS inhibitory activity of the **Z13** series compounds.

**Figure 3:**
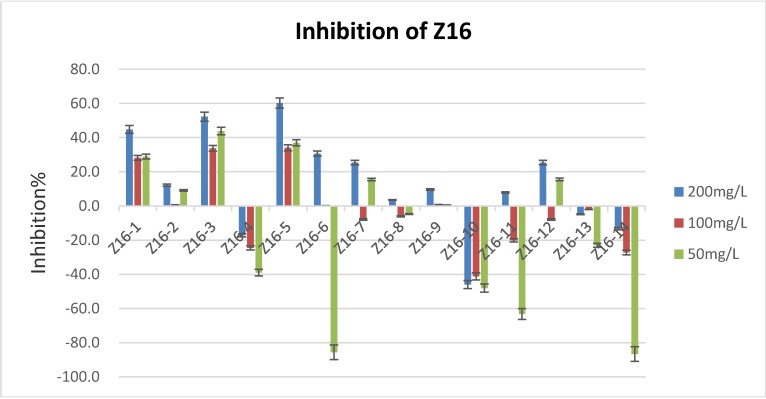
The QS inhibitory activity of the **Z16** series compounds.

The dose-response assays of compounds were evaluated in the *Agrobacterium* to inhibit the QS and the results were shown in Figure 1, Figure 2 and Figure 3 (the raw data are available in the supporting information Tables S1–S3). AMZ was a positive control (the raw data are available in the supporting information Table S1). The compounds containing a benzene ring or 1,2,4-triazole showed low inhibitory activity. The compounds **Z12**-**(6**–**12)**, **Z13**-**(6**–**12)**, **Z16**-**(6**–**12)** inhibited QS by less 36% at a concentration of 200 mg/L. The compounds containing an alkyl group or cyclohexane showed high inhibitory activity. The inhibitory activities of **Z12**-**(1**, **3**, **5**), **Z13**-**(2**, **4**, **5), Z16**-**(1**, **3**, **5)** inhibited QS by more than 54% at a concentration of 200 mg/L. The compounds **Z12**-**5**, **Z13**-**5** and **Z16**-**5** showing high inhibitory activity, and all of them contained cyclohexane. Interestingly, **Z16**-**6** and **Z16**-**11** inhibited QS by −85% and −63% at 50 mg/L and there may have been a promotion of QS activity. AMZ was a positive control and AMZ inhibited QS by 78.59% at 12.5 mg/L (16.7 nM). The Z12-3 at 200 mg/L (0.45 mM) can reach 67%. The activity of AMZ was more than 20 times higher than that of Z12-3.

#### 2.2.2. Dose–Response Bioassay

The compounds were dissolved in DMSO and diluted at a final concentration of 12.5 mg/L in the AB minimal medium [21,22]. The results of the bacterial incubated with the compounds which showed QS Inhibitory activity was shown in Table 1. The OD600 value of the bacteria with compounds added did not decline, which demonstrates that the compounds have no bactericidal activity. We can infer that the β-galactosidase (U) was reduced by inhibited QS.

#### 2.2.3. Non-Competitive Inhibition Assay

**Figure 4:**
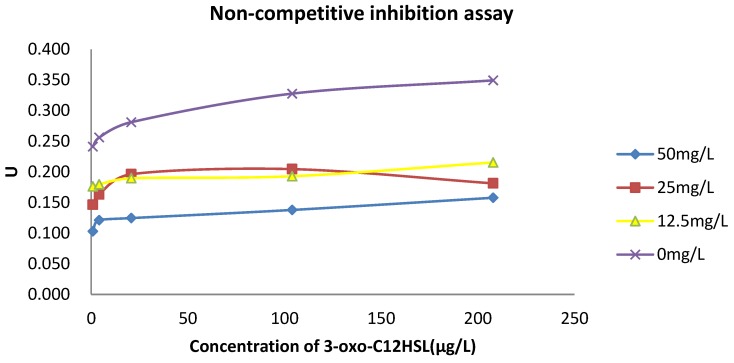
The noncompetition assay between **Z12**-**3** and the signal molecule.

The experiment results showed that there was a non-competitive inhibition between **Z12**-**3** and the signal molecule, as shown in Figure 4. When compound **Z12**-**3** was added, the activity of β-galactosidase (U) had no significant difference at the gradient concentrations of the signal molecules. This means that when **Z12**-**3** was added, it was not possible to relieve the inhibitory effect on QS by increasing the exogenous signal molecules. We can infer that there was a noncompetitive relationship between compound **Z12**-**3** and the signal molecule.

#### 2.2.4. QS Agonist

The results of the synergy assay between **Z16**-**6** and the signal molecule were shown in Table 2. When compound **Z16**-**6** at 50 mg/L was added to the signal, the activity of β-galactosidase (U) increased by 67%. However, the activity of β-galactosidase (U) was very low in the absence of the signal. So we inferred that **Z16**-**6** had no QS agonist activity and it just may promote β-galactosidase activity.

### 2.3. Docking

**Figure 5:**
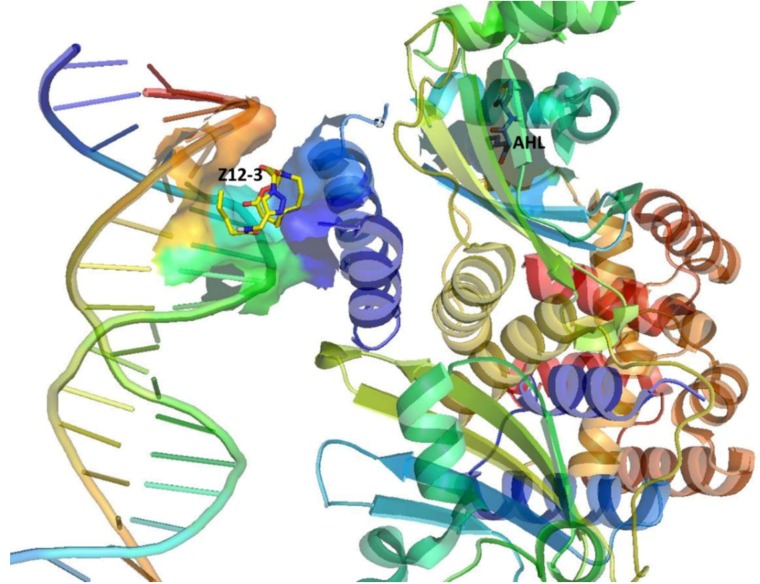
The active site pocket.

Because **Z12**-**3** and the signal molecules were in a noncompetitive relationship, we attempted the different active pocket and found one (Figure 5) that had docking scores and biological activity that matched for each other. To obtain the structural insight into the plausible interaction mode of the inhibitors interacting with TraR-OOHL (pdb 1L3L), the docking studies were performed over the respective systems. Considering the QS inhibitory activities, **Z12**-**3**, **Z16**-**9**, **Z12**-**6**, **Z13**-**6**, and **Z16**-**6** were chosen to conduct the docking calculation. For compounds **Z12**-**3** and **Z16**-**9**, the molecular docking scores were 7.54 and 3.63, and their inhibitory rates were 67% and 9%. The structure-activity relationship showed that compound **Z12**-**3** had a side chain of five carbon atom paraffins and compound **Z16**-**9** had a side chain of chlorobenzene.

**Figure 6:**
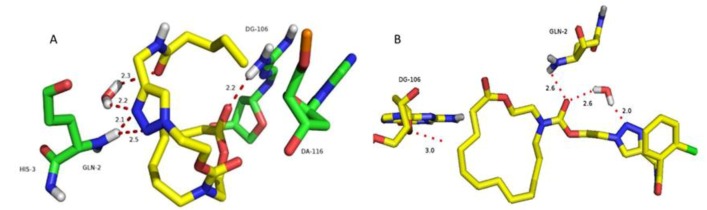
The molecular model of TraR-OOHL with **Z12**-**3** (**A**) and **Z16**-**9** (**B**), bound to the active site.

The compound **Z12**-**3** was shown as yellow stick models (Figure 6) and the key amino acid residues were shown as green stick models. The red dotted lines represented the hydrogen bonding interactions between the compounds and the amino acid residues or DNA bases. The docking positions for **Z12**-**3** indicated the presence of hydrogen bonds between the triazole ring and GLN-2, and the bond lengths were 2.1 Å and 2.5 Å. The triazole ring and the carbonyl group (C=O) in the amide (NH) formed hydrogen bonds with water. It was worth noting that the carbonyl group (C=O) on the azamacrolides formed hydrogen bonds with the DG-106 base in the DNA and the bond length was 2.2 Å. Thus, it was inferred that **Z12**-**3** may be able to interfere with the transcription of the QS gene. However, **Z16**-**9** only formed hydrogen bonds with GLN-2 and the bond length was 2.6 Å, as shown in Figure 6. So **Z16**-**9** had low activity.

**Figure 7:**
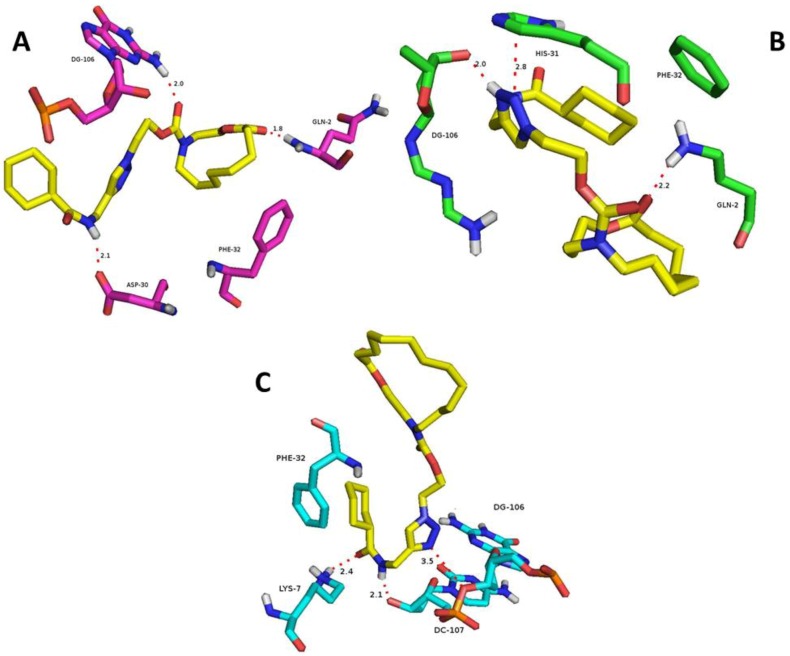
The molecular model of TraR-OOHL with **Z12**-**5** (**A**), **Z13**-**5** (**B**), and **Z16**-**5** (**C**), bound to the active site.

The compounds **Z12**-**5**, **Z13**-**5**, and **Z16**-**5** showed high inhibitory activity (64%, 54%, and 60% at 200 mg/L) and all of them containing cyclohexane. We studied the molecular docking and the scores were 7.73, 7.04, and 7.92. as shown in Figure 7. The docking positions for **Z12**-**5**, **Z13**-**5**, and **Z16**-**5** indicated the presence of hydrogen bonds between the compounds and DG-106 in the DNA, and the cyclohexane had a hydrophobic interaction with the amino acid PHE-32.

The compounds bound to the native QS ligand active site. In order to verify the active pocket available, we synthesized six compounds which did not have amides near the triazole ring or carbamates near azamacrolide. The structure-activity relationship showed that **Z12**-**13**, **Z13**-**13**, and **Z16**-**13** shared the same structure with **Z12**-**5**, **Z13**-**5**, and **Z16**-**5**, except for the amide near the triazole ring. The compounds **Z12**-**5**, **Z13**-**5**, and **Z16**-**5** showed high inhibitory activity (64%, 54%, and 60% at 200 mg/L), while **Z12**-**13**, **Z13**-**13**, and **Z16**-**13** had almost no inhibitory activity (−48.3%, −9.1%, and −4.9% at 200 mg/L). So, we inferred that the amide near the triazole ring was very important. The same applied to **Z12**-**14**, **Z13**-**14**, and **Z16**-**14**, all of which had no inhibitory activity (−19.6%, −29.4%, and −13.3% at 200 mg/L). The carbamate near the azamacrolide was irreplaceable. Additionally, the molecular docking scores varied greatly. The scores of **Z12**-**5**, **Z13**-**5**, and **Z16**-**5** were 8.13, 6.85, and 6.12. The scores of **Z12**-**13**, **Z13**-**13**, and **Z16**-**13** were 3.34, 2.86, and 2.46. The scores of **Z12**-**14**, **Z13**-**14**, and **Z16**-**14** were 2.78, 3.00, and 3.38.

## 3. Experimental

### 3.1. QS Inhibitory Activity

The *Agrobacterium* was cultured in AB minimal medium, when it grew to OD600 > 0.6. Taking 0.3 mL of bacterial suspension into a 1.5 mL microcentrifuge tube, both the test compound (soluble in DMSO) and AHL were added in, cultured at 28 °C for 3 h. The β-galactosidase activity was measured as described previously [21,22,23].

### 3.2. The Bactericidal Activities of the Compounds

The *Agrobacterium* was cultured in an AB minimal medium with the compounds and showed QS Inhibitory activity. The compounds were dissolved in DMSO and diluted at a final concentration of 12.5 mg/L in AB minimal medium. The bacterial solution was incubated with the compound for 18 h and the OD600 value was measured.

### 3.3. Noncompetition Assay

Compound **Z12**-**3** showed comparable QS inhibition and in order to verify the mechanism of action of the compounds, competition assays were tested between **Z12**-**3** and the signal molecule. Different concentrations of compound **Z12**-**3** (50, 25, and 12.5 mg/L) and the signal molecule (0.83, 4.15, 20.75, 104, and 208 µg/L) were added into bacterial solutions (OD600 > 0.6). After 1.5 h, they were tested for β-galactosidase activity. If the activity of galactosidase (U) had no significant difference at the gradient concentrations of signal molecules, it indicated the compound and the signal molecule had no competitive relationship.

### 3.4. QS Agonist

Compound **Z16**-**6** showed QS agonist activity and in order to verify the mechanism of action of **Z16**-**6**, synergy assays were tested between **Z16**-**6** and the signal molecule. Different concentrations of compound **Z16**-**6** (100 and 50 mg/L) and the signal molecule (104 µg/L) were added into bacterial solutions (OD600 > 0.6); the activity of β-galactosidase was tested after 3 h.

### 3.5. Computational Chemistry—Docking

The receptor was derived from the crystal structure of TraR-OOHL (pdb 1L3L). The Surflex-Dock program in the SYBYL 7.3 software package was used to add polar hydrogens and to save the protein in the appropriate file formate for docking with the compounds [24,25]. From the noncompetition assay data, we can infer that there was a noncompetitive relationship between compound **Z12**-**3** and the signal molecule. Furthermore, we can infer that there are different binding sites between AHL and the compounds with TraR-OOHL (pdb 1L3L). In this study, the multi-channel surface mode of the protocol was applied to define the active site. All other parameters were set to their default values. We tried different active pockets and found one (Figure 5) with docking scores and biological activity that matched for each other.

## 4. Conclusions

In conclusion, we synthesized azamacrolide comprising the triazole moiety and examined it for the ability to inhibit QS inhibitor activities on the *Agrobacterium tumefaciens*. Docking studies were performed. We attempted different active pockets and found that the docking scores and biological activity matched for each other. The triazole moiety should form hydrogen bonds with the receptor. The most active derivatives included the alkyl substituted **Z12**-**3** (67% at 200 mg/L) and included the cyclohexane substituted **Z12**-**5** (64% at 200 mg/L), **Z13**-**5** (54% at 200 mg/L), and **Z16**-**5** (60% at 200 mg/L). We can infer that there was a noncompetitive relationship between **Z12**-**3** and the signal molecules. We inferred that **Z16**-**6** had no QS agonist activity and it may just promote β-galactosidase activity. We inferred that the amide near the triazole ring was very important and that the carbamate near the azamacrolide was irreplaceable.

## Figures and Tables

**Table 1:** The results of the bactericidal activity.

Comp.	OD600	Inhibition (%)	Comp.	OD600	Inhibition (%)
Blank	0.664	---	DMSO	0.683	−2.2
Z12-2	0.73	−9.9	Z16-3	0.838	−25.5
Z12-9	0.826	−22.2	Z16-9	0.845	−21.6
Z13-1	0.811	−17.8	Z16-10	0.868	−24.1
Z13-3	0.85	−22.9	Z16-11	0.815	−17.4

**Table 2:** The synergy assay between **Z16**-**6** and the signal molecule.

Group	U	Inhibition (%)
signal	0.47	——
signal + 100 mg/L	0.46	1.05
signal + 50 mg/L	0.78	−66.67
100 mg/L	0.04	——
50 mg/L	0.03	——

## Supplementary Materials

Supplementary materials are available online.
