# Supplementary material for: Synthesis and Biological Evaluation of Azamacrolide Comprising the Triazole Moiety as Quorum Sensing Inhibitors
Source: Molecules. 2018 May 4;23(5):1086. doi: 10.3390/molecules23051086 (PMC6102594; doi:10.3390/molecules23051086)
Supplement: Supplementary file 1 [file molecules-23-01086-s001.pdf]

Synthesis and Biological Evaluation of Azamacrolide Comprising Triazole Moiety as Quorum Sensing Inhibitors

Bin Zhang, Bingyi Guo, Yunlong Bai, Huizhe Lu, Yanhong Dong*

College of Science, China Agricultural University, No.2 Yuanmingyuan West Road, Beijing 100193, China.

*dongyh@cau.edu.cn

Supplementary Information

1. Table of Contents **S1**

2. General Information **S2**

3. ^{1}H and ^{13}C NMR Spectra **S3**

4. Inhibition rate **S4**

5. References **S5**

2. General Information

Glassware was dried in an oven overnight before use. Thin layer chromatography was carried out on SIL G/UV254 silica-aluminum plates and plates were visualized using ultra-violet light (254 nm) and I_2. For flash column chromatography, silica gel 60, 35-70 μ was used. NMR data was collected at 300 MHz. Data was manipulated directly from the spectrometer or *via* a networked PC with appropriate software. All samples were analyzed in $CDCl_3$ unless otherwise stated. Reference values for residual solvent were taken as δ = 7.27 ($CDCl_3$) for ^{1}H-NMR; δ = 77.1 ($CDCl_3$) for ^{13}C-NMR. Multiplicities for coupled signals were designated using the following abbreviations: s = singlet, d = doublet, t = triplet, q = quartet, quin = quintet, br = broad signal, and are given in Hz. High-resolution mass spectra (ESI-HRMS) were recorded on a Bruker Daltonics Bio-TOF-Q III mass spectrometer (Bruker Co., Karlsruhe, Germany).

All reagents used were of analytical grade and the solvents were dried according to standard procedures.

3. ^{1}H and ^{13}C NMR Spectra

c. 1-oxa-4-azacyclododecan-12-one

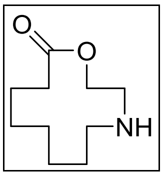

Compound **c** was synthesized in 74% yield according to the procedure reported in Ref [1]. ^{1}H-NMR (300 MHz, $CDCl_3$): δ 4.36-4.11 (m, 2H), 2.96-2.83 (m, 2H), 2.74-2.62 (m, 2H), 2.44-2.27 (m, 2H), 1.88-1.64 (m, 2H), 1.57-1.26 (m, 10H). 13C-NMR (75 MHz, $CDCl_3$): δ 173.7, 62.9, 46.5, 46.2, 34.5, 25.7, 25.2, 25.0, 23.0, 22.7. HRMS (m/z) calcd for $C_{10}H_{19}NO_2$ $[M+H]^+$ 186.1489, found 186.1485.

f. 12-oxo-1-oxa-4-azacyclododecane-4-carbonyl chloride

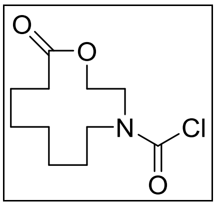

Under the ice water bath, the triphosgene (12.81 g, 43.18 mmol) was added in a dry three-necked flask, dissolved in dichloromethane (25 mL). And the compound **c** (20 g, 107.95 mmol) and Et_3N (10.92 g, 107.95 mmol) dissolved in dichloromethane (15 mL) were added dropwise. Then they reacted at room temperature for 10 h. The mixture rotary evaporated to get colorless viscous liquid, silica gel column chromatography (ethyl acetate : Petroleum ether=1:5), to get a colorless viscous liquid (15.60 g, 62.97 mmol), a yield of 58%. The compound used for the next reaction directly.

i. 2-azidoethyl 12-oxo-1-oxa-4-azacyclododecane-4-carboxylate

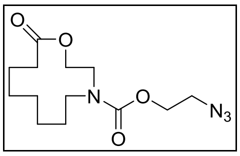

The compound **f** (15 g, 60.55 mmol) and 2-azidoethan-1-ol (5.80 g, 66.61 mmol) were added in a dry three-necked flask, dissolved in dichloromethane (25 ml), then Et_3N(6.15 g) was added in the three-necked flask, and reacted at room temperature for 18 h. The mixture rotary evaporated to get dark brown viscous liquid, silica gel column chromatography (ethyl acetate : Petroleum ether=1:5), to get a white solid(13.5 g, 45.25 mmol), a yield of 74%.

^{1}H NMR (300 MHz, $CDCl_3$) δ 4.30 (ddd, J = 14.7, 9.2, 5.5 Hz, 4H), 3.61 (dd, J = 9.3, 4.9 Hz, 2H), 3.48 (dd, J = 6.5, 3.5 Hz, 2H), 3.34 (dd, J = 13.1, 6.5 Hz, 2H), 2.47 – 2.36 (m, 2H), 1.69 (ddd, J = 27.8, 14.0, 4.5 Hz, 4H), 1.45 (dd, J = 13.4, 6.9 Hz, 6H).

Z12-1. 2-(4-(propionamidomethyl)-1H-1,2,3-triazol-1-yl)ethyl 12-oxo-1-oxa-4-azacyclododecane-4-carboxylate

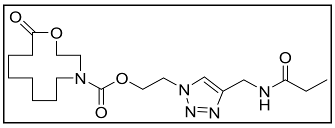

The compound **i** (0.5g, 1.68mmol) and *N*-ethynylpropionamide (0.17 g, 1.76 mmol) were added

in a three-necked flask, dissolved in methanol (10 ml), then $CuSO_4$(0.2 g, 1.25 mmol) and vitamin C sodium salt(0.25 g, 1.25 mmol) were added in the three-necked flask, and reacted at room temperature for 0.5 h. The mixture rotary evaporated to get dark green viscous liquid, silica gel column chromatography (ethyl acetate), to get a white solid(0.63 g, 1.54 mmol), a yield of 92 %. ^{1}H NMR (300 MHz, DMSO) δ 8.24 (s, 1H), 7.91 (s, 1H), 4.43-4.00 (m, 6H), 3.54-3.06 (m, 4H), 2.41-2.30 (m, 2H), 2.09 (m 2H), 1.68-1.54 (m, 2H), 1.53-1.15 (m, 10H), 0.99 (t, *J* = 7.5 Hz, 3H). ^{13}C NMR (75 MHz, DMSO) δ 172.8, 172.7, 155.4, 145.1, 123.0, 63.6, 63.3, 48.6, 47.2, 34.0, 33.4, 28.2, 24.8, 23.7, 22.5, 21.9, 9.7. HRMS (m/z) calcd for $C_{19}H_{31}N_5O_5$ $[M+H]^+$ 410.2398, found 410.2395.

Z12-2. 2-(4-(butyramidomethyl)-1H-1,2,3-triazol-1-yl)ethyl 12-oxo-1-oxa-4-azacyclododecane-4-carboxylate

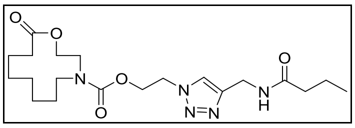

The compound **i** (0.5 g, 1.68 mmol) and *N*-(prop-2-yn-1-yl)butyramide (0.22 g, 1.76 mmol) were added in a three-necked flask, dissolved in methanol (10 ml), then $CuSO_4$ (0.2 g, 1.25 mmol) and vitamin C sodium salt(0.25 g, 1.25 mmol) were added in the three-necked flask, and reacted at room temperature for 0.5 h. The mixture rotary evaporated to get dark green viscous liquid, silica gel column chromatography (ethyl acetate), to get a white solid(0.67 g, 1.58 mmol), a yield of 94 %. ^{1}H NMR (300 MHz, $CDCl_3$) δ 7.58 (s, 1H), 6.40 (m, 1H), 4.62 (t, *J* = 5.1 Hz, 2H), 4.54-4.46 (m, 4H), 4.31 (m, 1H), 4.05 (m, 1H), 3.57 (m 1H), 3.43 (m, 1H), 3.31 (m, 1H), 3.21 (m, 1H), 2.41 (m, *J* = 6.2 Hz, 2H), 2.17 (t, *J* = 7.5 Hz, 2H), 1.71-1.54 (m, 4H), 1.50-1.30 (m, 8H), 0.92 (t, *J* = 7.4 Hz, 3H). ^{13}C NMR (75 MHz, $CDCl_3$)δ 173.6, 173.2, 155.9, 130.8, 122.9, 64.1, 63.2, 49.5, 47.8, 38.2, 34.7, 33.9, 28.8, 25.4, 25.1, 24.7, 23.9, 22.4, 19.9, 13.7. HRMS (m/z) calcd for $C_{20}H_{33}N_5O_5$ $[M+H]^+$ 424.2554, found 424.2548.

Z12-3. 2-(4-(pentanamidomethyl)-1H-1,2,3-triazol-1-yl)ethyl 12-oxo-1-oxa-4-azacyclododecane-4-carboxylate

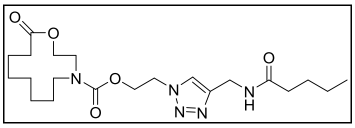

The compound **i** (0.5g, 1.68mmol) and *N*-(prop-2-yn-1-yl)pentanamide (0.25 g, 1.76 mmol) were added in a three-necked flask, dissolved in methanol (10 ml), then $CuSO_4$ (0.2 g, 1.25 mmol) and vitamin C sodium salt(0.25 g, 1.25 mmol) were added in the three-necked flask, and reacted at room temperature for 0.5 h. The mixture rotary evaporated to get dark green viscous liquid, silica gel column chromatography (ethyl acetate), to get a white solid(0.66 g, 1.51 mmol), a yield of 90 %. ^{1}H NMR (300 MHz, $CDCl_3$) δ 7.63 (s, 1H), 6.92-6.73 (d, 1H), 4.62 (t, *J* = 5.1, 2H), 4.53-4.44 (m, 4H), 4.34-4.26 (m, 1H), 4.10-4.02 (m, 1H), 3.61-3.52 (m, 1H), 3.47-3.41 (m, 1H), 3.31 (t, *J* = 6.7, 1H), 3.21 (t, *J* = 6.8, 1H), 2.45-2.35 (m, 2H), 2.20 (m, *J* = 7.6, 2H), 1.78-1.65 (m, 2H), 1.67-1.53 (m, 4H), 1.52-1.25 (m, 8H), 0.89 (t, *J* = 7.3, 3H). ^{13}C NMR (75 MHz, $CDCl_3$) δ 173.6, 173.4, 155.9, 130.9, 122.8, 64.6, 63.2, 49.5, 47.8, 36.1, 34.7, 33.9, 27.6, 25.7, 25.4, 25.1, 24.2, 23.9, 22.9, 22.3, 13.7. HRMS (m/z) calcd for $C_{21}H_{35}N_5O_5$ [M+H]+ 438.2711, found 438.2704.

Z12-4. 2-(4-(heptanamidomethyl)-1H-1,2,3-triazol-1-yl)ethyl 12-oxo-1-oxa-4-azacyclododecane-4-carboxylate

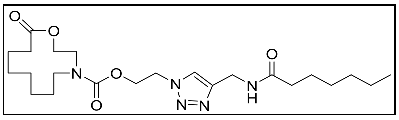

The compound **i** (0.5 g, 1.68 mmol) and *N*-(prop-2-yn-1-yl)heptanamide (0.29 g, 1.76 mmol) were added in a three-necked flask, dissolved in methanol (10ml), then $CuSO_4$ (0.2 g, 1.25 mmol) and vitamin C sodium salt(0.25 g, 1.25 mmol) were added in the three-necked flask, and reacted at room temperature for 0.5 h. The mixture rotary evaporated to get dark green viscous liquid, silica gel column chromatography (ethyl acetate), to get a white solid(0.71 g, 1.51 mmol), a yield of 91 %.

^{1}H NMR (300 MHz, $CDCl_3$) δ 7.58 (s, 1H), 6.33 (m, 1H), 4.62 (t, *J* = 5.0 Hz, 2H), 4.54-4.45 (m, 4H), 4.34-4.27 (m, 1H), 4.07-4.01 (m, 1H), 3.60-3.52 (s, 1H), 3.47-3.40 (m, 1H), 3.35-3.27 (m, 1H), 3.25-3.16 (m, 1H), 2.45-2.37 (m, 2H), 2.19 (t, *J* = 7.7 Hz, 2H), 1.76-1.57 (m, 6H), 1.41-1.28 (m, , 12H), 0.87 (t, *J* = 5.7 Hz, 3H). ^{13}C NMR (75 MHz, $CDCl_3$) δ 173.7, 155.6, 146.8, 122.7, 122.4, 64.3, 63.3, 49.6, 49.0, 48.3, 48.0, 36.6, 34.9, 34.0, 31.6, 29.7, 29.4, 29.0, 25.6, 25.2, 23.1, 22.5, 14.0.

HRMS (m/z) calcd for $C_{23}H_{39}N_5O_5$ [M+H]+ 466.3024, found 466.3018.

Z12-5. 2-(4-(cyclohexanecarboxamidomethyl)-1H-1,2,3-triazol-1-yl)ethyl 12-oxo-1-oxa-4-azacyclododecane-4-carboxylate

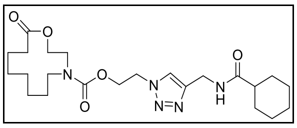

The compound **i** (0.5 g, 1.68 mmol) and *N*-(prop-2-yn-1-yl)cyclohexanecarboxamide (0.29 g, 1.76 mmol) were added in a three-necked flask, dissolved in methanol (10ml), then $CuSO_4$ (0.2 g, 1.25 mmol) and vitamin C sodium salt(0.25 g, 1.25 mmol) were added in the three-necked flask, and reacted at room temperature for 0.5 h. The mixture rotary evaporated to get dark green viscous liquid, silica gel column chromatography (ethyl acetate), to get a white solid(0.73 g, 1.57 mmol), a yield of 94 %.

^{1}H NMR (300 MHz, DMSO) δ 8.17 (s, 1H), 7.87 (s, 1H), 4.61 (t, *J* = 4.7 Hz, 2H), 4.4-4.221 (m, 4H), 4.22-4.02 (m, 2H), 3.52-3.29 (m, 2H), 3.26-3.06 (m, 2H), 2.43-2.29 (m, 2H), 2.17-2.03 (m, 1H), 1.75-1.42 (m, 8H), 1.41-1.08 (m, 12H). ^{13}C NMR (75 MHz, $CDCl_3$) δ 176.2, 173.7, 156.0, 145.2, 122.8, 64.2, 63.3, 49.6, 48.9, 48.3, 47.9, 45.2, 34.72, 34.0, 29.7, 29.6, 25.7, 25.2, 24.3, 24.0, 23.1, 23.0, 22.5.

HRMS (m/z) calcd for $C_{23}H_{37}N_5O_5$ [M+H]+ 464.2867, found 464.2862.

Z12-6. 2-(4-(((4-bromophenyl)sulfonamido)methyl)-1H-1,2,3-triazol-1-yl)ethyl 12-oxo-1-oxa-4-azacyclododecane-4-carboxylate

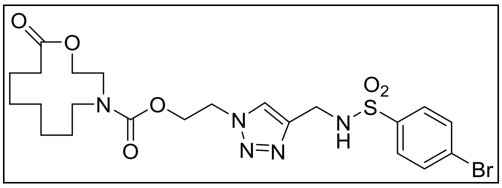

The compound **i** (0.5 g, 1.68 mmol) and *N*-(prop-2-yn-1-yl)benzamide (0.48 g, 1.76 mmol) were added in a three-necked flask, dissolved in methanol (10ml), then $CuSO_4$ (0.2 g, 1.25 mmol) and vitamin C sodium salt(0.25 g, 1.25 mmol) were added in the three-necked flask, and reacted at room temperature for 0.5 h. The mixture rotary evaporated to get dark green viscous liquid, silica gel column chromatography (ethyl acetate), to get a white solid(0.79 g, 1.38 mmol), a yield of 82 %.

^{1}H NMR (300 MHz, $CDCl_3$) δ 7.79-7.68 (m, 2H), 7.67-7.60 (m, 2H), 7.60-7.54 (m, 1H),6.40-6.08 (m,

1H), 4.66-4.55 (m, 2H), 4.46 (t, J = 5.1 Hz, 2H), 4.28-4.21 (m, 3H), 4.04-3.96 (m, 1H), 3.59-3.38 (m, 2H), 3.34-3.16 (m, 2H), 2.45-2.36 (m, 2H), 1.76-1.52 (m, 4H), 1.51-1.22 (m, 6H). ^{13}C NMR (75 MHz, $CDCl_3$) δ 173.8, 155.6, 143.8, 139.1, 132.4, 132.4, 128.7, 128.7, 127.6, 122.9, 64.2, 63.2, 49.5, 48.4, 48.0, 38.6, 34.1, 25.6, 25.2, 24.5, 23.0, 22.4. HRMS (m/z) calcd for $C_{22}H_{30}BrN_5O_6S$ [M+H]+ 570.1027, found 570.1028.

Z12-7. 2-(4-phenyl-1H-1,2,3-triazol-1-yl)ethyl 12-oxo-1-oxa-4-azacyclododecane-4-carboxylate

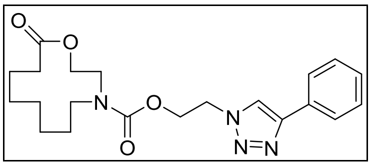

The compound **i** (0.5 g, 1.68 mmol) and ethynylbenzene (0.18 g, 1.76 mmol) were added in a three-necked flask, dissolved in methanol (10ml), then $CuSO_4$ (0.2 g, 1.25 mmol) and vitamin C sodium salt(0.25 g, 1.25 mmol) were added in the three-necked flask, and reacted at room temperature for 0.5 h. The mixture rotary evaporated to get dark green viscous liquid, silica gel column chromatography (ethyl acetate), to get a white solid(0.64 g, 1.60 mmol), a yield of 95 %.

^{1}H NMR (300 MHz, $CDCl_3$) δ 7.86-7.79 (m, 3H), 7.47-7.38 (m, 2H), 7.37-7.30 (m, 1H), 4.70 (t, J = 5.2 Hz, 2H), 4.61-4.52 (m, 2H), 4.36-4.28 (m, 1H), 4.21-414 (m, 1H), 4.17 (s, 1H), 3.60-3.40 (m, 2H), 3.36-3.17 (m, 2H), 2.43-2.32 (m, 2H), 1.74-1.53 (m, 4H), 1.48-1.24 (m, 6H). ^{13}C NMR (75 MHz, $CDCl_3$) δ 173.5, 155.9, 155.6, 147.8, 130.4, 128.8, 128.1, 125.6, 120.0, 64.1, 63.2, 49.5, 48.7, 48.2, 47.9, 33.9, 25.6, 25.2, 25.0, 24.1, 23.8, 22.8, 22.3. HRMS (m/z) calcd for $C_{23}H_{30}ClN_5O_5$ [M+H]+ 400.2118, found 400.2114.

Z12-8. 2-(4-(benzamidomethyl)-1H-1,2,3-triazol-1-yl)ethyl 12-oxo-1-oxa-4-azacyclododecane-4-carboxylate

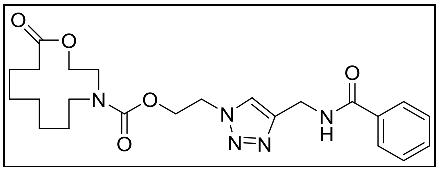

The compound **i** (0.5 g, 1.68 mmol) and *N*-(prop-2-yn-1-yl)benzamide (0.28 g, 1.76 mmol) were added in a three-necked flask, dissolved in methanol (10ml), then $CuSO_4$ (0.2 g, 1.25 mmol) and vitamin C sodium salt(0.25 g, 1.25 mmol) were added in the three-necked flask, and reacted at room temperature for 0.5 h. The mixture rotary evaporated to get dark green viscous liquid, silica gel column chromatography (ethyl acetate), to get a white solid(0.69 g, 1.51 mmol), a yield of 90 %.

^{1}H NMR (300 MHz, $CDCl_3$) δ 7.86-7.77 (m, 2H), 7.71-7.66 (m, 1H), 7.54-7.37 (m, 3H), 7.35-7.13 (d, 1H), 4.75-4.68 (m, 2H), 4.62 (t, J = 5.1 Hz, 2H), 4.59 (t, J = 5.1 Hz,2H), 4.34-4.21 (m, 1H), 4.05-3.98 (m, 1H), 3.57-3.50 (m, 1H), 3.43-3.35 (m, 1H), 3.32-3.23 (m, 1H), 3.21-3.12 (m, 1H), 2.44-2.33 (m, 2H), 1.89 (m, 1H), 1.74-1.64 (m, 2H), 1.61-1.51 (m, 1H), 1.46-1.24 (m, 8H). ^{13}C NMR (75 MHz, $CDCl_3$) δ 173.6, 167.4, 155.9, 145.1, 134.0, 131.5, 128.4, 128.4, 127.1, 127.1, 123.2, 64.2, 63.2, 49.5, 48.8, 48.2, 47.8, 35.3, 33.9, 31.8, 29.6, 25.1, 22.4. HRMS (m/z) calcd for$C_{23}H_{31}N_5O_5$ [M+H]+ 458.2398, found 458.2391.

Z12-9. 2-(4-((2-chlorobenzamido)methyl)-1H-1,2,3-triazol-1-yl)ethyl 12-oxo-1-oxa-4-azacyclododecane-4-carboxylate

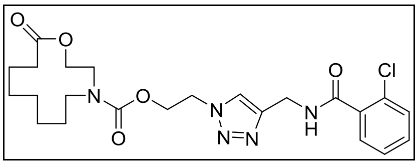

The compound **i** (0.5 g, 1.68 mmol) and 2-chloro-*N*-(prop-2-yn-1-yl)benzamide (0.34 g, 1.76 mmol) were added in a three-necked flask, dissolved in methanol (10ml), then $CuSO_4$ (0.2 g, 1.25 mmol) and vitamin C sodium salt(0.25 g, 1.25 mmol) were added in the three-necked flask, and reacted at room temperature for 0.5 h. The mixture rotary evaporated to get dark green viscous liquid, silica gel column chromatography (ethyl acetate), to get a white solid(0.73 g, 1.48 mmol), a yield of 88 %.

^{1}H NMR (300 MHz, $CDCl_3$) δ 7.72-7.59 (m, 2H), 7.43-7.29 (m, 3H), 7.15-6.90 (m, 1H), 4.79-4.70 (m, 2H), 4.63 (t, *J* = 5.1, 2H), 4.50 (t, *J* = 5.1, 2H), 4.34-4.24 (m, 1H), 4.06-3.98 (m, 1H), 3.60-3.0 (m, 1H), 3.46-3.38(m, 1H), 3.33-3.15 (m, 2H), 2.44-2.37 (m, 2H), 1.77-1.51 (m, 4H), 1.49-1.21 (m, 6H). ^{13}C NMR (75 MHz, $CDCl_3$) δ 173.7, 166.6, 156.0, 144.6, 135.0, 131.4, 130.8, 130.2, 130.0, 127.0, 122.9, 64.2, 63.3, 49.6, 47.9, 35.9, 33.9, 25.7, 25.4, 25.1, 23.9, 23.0, 22.5. HRMS (m/z) calcd for $C_{23}H_{30}ClN_5O_5$ [M+H]+ 492.2008, found 492.2005.

Z12-10. 2-(4-((3-chlorobenzamido)methyl)-1H-1,2,3-triazol-1-yl)ethyl 12-oxo-1-oxa-4-azacyclododecane-4-carboxylate

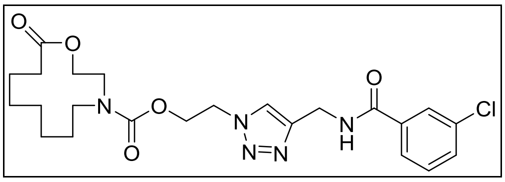

The compound **i** (0.5 g, 1.68 mmol) and 3-chloro-*N*-(prop-2-yn-1-yl)benzamide (0.34 g, 1.76 mmol) were added in a three-necked flask, dissolved in methanol (10ml), then $CuSO_4$ (0.2 g, 1.25 mmol) and vitamin C sodium salt(0.25 g, 1.25 mmol) were added in the three-necked flask, and reacted at room temperature for 0.5 h. The mixture rotary evaporated to get dark green viscous liquid, silica gel column chromatography (ethyl acetate), to get a white solid(0.72 g, 1.48 mmol), a yield of 86 %.

^{1}H NMR (300 MHz, $CDCl_3$) δ 7.87-7.79 (m, 1H), 7.75-7.64 (m, 2H), 7.51-7.33 (m, 3H), 4.77-4.68 (m, 2H), 4.67-4.60 (m, 2H), 4.56-4.46 (m, 2H), 4.33-4.24 (m, 1H), 4.03-3.94 (m, 1H), 3.59-3.51 (m, 1H), 3.44-3.36 (m, 1H), 3.34-3.25 (m, 1H), 3.23-3.13 (m, 1H), 2.46-2.34 (m, 2H), 1.78-1.51 (m, 4H), 1.49-1.23 (m, 6H). ^{13}C NMR (75 MHz, $CDCl_3$) δ 173.7, 166.6, 155.0, 145.0, 135.1, 131.2, 130.8, 130.2, 129.8, 127.0, 122.8, 63.7, 63.3, 49.5, 49.2, 48.6, 47.6, 46.8, 35.6, 33.8, 27.5, 27.2, 26.9, 26.5, 26.1, 24.9, 23.8. HRMS (m/z) calcd for $C_{23}H_{30}ClN_5O_5$ [M+H]+ 492.2008, found 492.2004.

Z12-11. 2-(4-((4-chlorobenzamido)methyl)-1H-1,2,3-triazol-1-yl)ethyl 12-oxo-1-oxa-4-azacyclododecane-4-carboxylate

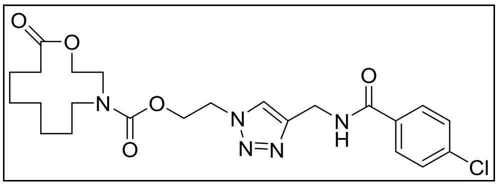

The compound **i** (0.5 g, 1.68 mmol) and 4-chloro-*N*-(prop-2-yn-1-yl)benzamide (0.34 g, 1.76 mmol) were added in a three-necked flask, dissolved in methanol (10ml), then $CuSO_4$ (0.2 g, 1.25

mmol) and vitamin C sodium salt(0.25 g, 1.25 mmol) were added in the three-necked flask, and reacted at room temperature for 0.5 h. The mixture rotary evaporated to get dark green viscous liquid, silica gel column chromatography (ethyl acetate), to get a white solid(0.75 g, 1.48 mmol), a yield of 90 %.

H NMR (300 MHz, $CDCl_3$) δ 7.85-7.75 (m, 2H), 7.74-7.54 (m, 2H), 7.42-7.33 (m, 2H), 4.77-4.58 (m, 4H), 4.56-4.45 (m, 2H), 4.33-4.22 (m, 1H), 4.05-3.96 (m, 1H), 3.58-3.49 (m, 1H), 3.44-3.35 (m, 1H), 3.33-3.12 (m, 2H), 2.45-2.32 (m, 2H), 1.7611.50 (m, 3H), 1.49-1.24 (m, 7H). 13C NMR (75 MHz, CDCl3) δ 173.5, 173.3, 166.1, 155.6, 155.1, 144.9, 144.7, 137.3, 132.0, 128.4, 128.3, 123.2, 123.0, 64.0, 63.8, 62.9, 49.3, 48.4, 47.8, 47.4, 47.3, 34.9, 33.5, 25.3, 25.0, 24.9, 24.8, 23.83, 23.8, 22.7, 22.5, 22.0. HRMS (m/z) calcd for $C_{23}H_{30}ClN_5O_5$ [M+H]+ 492.2008, found 492.2002.

Z12-12. 4-(1H-1,2,4-triazole-1-carbonyl)-1-oxa-4-azacyclododecan-12-one

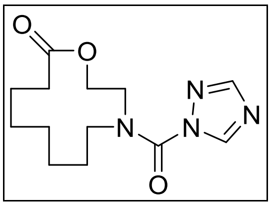

The compound **f** (0.6 g, 2.42 mmol) and 1H-1,2,4-triazole (0.18 g, 2.66 mmol) were added in a dry three-necked flask, dissolved in dichloromethane (10 ml), then Et3N(0.35 g) was added in the three-necked flask, and reacted at room temperature for 5 h. The mixture rotary evaporated to get colorless viscous liquid, silica gel column chromatography (ethyl acetate:Petroleum ether=1:1), to get a white solid(0.61 g, 2.18 mmol), a yield of 89 %.

^{1}H NMR (300 MHz, $CDCl_3$) δ 8.82 (s, 1H), 7.99 (s, 1H), 4.53-4.46 (m, 2H), 3.96-3.58 (m, 4H), 2.53-2.43 (m, 2H), 1.84-1.59 (m, 4H), 1.53-1.35 (m, 6H). ^{13}C NMR (75 MHz, $CDCl_3$) δ 173.3, 152.0, 149.7, 146.4, 61.5, 49.1, 44.1, 32.9, 28.8, 27.1, 23.9, 23.1, 22.9. HRMS (m/z) calcd for $C_{13}H_{20}N_4O_3$ [M+H]+ 281.1608, found 281.1604.

Z12-13. 2-(4-cyclohexyl-1H-1,2,3-triazol-1-yl)ethyl 12-oxo-1-oxa-4-azacyclododecane-4-carboxylate

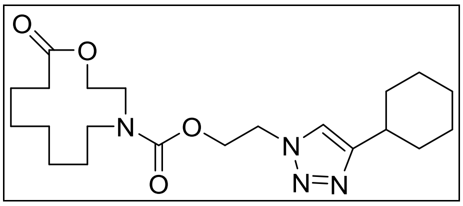

The compound **i** (0.5 g, 1.68 mmol) and ethynylcyclohexane (0.2 g, 1.84 mmol) were added in a three-necked flask, dissolved in methanol (10ml), then $CuSO_4$ (0.2 g, 1.25 mmol) and vitamin C sodium salt(0.25 g, 1.25 mmol) were added in the three-necked flask, and reacted at room temperature for 0.5 h. The mixture rotary evaporated to get dark green viscous liquid, silica gel column chromatography (ethyl acetate:Petroleum ether=1:1), to get a white solid(0.65 g, 1.68 mmol), a yield of 95 %.

^{1}H NMR (300 MHz, $CDCl_3$) δ 7.73-7.49 (m, 1H), 4.53 (m, 4H), 4.22 (m, 2H), 3.50 (m 2H), 3.25 (m 2H), 2.75 (m, 1H), 2.40 (m, 2H), 2.05 (m, 2H), 1.69 (m, 6H), 1.32 (m, 12H). ^{13}C NMR (75 MHz, $CDCl_3$) δ 173.6, 156.1, 153.9, 119.7, 64.4, 63.5, 49.3, 48.7, 48.2, 47.9, 35.3, 33.9, 33.1, 26.1, 25.7, 25.2, 25.1, 24.2, 23.9, 23.0, 22.5. HRMS (m/z) calcd for $C_{21}H_{34}N_4O_4$ [M+H]+ 407.2653, found 407.2645.

Z12-14. *N*-((1-(2-oxo-2-(12-oxo-1-oxa-4-azacyclododecan-4-yl)ethyl)-1H-1,2,3-triazol-4-yl)methyl)cyclohexanecarboxamide

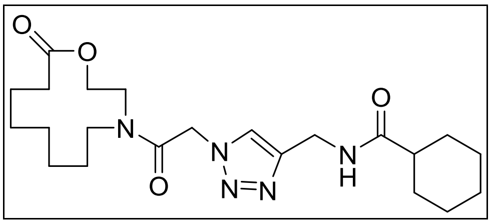

The compound **o** (0.5 g, 1.86 mmol) and ethynylcyclohexane (0.32 g, 1.96 mmol) were added in a three-necked flask, dissolved in methanol (10ml), then $CuSO_4$ (0.2 g, 1.25 mmol) and vitamin C sodium salt(0.25 g, 1.25 mmol) were added in the three-necked flask, and reacted at room temperature for 0.5 h. The mixture rotary evaporated to get dark green viscous liquid, silica gel column chromatography (ethyl acetate:Petroleum ether=1:1), to get a white solid(0.74 g, 1.71 mmol), a yield of 91 %.

^{1}H NMR (300 MHz, $CDCl_3$) δ 7.69 (s, 1H), 6.16 (s, 1H), 5.23 (s, 2H), 4.53 (d, *J* = 5.5 Hz, 2H), 4.45-4.31 (m, 2H), 3.77-3.62 (m, 2H), 3.48 (t, *J* = 7.1 Hz, 2H), 2.54-2.36 (m, 2H), 2.16-2.06 (m, 1H), 1.91-1.62 (m, 9H), 1.60 – 1.14 (m, 12H). ^{13}C NMR (75 MHz, $CDCl_3$) δ 176.2, 173.4, 165.8, 145.1, 124.0, 63.6, 51.0, 48.2, 46.8, 45.3, 35.0, 33.9, 29.6, 25.8, 25.5, 24.7, 24.6, 24.3, 23.0, 22.5.

HRMS (m/z) calcd for $C_{22}H_{35}N_5O_4$ [M+H]+ 434.2762, found 434.2754.

d. 1-oxa-5-azacyclotridecan-13-one

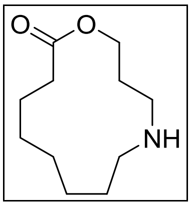

Compound **d** was synthesized in 85% yield according to the procedure reported in Ref [1].
^{1}H NMR (300 MHz, DMSO) δ 4.24-4.19 (m, 2H), 2.78-2.72 (m, 2H), 2.70-2.63 (m, 2H), 2.43-2.35 (m, 2H), 1.98 (s, 1H), 1.94-1.85 (m, 2H), 1.77-1.67 (m, 2H), 1.60-1.50 (m, 2H), 1.48-1.36 (m, 6H). ^{13}C NMR (75 MHz, $CDCl_3$) δ 173.9, 62.8, 46.1, 45.0, 33.8, 26.2, 25.3, 24.5, 23.5, 23.1, 22.7.

g. 13-oxo-1-oxa-5-azacyclotridecane-5-carbonyl chloride

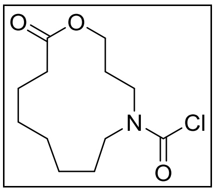

Under the ice water bath, the triphosgene (11.91 g, 40.14 mmol) was added in a dry three-necked flask, dissolved in dichloromethane (25 mL). And the compound **d** (20 g, 100.35 mmol) and Et_3N (10.16 g, 106 mmol) dissolved in dichloromethane (15 mL) were added dropwise. Then they reacted at room temperature for 10 h. The mixture rotary evaporated to get colorless viscous liquid, silica gel column chromatography (ethyl acetate : Petroleum ether=1:5), to get a colorless viscous liquid (8.31 g, 26.58 mmol), a yield of 69 %. The compound used for the next reaction directly.

j. 2-azidoethyl 13-oxo-1-oxa-5-azacyclotridecane-5-carboxylate

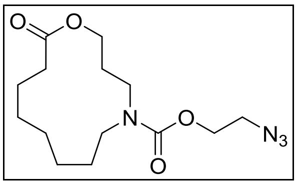

The compound **g** (10 g, 38.2 mmol) and 2-azidoethan-1-ol (3.66 g, 42.03 mmol) were added in a dry three-necked flask, dissolved in dichloromethane (25 ml), then Et_3N(4.25 g) was added in the three-necked flask, and reacted at room temperature for 18 h. The mixture rotary evaporated to get dark brown viscous liquid, silica gel column chromatography (ethyl acetate : Petroleum ether=1:5), to get a white solid(16.2 g, 61.89 mmol), a yield of 69 %.
^{1}H NMR (300 MHz, $CDCl_3$) δ 4.30-4.23 (m, 2H), 4.17 (m, 2H), 3.30 (m, 4H), 2.49-2.36 (m, 2H), 2.10-1.95 (m, 2H), 1.78-1.61 (m, 4H), 1.49-1.35 (m, 6H).

Z13-1. 2-(4-(propionamidomethyl)-1H-1,2,3-triazol-1-yl)ethyl 13-oxo-1-oxa-5-azacyclotridecane-5-carboxylate

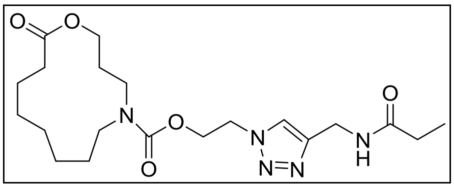

The compound **j** (0.5 g, 1.60 mmol) and *N*-(prop-2-yn-1-yl)propionamide (0.21 g, 1.68 mmol)

were added in a three-necked flask, dissolved in methanol (10ml), then $CuSO_4$ (0.2 g, 1.25 mmol) and vitamin C sodium salt(0.25 g, 1.25 mmol) were added in the three-necked flask, and reacted at room temperature for 0.5 h. The mixture rotary evaporated to get dark green viscous liquid, silica gel column chromatography (ethyl acetate), to get a white solid(0.61 g, 1.44 mmol), a yield of 90 %.

^{1}H NMR (300 MHz, $CDCl_3$) δ 7.58 (s, 1H), 6.45 (s, 1H), 4.61 (t, *J* = 5.2 Hz, 2H), 4.53-4.42 (m, 4H), 4.19-4.06 (m, 2H), 3.36-3.6 (m, 4H), 2.45- 2.36 (m, 2H), 2.29-2.18 (m, 2H), 2.01-1.87 (m, 1H), 1.83-1.65 (m, 3H), 1.61-1.30 (m, 8H), 1.15 (t, *J* = 7.6 Hz, 3H). ^{13}C NMR (75 MHz, $CDCl_3$) δ 173.9, 173.7, 155.3, 145.1, 122.8, 63.1, 61.5, 49.6, 45.4, 44.3, 34.8, 33.6, 29.4, 28.5, 27.8, 25.7, 25.3, 24.7, 24.2, 23.8, 9.7. HRMS (m/z) calcd for $C_{20}H_{33}N_5O_5$ [M+H]+ 424.2554, found 424.2548.

Z13-2. 2-(4-(butyramidomethyl)-1H-1,2,3-triazol-1-yl)ethyl 13-oxo-1-oxa-5-azacyclotridecane-5-carboxylate

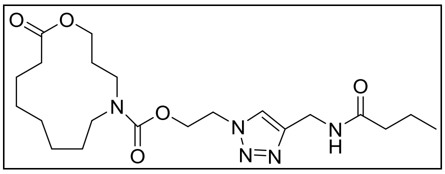

The compound **j** (0.5 g, 1.60 mmol) and *N*-(prop-2-yn-1-yl)butyramide (0.21 g, 1.68 mmol) were added in a three-necked flask, dissolved in methanol (10ml), then $CuSO_4$ (0.2 g, 1.25 mmol) and vitamin C sodium salt(0.25 g, 1.25 mmol) were added in the three-necked flask, and reacted at room temperature for 0.5 h. The mixture rotary evaporated to get dark green viscous liquid, silica gel column chromatography (ethyl acetate), to get a white solid(0.63 g, 1.44 mmol), a yield of 90 %.

^{1}H NMR (300 MHz, $CDCl_3$) δ 7.60 (s, 1H), 6.63 (s, 1H), 4.61 (t, *J* = 5.1 Hz, 2H), 4.53-4.41 (m, 4H), 4.18-4.05 (m, 2H), 3.36-3.04 (m, 4H), 2.45-2.36 (m, 2H), 2.18 (t, *J* = 7.5 Hz, 3H), 1.99-1.87 (s, 1H), 1.81-1.51 (s, 5H), 1.50-1.29 (m, 8H), 0.92 (t, *J* = 7.4 Hz, 3H). ^{13}C NMR (75 MHz, $CDCl_3$) δ 173.7, 173.2, 155.3, 145.1, 122.8, 63.1, 61.5, 49.6, 45.4, 44.3, 38.4, 34.8, 33.64, 27.8, 25.7, 25.3, 24.7, 24.2, 23.8, 19.1, 13.8. HRMS (m/z) calcd for $C_{21}H_{35}N_5O_5$ [M+H]+ 438.2711, found 438.2705.

Z13-3. 2-(4-(pentanamidomethyl)-1H-1,2,3-triazol-1-yl)ethyl 13-oxo-1-oxa-5-azacyclotridecane-5-carboxylate

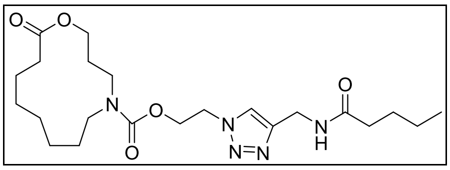

The compound **j** (0.50 g, 1.60 mmol) and *N*-(prop-2-yn-1-yl)pentanamide (0.23 g, 1.68 mmol) were added in a three-necked flask, dissolved in methanol (10ml), then $CuSO_4$ (0.2g, 1.25mmol) and vitamin C sodium salt(0.25 g, 1.25 mmol) were added in the three-necked flask, and reacted at room temperature for 0.5 h. The mixture rotary evaporated to get dark green viscous liquid, silica gel column chromatography (ethyl acetate), to get a white solid(0.62 g, 1.37 mmol), a yield of 86 %.

^{1}H NMR (300 MHz, $CDCl_3$) δ 7.55 (s, 1H), 6.17 (s, 1H), 4.60 (t, *J* = 5.2 Hz, 2H), 4.54-4.41 (m, 4H), 4.18-4.06 (m, 3H), 3.36-3.05 (m, 4H), 3.10 (s, 1H), 2.45-2.37 (m, 2H), 2.19 (t, *J* = 7.7, 2H), 1.99-1.87 (m, 1H), 1.83-1.52 (m, 8H), 1.50-1.28 (m, 10H), 0.90 (t, *J* = 7.3, 3H). ^{13}C NMR (75 MHz, $CDCl_3$) δ 173.6, 173.3, 155.2, 144.9, 122.7, 63.0, 61.3, 49.5, 45.3, 44.2, 36.0, 34.6, 33.5, 27.6, 25.6, 24.6, 23.6, 22.3, 13.6. HRMS (m/z) calcd for $C_{22}H_{37}N_5O_5$ [M+H]+ 452.2867, found 452.2860.

Z13-4. 2-(4-(heptanamidomethyl)-1H-1,2,3-triazol-1-yl)ethyl 13-oxo-1-oxa-5-azacyclotridecane-5-carboxylate

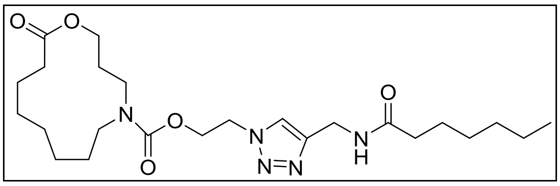

The compound **j** (0.50 g, 1.60 mmol) and *N*-(prop-2-yn-1-yl)pentanamide (0.28 g, 1.68 mmol) were added in a three-necked flask, dissolved in methanol (10ml), then $CuSO_4$ (0.20g, 1.25 mmol) and vitamin C sodium salt(0.25 g, 1.25 mmol) were added in the three-necked flask, and reacted at room temperature for 0.5 h. The mixture rotary evaporated to get dark green viscous liquid, silica gel column chromatography (ethyl acetate), to get a white solid(0.67 g, 1.40 mmol), a yield of 87 %.

^{1}H NMR (300 MHz, $CDCl_3$) δ 7.56 (s, 1H), 6.28 (s, 1H), 4.67-4.56 (m, 2H), 4.54-4.41 (m, 4H), 4.19-4.04 (m, 2H), 3.37-3.04 (m, 4H), 2.47-2.35 (m, 2H), 2.19 (t, *J* = 7.2 Hz, 2H), 1.99-1.87 (m, 1H), 1.83-1.51 (m, 7H), 1.45-1.17 (m, 12H), 0.94-0.78 (m, 3H). ^{13}C NMR (75 MHz, $CDCl_3$) δ 173. 8, 173.3, 155.3, 122.7, 122.7, 63.2, 61.5, 49.7, 49.7, 45.5, 43.7, 36.6, 35.0, 33.7, 31.6, 29.0, 27.9, 25.6, 25.4, 24.8, 24.3, 23.9, 22.6, 14.1. HRMS (m/z) calcd for $C_{24}H_{41}N_5O_5$ [M+H]+ 480.3180, found 480.3174.

Z13-5. 2-(4-(cyclohexanecarboxamidomethyl)-1H-1,2,3-triazol-1-yl)ethyl 13-oxo-1-oxa-5-azacyclotridecane-5-carboxylate

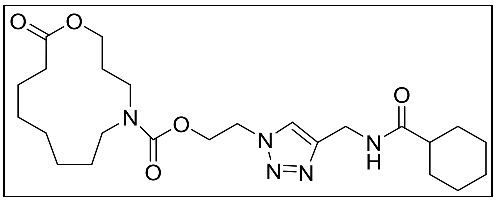

The compound **j** (0.50 g, 1.60 mmol) and *N*-(prop-2-yn-1-yl)cyclohexanecarboxamide (0.28 g, 1.68 mmol) were added in a three-necked flask, dissolved in methanol (10ml), then $CuSO_4$ (0.2 g, 1.25 mmol) and vitamin C sodium salt(0.25 g, 1.25 mmol) were added in the three-necked flask, and reacted at room temperature for 0.5 h. The mixture rotary evaporated to get dark green viscous liquid, silica gel column chromatography (ethyl acetate), to get a white solid(0.65 g, 1.36 mmol), a yield of 85 %.

^{1}H NMR (300 MHz, $CDCl_3$) δ 7.56 (s, 1H), 6.24 (s, 1H), 4.61 (t, *J* = 5.0 Hz, 2H), 4.55-4.41 (m, 4H), 4.19-4.06 (m, 2H), 3.36-3.06 (m, 4H), 2.46-2.36 (m, 2H), 2.16-2.03 (m, 1H), 1.99-1.62 (m, 9H), 1.56 (s, 1H), 1.51-1.14 (m, 12H). ^{13}C NMR (75 MHz, $CDCl_3$) δ 176.1, 173.6, 155.2, 145.0, 122.5, 63.0, 61.3, 49.5, 45.9, 45.3, 45.1, 44.2, 43.5, 34.7, 33.5, 29.6, 29.5, 28.4, 27.7, 25.6, 25.2, 24.6, 24.1, 23.7. HRMS (m/z) calcd for $C_{24}H_{39}N_5O_5$ [M+H]+ 478.3024, found 478.3018.

Z13-6. 2-(4-(((4-bromophenyl)sulfonamido)methyl)-1H-1,2,3-triazol-1-yl)ethyl 13-oxo-1-oxa-5-azacyclotridecane-5-carboxylate

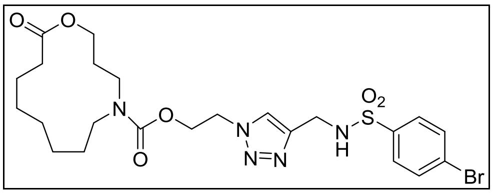

The compound **j** (0.50 g, 1.60 mmol) and 4-bromo-N-(prop-2-yn-1-yl)benzenesulfonamide

(0.46 g, 1.68 mmol) were added in a three-necked flask, dissolved in methanol (10 ml), then $CuSO_4$ (0.2 g, 1.25 mmol) and vitamin C sodium salt(0.25 g, 1.25 mmol) were added in the three-necked flask, and reacted at room temperature for 0.5 h. The mixture rotary evaporated to get dark green viscous liquid, silica gel column chromatography (ethyl acetate), to get a white solid(0.69 g, 1.18 mmol), a yield of 74 %.

^{1}H NMR (300 MHz, DMSO) δ 8.32-8.23 (s, 1H), 7.95-7.86 (m, 1H), 7.82-7.66 (m, 4H), 4.61-4.52 (m, 2H), 4.33-4.23 (m, 2H), 4.10-3.97 (m, 4H), 3.23-2.95 (m, 4H), 2.40-2.30 (m, 2H), 1.83-1.71 (m, 1H), 1.69-1.51 (m, 3H), 1.49-1.15 (m, 8H). ^{13}C NMR (75 MHz, $CDCl_3$) δ 173.8, 155.3, 144.2, 143.8, 139.0, 132.4, 129.4, 128.7, 127.7, 122.9, 63.0, 61.4, 49.7, 46.1, 44.3, 38.6, 33.7, 28.5, 25.7, 25.4, 24.7, 24.2, 23.8. HRMS (m/z) calcd for $C_{23}H_{32}N_5O_6SBr$ [M+H]+ 586.1329, found 586.1326.

Z13-7. 2-(4-phenyl-1H-1,2,3-triazol-1-yl)ethyl 13-oxo-1-oxa-5-azacyclotridecane-5-carboxylate

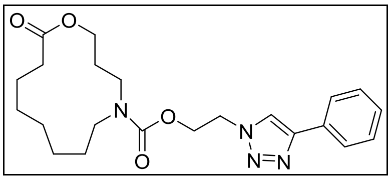

The compound **j** (0.50 g, 1.60 mmol) and ethynylbenzene (0.17 g, 1.68 mmol) were added in a three-necked flask, dissolved in methanol (10 ml), then $CuSO_4$ (0.2 g, 1.25 mmol) and vitamin C sodium salt(0.25 g, 1.25 mmol) were added in the three-necked flask, and reacted at room temperature for 0.5 h. The mixture rotary evaporated to get dark green viscous liquid, silica gel column chromatography (ethyl acetate), to get a white solid(0.63 g, 1.52 mmol), a yield of 95 %.

^{1}H NMR (300 MHz, $CDCl_3$) δ 7.91-7.72 (m, 3H), 7.56-7.24 (m, 3H), 4.77-4.61 (m, 2H), 4.57-4.45 (m, 2H), 4.20-3.96 (m, 2H), 3.38-3.02 (m, 4H), 2.46-2.31 (m, 2H), 2.06-1.59 (m, 4H), 1.59-1.22 (m, 8H). ^{13}C NMR (75 MHz, $CDCl_3$) δ 173.7, 155.3, 147.9, 130.5, 128.9, 128.2, 125.7, 120.0, 63.1, 61.5, 49.6, 45.5, 44.3, 33.6, 27.8, 25.7, 25.3, 24.7, 24.2, 23.7. HRMS (m/z) calcd for $C_{22}H_{30}N_4O_4$ [M+H]+ 414.2267, found 414.2263.

Z13-8. 2-(4-(benzamidomethyl)-1H-1,2,3-triazol-1-yl)ethyl 13-oxo-1-oxa-5-azacyclotridecane-5-carboxylate

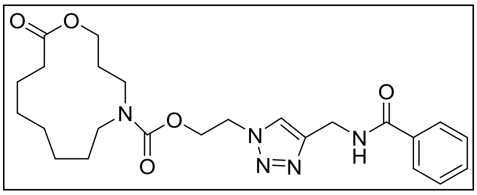

The compound **j** (0.50 g, 1.60 mmol) and *N*-(prop-2-yn-1-yl)benzamide (0.27 g, 1.68 mmol) were added in a three-necked flask, dissolved in methanol (10 ml), then $CuSO_4$ (0.20 g, 1.25 mmol) and vitamin C sodium salt(0.25 g, 1.25 mmol) were added in the three-necked flask, and reacted at room temperature for 0.5 h. The mixture rotary evaporated to get dark green viscous liquid, silica gel column chromatography (ethyl acetate), to get a white solid(0.66 g, 1.40 mmol), a yield of 87 %.

^{1}H NMR (300 MHz, $CDCl_3$) δ 7.84 (m, 2H), 7.72 (m, 1H), 7.61 (s, 1H), 7.43 (m, 3H), 4.70 (m, 2H), 4.61 (t, J = 5.1 Hz, 2H), 4.46 (m 2H), 4.06 (m, 2H), 3.16 (t, J = 32.5 Hz, 4H), 2.45-2.33 (m, 2H), 1.79 (m, 4H), 1.33 (m, 8H). ^{13}C NMR (75 MHz, DMSO) δ 172.8, 166.0, 154.6, 145.2, 134.0, 131.2, 128.2, 128.2, 127.2, 127.2, 123.3, 79.1, 63.1, 63.1, 61.0, 48.7, 48.7, 34.8, 33.0, 25.1, 24.1, 23.6, 23.3. HRMS (m/z) calcd for $C_{24}H_{33}N_5O_5$ [M+H]+ 472.2554, found 472.2550.

Z13-9. 2-(4-((2-chlorobenzamido)methyl)-1H-1,2,3-triazol-1-yl)ethyl 13-oxo-1-oxa-5-azacyclotridecane-5-carboxylate

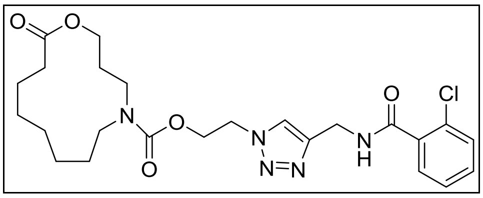

The compound **j** (0.50 g, 1.60 mmol) and 2-chloro-N-(prop-2-yn-1-yl)benzamide (0.33 g, 1.68 mmol) were added in a three-necked flask, dissolved in methanol (10 ml), then $CuSO_4$ (0.20 g, 1.25 mmol) and vitamin C sodium salt(0.25 g, 1.25 mmol) were added in the three-necked flask, and reacted at room temperature for 0.5 h. The mixture rotary evaporated to get dark green viscous liquid, silica gel column chromatography (ethyl acetate), to get a white solid(0.73 g, 1.44 mmol), a yield of 90 %.

^{1}H NMR (300 MHz, $CDCl_3$) δ 7.75-7.69 (m, 1H), 7.62-7.56 (m, 1H), 7.41-7.22 (m, 4H), 4.74-4.67 (m, 2H), 4.61 (t, J = 5.1 Hz, 2H), 4.49-4.41 (m, 2H), 4.15-4.00 (m, 2H), 3.34-3.03 (m, 4H), 2.43-2.31 (m, 2H), 1.96-1.61 (m, 4H), 1.60-1.24 (m, 8H). ^{13}C NMR (75 MHz, $CDCl_3$) δ 173.7, 166.6, 155.3, 144.6, 134.7, 131.4, 130.8, 130.3, 130.0, 127.1, 122.9, 63.1, 61.4, 49.7, 46.0, 44.2, 35.6, 33.6, 27.8, 25.7, 25.3, 24.7, 24.2, 23.7. HRMS (m/z) calcd for $C_{24}H_{32}ClN_5O_5$ [M+H]+ 506.2165, found 506.2161.

Z13-10. 2-(4-((3-chlorobenzamido)methyl)-1H-1,2,3-triazol-1-yl)ethyl 13-oxo-1-oxa-5-azacyclotridecane-5-carboxylate

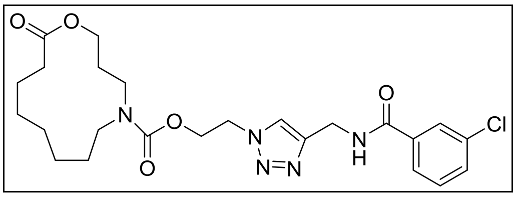

The compound **j** (0.50 g, 1.60 mmol) and 3-chloro-N-(prop-2-yn-1-yl)benzamide (0.33 g, 1.68 mmol) were added in a three-necked flask, dissolved in methanol (10ml), then $CuSO_4$ (0.20 g, 1.25 mmol) and vitamin C sodium salt(0.25 g, 1.25 mmol) were added in the three-necked flask, and reacted at room temperature for 0.5 h. The mixture rotary evaporated to get dark green viscous liquid, silica gel column chromatography (ethyl acetate), to get a white solid(0.73 g, 1.48 mmol), a yield of 90 %.

^{1}H NMR (300 MHz, $CDCl_3$) δ 8.01-7.91(s, 1H), 7.86 (s, 1H), 7.78-7.70 (m, 2H), 7.48-7.42 (m, 1H), 7.38-7.30 (m, 1H), 4.74-4.67 (m, 2H), 4.61 (t, J = 5.1 Hz, 2H), 4.49-4.41 (m, 2H), 4.15-4.00 (m, 2H), 3.34-3.03 (m, 4H), 2.43-2.31 (m, 2H), 1.96-1.61 (m, 4H), 1.60-1.24 (m, 8H). ^{13}C NMR (75 MHz, $CDCl_3$) δ 173.8, 166.2, 155.4, 145.0, 135.9, 134.8, 131.7, 129.9, 127.7, 125.4, 123.5, 63.2, 61.5, 49.8, 46.1, 45.4, 44.4, 43.7, 35.4, 33.7, 25.7, 24.7, 24.3, 23.8. HRMS (m/z) calcd for $C_{24}H_{32}ClN_5O_5$ [M+H]+ 506.2165, found 506.2163.

Z13-11. 2-(4-((4-chlorobenzamido)methyl)-1H-1,2,3-triazol-1-yl)ethyl 13-oxo-1-oxa-5-azacyclotridecane-5-carboxylate

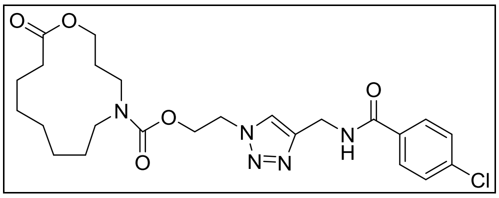

The compound **j** (0.50 g, 1.60 mmol) and 4-chloro-N-(prop-2-yn-1-yl)benzamide (0.33 g, 1.68 mmol) were added in a three-necked flask, dissolved in methanol (10 ml), then $CuSO_4$ (0.20 g, 1.25 mmol) and vitamin C sodium salt(0.25 g, 1.25 mmol) were added in the three-necked flask, and reacted at room temperature for 0.5 h. The mixture rotary evaporated to get dark green viscous liquid, silica gel column chromatography (ethyl acetate), to get a white solid(0.75 g, 1.48 mmol), a yield of 92 %.

^{1}H NMR (300 MHz, $CDCl_3$) δ 8.16-8.05 (m, 1H), 7.88-7.79 (m, 2H), 7.79-7.7.4 (m, 1H), 7.41-7.33 (m, 2H), 4.74-4.67 (m, 2H), 4.61 (t, J = 5.1 Hz, 2H), 4.49-4.41 (m, 2H), 4.15-4.00 (m, 2H), 3.34-3.03 (m, 4H), 2.43-2.31 (m, 2H), 1.96-1.61 (m, 4H), 1.60-1.24 (m, 8H). ^{13}C NMR (75 MHz, $CDCl_3$) δ 173.7, 166.4, 155.3, 145.1, 137.9, 132.4, 129.1, 128.8, 123.4, 63.1, 61.4, 49.7, 46.0, 44.3, 35.3, 33.6, 28.5, 27.7, 25.6, 24.7, 24.2, 23.7. HRMS (m/z) calcd for $C_{24}H_{32}ClN_5O_5$ [M+H]+ 506.2165, found 506.2163.

Z13-12. 5-(1H-1,2,4-triazole-1-carbonyl)-1-oxa-5-azacyclotridecan-13-one

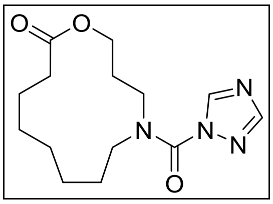

The compound **g** (0.60 g, 2.29 mmol) and 1H-1,2,4-triazole (0.17 g, 2.52 mmol) were added in a dry three-necked flask, dissolved in dichloromethane (10 ml), then Et3N(0.35 g) was added in the three-necked flask, and reacted at room temperature for 5 h. The mixture rotary evaporated to get colorless viscous liquid, silica gel column chromatography (ethyl acetate:Petroleum ether=1:1), to get a white solid(0.61 g, 2.07 mmol), a yield of 90 %.

^{1}H NMR (300 MHz, $CDCl_3$) δ 8.83 (s, 1H), 7.99 (s, 1H), 4.26-4.14 (m, 2H), 3.70-3.32 (m, 4H), 2.48-2.39 (m, 2H), 2.31-2.06 (m, 2H), 1.93-1.63 (m, 4H), 1.56-1.36 (m, 6H). ^{13}C NMR (75 MHz, $CDCl_3$) δ 173.7, 152.1, 149.2, 146.5, 61.4, 47.7, 45.6, 33.6, 28.9, 27.3, 25.4, 24.8, 24.4, 23.6. HRMS (m/z) calcd for $C_{14}H_{22}N_4O_3$ [M+H]+ 295.1765, found 295.1760.

Z13-13. 2-(4-cyclohexyl-1H-1,2,3-triazol-1-yl)ethyl 13-oxo-1-oxa-5-azacyclotridecane-5-carboxylate

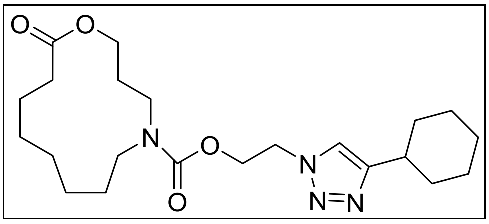

The compound **j** (0.50 g, 1.60 mmol) and ethynylcyclohexane (0.19 g, 1.76 mmol) were added in a three-necked flask, dissolved in methanol (10 ml), then $CuSO_4$ (0.20 g, 1.25 mmol) and vitamin C sodium salt(0.25 g, 1.25 mmol) were added in the three-necked flask, and reacted at room temperature for 0.5 h. The mixture rotary evaporated to get dark green viscous liquid, silica gel column chromatography (ethyl acetate), to get a white solid(0.65 g, 1.55 mmol), a yield of 96 %.

^{1}H NMR (300 MHz, $CDCl_3$) δ 7.24 (s, 1H), 4.57 (t, J = 5.2 Hz, 2H), 4.44 (m, 2H), 4.19 – 3.98 (m, 2H), 3.36 – 3.01 (m, 4H), 2.82 – 2.66 (m, 1H), 2.43 – 2.34 (m, 2H), 2.10 – 1.85 (m, 4H), 1.84 – 1.49 (m, 7H), 1.49 – 1.16 (m, 13H). ^{13}C NMR (75 MHz, $CDCl_3$) δ 173.7, 155.42, 153.9, 119.8, 63.3, 61.4, 49.4, 46.0, 45.4, 44.3, 43.6, 35.3, 33.7, 33.1, 28.2, 27.6, 26.2, 26.1, 25.8, 24.7, 24.3, 23.8. HRMS

(m/z) calcd for $C_{22}H_{36}N_4O_4$ [M+H]+, 421.2809 found 421.2802.

Z13-14. 2-(4-cyclohexyl-1H-1,2,3-triazol-1-yl)ethyl 13-oxo-1-oxa-5-azacyclotridecane-5-carboxylate

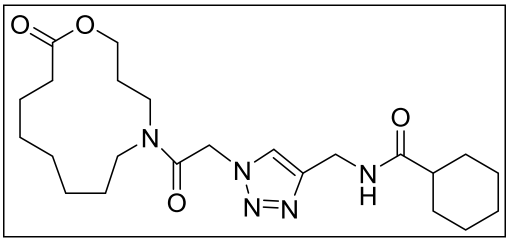

The compound **j** (0.50 g, 1.77 mmol) and *N*-(prop-2-yn-1-yl)cyclohexanecarboxamide (0.31 g, 1.86 mmol) were added in a three-necked flask, dissolved in methanol (10 ml), then $CuSO_4$ (0.20 g, 1.25 mmol) and vitamin C sodium salt(0.25 g, 1.25 mmol) were added in the three-necked flask, and reacted at room temperature for 0.5 h. The mixture rotary evaporated to get dark green viscous liquid, silica gel column chromatography (ethyl acetate), to get a white solid(0.73 g, 1.63 mmol), a yield of 92 %.

^{1}H NMR (300 MHz, $CDCl_3$) δ 7.69 (m, 1H), 6.32 (m, 1H), 5.19 (s, 2H), 4.51 (m, 2H), 4.26 – 4.10 (m, 2H), 3.37 (m, 4H), 2.44 (m, 2H), 2.18 – 1.90 (m, 4H), 1.90 – 1.54 (m, 9H), 1.53 – 1.13 (m, 10H).

^{13}C NMR (75 MHz, $CDCl_3$) δ 176.2, 173.6, 164.4, 145.1, 123.9, 61.6, 60.9, 50.8, 46.0, 45.3, 43.7, 43.3, 35.0, 33.6, 29.6, 28.6, 27.6, 25.9, 25.7, 24.8, 24.4, 23.7, 23.5.

HRMS (m/z) calcd for $C_{23}H_{37}N_5O_4$ [M+H]+, 448.2918 found 448.2912.

e. 1-oxa-4-azacyclohexadecan-16-one

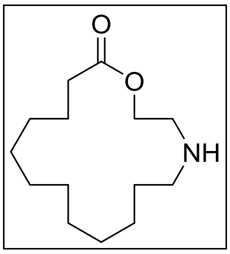

Compound **e** was synthesized in 87% yield according to the procedure reported in Ref [1]. ^{1}H-NMR (300 MHz, $CDCl_3$): δ 4.36-4.11 (m, 2H), 2.96-2.83 (m, 2H), 2.74-2.62 (m, 2H), 2.44-2.27 (m, 2H), 1.88-1.64 (m, 2H), 1.57-1.26 (m, 10H). ^{13}C-NMR (75 MHz, $CDCl_3$): δ 173.7, 62.9, 46.5, 46.2, 34.5, 25. 7, 25.2, 25.0, 23.0, 22.7.

h. 16-oxo-1-oxa-4-azacyclohexadecane-4-carbonyl chloride

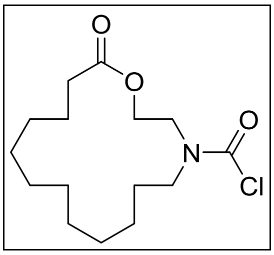

Under the ice water bath, the triphosgene (9.83 g, 33.14 mmol) was added in a dry three-necked flask, dissolved in dichloromethane (25 mL). And the compound **e** (20 g, 82.86 mmol) and Et_3N (8.38 g, 82.86 mmol) dissolved in dichloromethane (15 mL) were added dropwise. Then they reacted at room temperature for 10 h. The mixture rotary evaporated to get colorless viscous liquid, silica gel column chromatography (ethyl acetate : Petroleum ether=1:5), to get a colorless viscous liquid (19.41 g, 63.85 mmol), a yield of 77 %. The compound used for the next reaction directly.

k. 2-azidoethyl 16-oxo-1-oxa-4-azacyclohexadecane-4-carboxylate

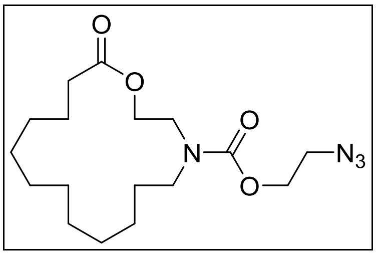

The compound **h** (15 g, 49.37 mmol) and 2-azidoethan-1-ol (4.73 g, 54.31 mmol) were added in a dry three-necked flask, dissolved in dichloromethane (25 ml), then Et_3N(5 g, 49.37 mmol) was added in the three-necked flask, and reacted at room temperature for 18 h. The mixture rotary evaporated to get dark brown viscous liquid, silica gel column chromatography (ethyl acetate : Petroleum ether=1:5), to get a white solid(15.70 g, 44.29 mmol), a yield of 89 %. ^{1}H NMR (300 MHz, $CDCl_3$) δ 4.29-4.14 (m, 4H), 3.55-3.37 (m, 4H), 3.33-3.19 (m, 2H), 2.29 (t, J = 7.1 Hz, 2H), 1.69-1.47 (m, 4H), 1.40-1.19 (m, 14H).

Z16-1. 2-(4-(propionamidomethyl)-1H-1,2,3-triazol-1-yl)ethyl 16-oxo-1-oxa-4-azacyclohexadecane-4-carboxylate

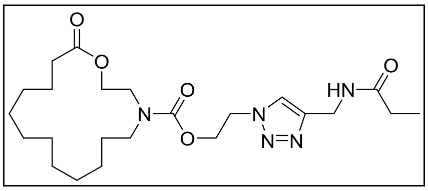

The compound **k** (0.50 g, 1.41 mmol) and *N*-(prop-2-yn-1-yl)propionamide (0.16 g, 1.48 mmol) were added in a three-necked flask, dissolved in methanol (10 ml), then $CuSO_4$ (0.20 g, 1.25 mmol) and vitamin C sodium salt(0.25 g, 1.25 mmol) were added in the three-necked flask, and reacted at room temperature for 0.5 h. The mixture rotary evaporated to get dark green viscous liquid, silica gel column chromatography (ethyl acetate), to get a white solid(0.58 g, 1.35 mmol), a yield of 88 %.

^{1}H NMR (300 MHz, $CDCl_3$) δ 7.60 (s, 1H), 6.66-6.39 (m, 1H), 4.62 (t, *J* = 5.1 Hz, 2H), 4.55-4.43 (m, 4H), 4.27-4.19 (m, 1H), 4.01-3.93 (m, 1H), 3.53-3.35 (m, 2H), 3.33-3.13 (m, 2H), 2.39-2.28 (m, 2H), 2.28-2.19 (m, 2H), 1.75-1.61 (s, 2H), 1.60-1.40 (m, 2H), 1.40-1.22 (m, 12H), 1.15 (t, *J* = 7.6 Hz, 3H). ^{13}C NMR (75 MHz, $CDCl_3$) δ 173.9, 173.6, 154.9, 144.9, 122.6, 63.6, 63.2, 49.5, 49.4, 49.2, 48.5, 47.5, 46.7, 34.7, 33.7, 29.6, 29.3, 27.1, 26.4, 26.0, 24.8, 23.7, 9.6. HRMS (m/z) calcd for $C_{23}H_{39}N_5O_5$ [M+H]+ 414.2267, found 414.2263.

Z16-2. 2-(4-(butyramidomethyl)-1H-1,2,3-triazol-1-yl)ethyl 16-oxo-1-oxa-4-azacyclohexadecane-4-carboxylate

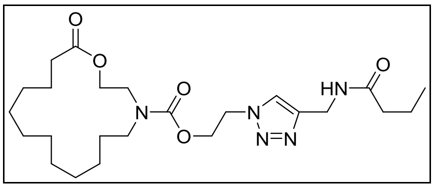

The compound **k** (0.50 g, 1.41 mmol) and *N*-(prop-2-yn-1-yl)butyramide (0.19 g, 1.48 mmol) were added in a three-necked flask, dissolved in methanol (10ml), then $CuSO_4$ (0.20 g, 1.25 mmol) and vitamin C sodium salt(0.25 g, 1.25 mmol) were added in the three-necked flask, and reacted at room temperature for 0.5 h. The mixture rotary evaporated to get dark green viscous liquid, silica gel column chromatography (ethyl acetate), to get a white solid(0.55 g, 1.15 mmol), a yield of 81 %.

^{1}H NMR (300 MHz, $CDCl_3$) δ 7.63 (s, 1H), 6.84 (d, *J* = 28.8 Hz, 1H), 4.62 (t, *J* = 5.2 Hz, 2H), 4.54-4.44 (m, 4H), 4.28-4.19 (m, 1H), 4.02-3.94 (m, 1H), 3.54-3.34 (m, 2H), 3.33-3.14 (m, 2H), 2.38-2.29 (m, 2H), 2.18 (t, *J* = 7.5 Hz, 2H), 1.73-1.60 (m, 4H), 1.60-1.41 (m, 2H), 1.40-1.22 (m, 16H), 0.92 (m, *J* = 7.4 Hz, 3H). ^{13}C NMR (75 MHz, $CDCl_3$) δ 173.6, 173.2, 155.4, 145.4, 122.7, 63.7, 63.2, 49.4, 48.5, 48.2, 46.8, 38.3, 34.8, 33.8, 27.2, 26.86, 26.4, 26.1, 26.0, 24.8, 23.8, 22.6, 19.0, 13.7. HRMS (m/z) calcd for $C_{24}H_{41}N_5O_5$ [M+H]+ 480.3180, found 480.3175.

Z16-3. 2-(4-(pentanamidomethyl)-1H-1,2,3-triazol-1-yl)ethyl 16-oxo-1-oxa-4-azacyclohexadecane-4-carboxylate

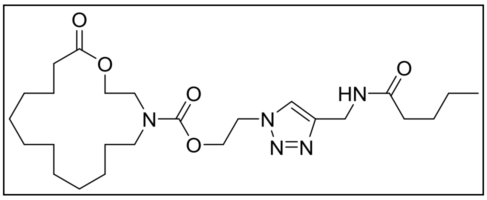

The compound **k** (0.50 g, 1.41 mmol) and *N*-(prop-2-yn-1-yl)pentanamide (0.21 g, 1.48 mmol)

were added in a three-necked flask, dissolved in methanol (10ml), then $CuSO_4$ (0.20 g, 1.25 mmol) and vitamin C sodium salt(0.25 g, 1.25 mmol) were added in the three-necked flask, and reacted at room temperature for 0.5 h. The mixture rotary evaporated to get dark green viscous liquid, silica gel column chromatography (ethyl acetate), to get a white solid(0.61 g, 1.24 mmol), a yield of 87 %.

^{1}H NMR (300 MHz, $CDCl_3$) δ 7.57 (s, 1H), 6.51-6.19 (m, 1H), 4.61 (t, *J* = 5.2 Hz, 2H), 4.55-4.43 (m, 4H), 4.29-4.17 (m, 1H), 4.01-3.92 (m, 1H), 3.55-3.44 (m, 1H), 3.43-3.34 (m, 1H), 3.33-3.11 (m, 2H), 2.39-2.28 (m, 2H), 2.20 (t, *J* = 7.7 Hz, 2H), 1.78-1.51 (m, 6H), 1.40-1.18 (m, 16H), 0.90 (t, *J* = 7.1 Hz, 3H). ^{13}C NMR (75 MHz, $CDCl_3$) δ 173.8, 173.4, 145.4, 130.9, 122.6, 63.8, 63.3, 58.4, 49.6, 48.6, 47.7, 36.3, 34.9, 33.9, 32.0, 29.7, 27.7, 27.3, 26.5, 26.2, 24.9, 23.9, 22.4, 18.5, 13.8. HRMS (m/z) calcd for $C_{25}H_{43}N_5O_5$ [M+H]+ 494.3337, found 494.3331.

Z16-4. 2-(4-(heptanamidomethyl)-1H-1,2,3-triazol-1-yl)ethyl 16-oxo-1-oxa-4-azacyclohexadecane-4-carboxylate

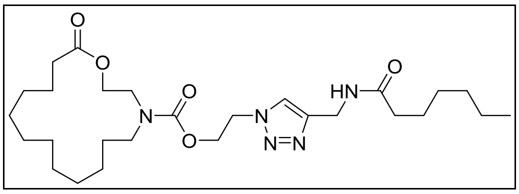

The compound **k** (0.50 g, 1.41 mmol) and *N*-(prop-2-yn-1-yl)heptanamide (0.25 g, 1.48 mmol) were added in a three-necked flask, dissolved in methanol (10 ml), then $CuSO_4$ (0.20 g, 1.25 mmol) and vitamin C sodium salt(0.25 g, 1.25 mmol) were added in the three-necked flask, and reacted at room temperature for 0.5 h. The mixture rotary evaporated to get dark green viscous liquid, silica gel column chromatography (ethyl acetate), to get a white solid(0.68 g, 1.30 mmol), a yield of 92 %.

^{1}H NMR (300 MHz, $CDCl_3$) δ 7.59 (s, 1H), 6.62-6.35 (m, 1H), 4.61 (t, *J* = 5.1 Hz, 2H), 4.55-4.43 (m, 4H), 4.25-4.19 (m, 1H), 4.01-3.93 (s, 1H), 3.84-3.34 (m, 2H), 3.34-3.13 (m, 2H), 2.39-2.27 (m, 2H), 2.19 (t, *J* = 7.6 Hz, 2H), 1.74-1.50 (m, 6H), 1.41-1.18 (m, 20H), 0.87 (t, *J* = 6.8 Hz, 3H). ^{13}C NMR (75 MHz, $CDCl_3$) δ 173.5, 173.4, 155.0, 145.2, 122.7, 63.8, 63.3, 49.6, 49.3, 48.6, 47.7, 46.9, 36.6, 34.9, 33.9, 31.6, 29.7, 29.0, 27.3, 26.6, 26.2, 26.1, 25.6, 24.9, 24.0, 22.5, 14.0. HRMS (m/z) calcd for $C_{27}H_{47}N_5O_5$ [M+H]+ 522.365, found 522.3645.

Z16-5. 2-(4-(cyclohexanecarboxamidomethyl)-1H-1,2,3-triazol-1-yl)ethyl 16-oxo-1-oxa-4-azacyclohexadecane-4-carboxylate

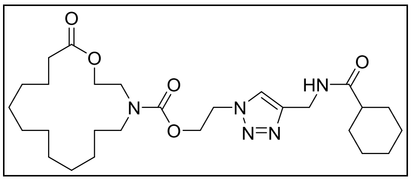

The compound **k** (0.50 g, 1.41 mmol) and *N*-(prop-2-yn-1-yl)heptanamide (0.24 g, 1.48 mmol) were added in a three-necked flask, dissolved in methanol (10 ml), then $CuSO_4$ (0.20 g, 1.25 mmol) and vitamin C sodium salt(0.25 g, 1.25 mmol) were added in the three-necked flask, and reacted at room temperature for 0.5 h. The mixture rotary evaporated to get dark green viscous liquid, silica gel column chromatography (ethyl acetate), to get a white solid(0.65 g, 1.25 mmol), a yield of 88 %.

^{1}H NMR (300 MHz, $CDCl_3$) δ 7.55 (s, 1H), 6.40-6.14 (d, *J* = 50.2 Hz, 1H), 4.61 (t, *J* = 5.1 Hz, 2H), 4.53-

4.45 (m, 4H), 4.27-4.19 (m, 1H), 4.02-3.94 (m, 1H), 3.53-3.45 (m, 1H), 3.42-3.34 (m, 1H), 3.33-3.13 (m, 2H), 2.39-2.28 (m, 2H), 2.16-2.05 (m, 1H), 1.92-1.61 (m, 8H), 1.51-1.23 (m, 20H). ^{13}C NMR (75 MHz, $CDCl_3$) δ 176.3, 173.7, 155.0, 145.4, 122.5, 63.5, 63.3, 49.6, 49.2, 48.6, 47.7, 46.8, 45.2, 34.8, 33.8, 29.6, 27.5, 27.2, 26.9, 26.9, 26.2, 26.1, 26.0, 25.7, 25.7, 24.9, 23.9. HRMS (m/z) calcd for $C_{27}H_{45}N_5O_5$ [M+H]+ 520.3493, found 520.3491.

Z16-6. 2-(4-(((4-bromophenyl)sulfonamido)methyl)-1H-1,2,3-triazol-1-yl)ethyl 16-oxo-1-oxa-4-azacyclohexadecane-4-carboxylate

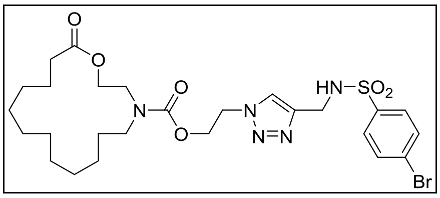

The compound **k** (0.50 g, 1.41 mmol) and *N*-(prop-2-yn-1-yl)benzamide (0.41 g, 1.48 mmol) were added in a three-necked flask, dissolved in methanol (10 ml), then $CuSO_4$ (0.20 g, 1.25 mmol) and vitamin C sodium salt(0.25 g, 1.25 mmol) were added in the three-necked flask, and reacted at room temperature for 0.5 h. The mixture rotary evaporated to get dark green viscous liquid, silica gel column chromatography (ethyl acetate), to get a white solid(0.71 g, 1.13 mmol), a yield of 80 %.

^{1}H NMR (300 MHz, DMSO) δ 8.27 (s, 1H), 7.91 (s, 1H), 7.81-7.66 (m, 4H), 4.63-4.53 (m, 2H), 4.37-4.26 (m, 2H), 4.16-3.94 (m, 4H), 3.29-3.36 (m, 2H), 3.22-3.04 (m, 2H), 2.29 (t, *J* = 6.8 Hz, 2H), 1.64-1.39 (m, 4H), 1.37-1.10 (m, 14H). ^{13}C NMR (75 MHz, $CDCl_3$) δ 174.3, 155.0, 144.3, 139.1, 132.4, 129.8, 128.7, 127.6, 123.0, 122.9, 64.0, 63.3, 49.6, 46.7, 38.7, 38.5, 34.0, 33.8, 27.5, 27.2, 27.0, 26.5, 26.1, 24.9, 24.0, 23.8. HRMS (m/z) calcd for $C_{26}H_{38}BrN_5O_6S$ [M+H]+ 626.1653, found 626.1655.

Z16-7. 2-(4-phenyl-1H-1,2,3-triazol-1-yl)ethyl 16-oxo-1-oxa-4-azacyclohexadecane-4-carboxylate

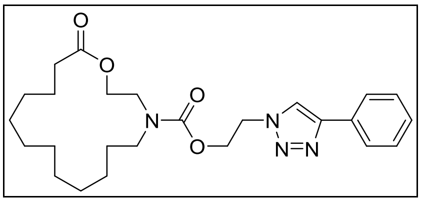

The compound **k** (0.50 g, 1.41 mmol) and ethynylbenzene (0.15 g, 1.48 mmol) were added in a three-necked flask, dissolved in methanol (10 ml), then $CuSO_4$ (0.20 g, 1.25 mmol) and vitamin C sodium salt(0.25 g, 1.25 mmol) were added in the three-necked flask, and reacted at room temperature for 0.5 h. The mixture rotary evaporated to get dark green viscous liquid, silica gel column chromatography (ethyl acetate), to get a white solid(0.61 g, 1.34 mmol), a yield of 95 %.

^{1}H NMR (300 MHz, $CDCl_3$) δ 7.83 (m, 3H), 7.47-7.39 (m, 2H), 7.38-7.30 (m, 1H), 4.70 (t, *J* = 5.1 Hz, 2H), 4.55 (t, *J* = 5.2 Hz, 2H), 4.25 (m, 1H), 4.12 (m, 1H), 3.50 (m, 1H), 3.40 (m, 1H), 3.33-3.13 (m, 2H), 2.31 (m, 2H), 1.66 (m, 2H), 1.59-1.38 (m, 2H), 1.31 (m, 14H). ^{13}C NMR (75 MHz, $CDCl_3$) δ 173.6, 155.3, 147.9, 130.5, 129.0, 129.0, 128.7, 128.2, 125.7, 120.1, 63.3, 49.6, 48.9, 47.7, 46.8, 33.8, 27.6, 27.0, 26.5, 26.4, 26.30, 26.0, 25.9, 24.8, 23.8. HRMS (m/z) calcd for $C_{25}H_{36}N_4O_4$ [M+H]+ 456.2737, found 456.2731.

Z16-8. 2-(4-(benzamidomethyl)-1H-1,2,3-triazol-1-yl)ethyl 16-oxo-1-oxa-4-azacyclohexadecane-4-carboxylate

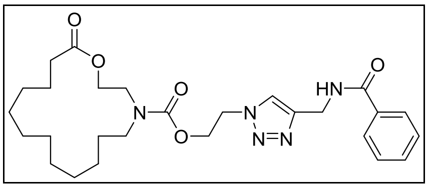

The compound **k** (0.50 g, 1.41 mmol) and *N*-(prop-2-yn-1-yl)benzamide (0.24 g, 1.48 mmol) were added in a three-necked flask, dissolved in methanol (10 ml), then $CuSO_4$ (0.20 g, 1.25 mmol) and vitamin C sodium salt(0.25 g, 1.25 mmol) were added in the three-necked flask, and reacted at room temperature for 0.5 h. The mixture rotary evaporated to get dark green viscous liquid, silica gel column chromatography (ethyl acetate), to get a white solid(0.62 g, 1.21 mmol), a yield of 85 %.

^{1}H NMR (300 MHz, DMSO) δ 9.01 (s, 1H), 8.04-7.79 (m, 3H), 7.61-7.34 (m, 3H), 4.68-4.44 (m, 4H), 4.40-4.26 (m, 2H), 4.14-3.89 (m, 2H), 3.45-3.20 (m, 2H), 3.18-2.96 (m, 2H), 2.36-2.17 (m, 2H), 1.65-1.48 (m, 2H), 1.45-1.02 (m, 14H). ^{13}C NMR (75 MHz, $CDCl_3$) δ 173.6, 167.3, 154.9, 145.3, 133.9, 131.5, 128.4, 127.0, 122.9, 63.4, 63.2, 49.1, 48. 5, 46.6, 35.3, 33.7, 27.1, 26.8, 26.3, 26.0, 24.7, 23.8. HRMS (m/z) calcd for $C_{27}H_{39}N_5O_5$ [M+H]+ 514.3024, found 514.3020.

Z16-9. 2-(4-((2-chlorobenzamido)methyl)-1H-1,2,3-triazol-1-yl)ethyl 16-oxo-1-oxa-4-azacyclohexadecane-4-carboxylate

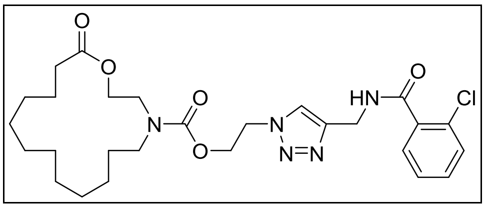

The compound **k** (0.50 g, 1.41 mmol) and 2-chloro-*N*-(prop-2-yn-1-yl)benzamide (0.29 g, 1.48 mmol) were added in a three-necked flask, dissolved in methanol (10 ml), then $CuSO_4$ (0.20 g, 1.25 mmol) and vitamin C sodium salt(0.25 g, 1.25 mmol) were added in the three-necked flask, and reacted at room temperature for 0.5 h. The mixture rotary evaporated to get dark green viscous liquid, silica gel column chromatography (ethyl acetate), to get a white solid(0.71 g, 1.30 mmol), a yield of 92 %.

^{1}H NMR (300 MHz, $CDCl_3$) δ 7.71 (s, 1H), 7.60 (m, 1H), 7.41-7.25 (m, 4H), 4.72 (m, 2H), 4.63 (t, *J* = 5.1 Hz, 2H), 4.49 (m, 2H), 4.21 (m, 1H), 3.93 (m, 1H), 3.51-3.41 (m, 1H), 3.36 (m, 1H), 3.30-3.12 (m, 2H), 2.27 (m, 2H), 1.72-1.40 (m, 4H), 1.30 (m, 14H). ^{13}C NMR (75 MHz, $CDCl_3$) δ 173.7, 166.6, 155.0, 145.0, 135.1, 131.2, 130.83 130.2, 129.8, 127.0, 122.8, 63.7, 63.2, 49.5, 49.2, 48.6, 47.6, 46.8, 35.6, 33.8, 27.5, 27.2, 26.9, 26.5, 26.1, 24.9, 23.8. HRMS (m/z) calcd for $C_{27}H_{38}ClN_5O_5$ [M+H]+ 548.2634, found 548.2631.

Z16-10. 2-(4-((3-chlorobenzamido)methyl)-1H-1,2,3-triazol-1-yl)ethyl 16-oxo-1-oxa-4-azacyclohexadecane-4-carboxylate

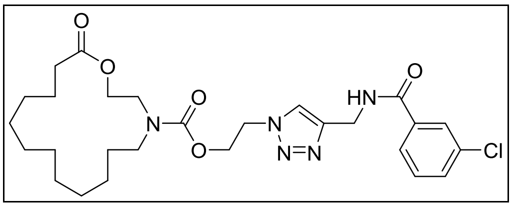

The compound **k** (0.50 g, 1.41 mmol) and 3-chloro-*N*-(prop-2-yn-1-yl)benzamide (0.29 g, 1.48 mmol) were added in a three-necked flask, dissolved in methanol (10ml), then $CuSO_4$ (0.20 g,

1.25 mmol) and vitamin C sodium salt(0.25 g, 1.25 mmol) were added in the three-necked flask, and reacted at room temperature for 0.5 h. The mixture rotary evaporated to get dark green viscous liquid, silica gel column chromatography (ethyl acetate), to get a white solid(0.70 g, 1.28 mmol), a yield of 90 %.

^{1}H NMR (300 MHz, $CDCl_3$) δ 7.86 (s, 1H), 7.70 (m, 2H), 7.61-7.31 (m, 3H), 4.72 (m, 2H), 4.63 (t, *J* = 5.1 Hz, 2H), 4.49 (m, 2H), 4.22 (m, 1H), 3.91 (m, 1H), 3.41 (m, 2H), 3.22 (m, 2H), 2.40-2.21 (m, 2H), 1.60 (m, 4H), 1.28 (m, 14H). ^{13}C NMR (75 MHz, $CDCl_3$) δ 173.6, 166.1, 155.0, 135.9, 134.6, 133.0, 131.5, 129.8, 127.6, 125.3, 123.3, 63.7, 63.2, 49.7, 49.6, 49.2, 48.6, 47.7, 46.7, 35.4, 33.8, 29.7, 27.2, 26.5, 26.2, 24.9, 23.9. HRMS (m/z) calcd for $C_{27}H_{38}ClN_5O_5$ [M+H]+ 548.2634, found 548.2631.

Z16-11. 2-(4-((4-chlorobenzamido)methyl)-1H-1,2,3-triazol-1-yl)ethyl 16-oxo-1-oxa-4-azacyclohexadecane-4-carboxylate

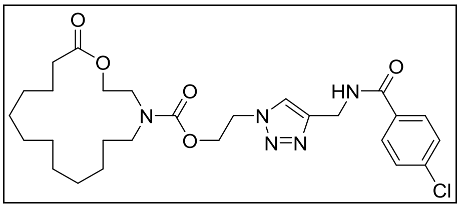

The compound **k** (0.50 g, 1.41 mmol) and 4-chloro-*N*-(prop-2-yn-1-yl)benzamide (0.29 g, 1.48 mmol) were added in a three-necked flask, dissolved in methanol (10ml), then $CuSO_4$ (0.20 g, 1.25 mmol) and vitamin C sodium salt(0.25 g, 1.25 mmol) were added in the three-necked flask, and reacted at room temperature for 0.5 h. The mixture rotary evaporated to get dark green viscous liquid, silica gel column chromatography (ethyl acetate), to get a white solid(0.71 g, 1.30 mmol), a yield of 92 %.

^{1}H NMR (300 MHz, $CDCl_3$) δ 7.99-7.54 (m, 4H), 7.38 (m, 2H), 4.70 (m, 2H), 4.62 (m, 2H), 4.50 (m, 2H), 4.07 (m, 2H), 3.31 (m, 4H), 2.46-2.15 (m, 2H), 1.59 (m, 4H), 1.28 (m, 14H). ^{13}C NMR (75 MHz, $CDCl_3$) δ 173.8, 166.4, 155.4, 155.0, 145.3, 137.8, 132.4, 128.7, 123.1, 63.7, 63.2, 49.6, 49.2, 47.6, 35.4, 33.8, 27.22, 26.9, 26.5, 26.1, 24.8, 23.9. HRMS (m/z) calcd for $C_{27}H_{38}ClN_5O_5$ [M+H]+ 548.2634, found 548.2632.

Z16-12. 4-(1H-1,2,4-triazole-1-carbonyl)-1-oxa-4-azacyclohexadecan-16-one

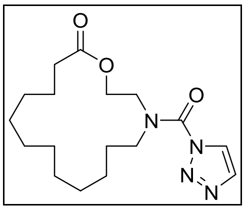

The compound **k** (0.60 g, 1.97 mmol) and 1H-1,2,4-triazole (0.15 g, 2.17 mmol) were added in a dry three-necked flask, dissolved in dichloromethane (10 ml), then Et_3N(0.33 g) was added in the three-necked flask, and reacted at room temperature for 5 h. The mixture rotary evaporated to get colorless viscous liquid, silica gel column chromatography (ethyl acetate:Petroleum ether=1:1), to get a white solid(0.57 g, 1.69 mmol), a yield of 85 %.

^{1}H NMR (300 MHz, $CDCl_3$) δ 8.83 (s, 1H), 7.99 (s, 1H), 4.46 (t, *J* = 4.9 Hz, 2H), 3.92-3.73 (m, 2H), 3.73-3.55 (m, 2H), 2.36 (t, *J* = 7.2 Hz, 2H), 1.82-1.63 (m, 4H), 1.45-1.31 (m, 14H). ^{13}C NMR (75 MHz, $CDCl_3$) δ 173.5, 152.2, 149.4, 146.7, 50.7, 48.9, 43.7, 33.9, 29.7, 27.2, 26.4, 26.1, 26.1, 25.9, 24.9, 23.9. HRMS (m/z) calcd for $C_{17}H_{28}N_4O_3$ [M+H]+ 337.2234, found 337.2229.

Z16-13. 2-(4-cyclohexyl-1H-1,2,3-triazol-1-yl)ethyl 16-oxo-1-oxa-4-azacyclohexadecane-4-carboxylate

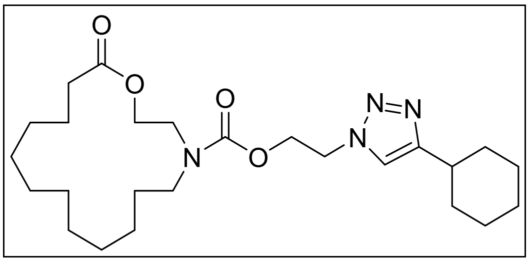

The compound **k** (0.50 g, 1.41 mmol) and ethynylcyclohexane (0.18 g, 1.55 mmol) were added in a three-necked flask, dissolved in methanol (10ml), then $CuSO_4$ (0.20 g, 1.25 mmol) and vitamin C sodium salt(0.25 g, 1.25 mmol) were added in the three-necked flask, and reacted at room temperature for 0.5 h. The mixture rotary evaporated to get dark green viscous liquid, silica gel column chromatography (ethyl acetate), to get a white solid(0.63 g, 1.36 mmol), a yield of 96 %. ^{1}H NMR (300 MHz, $CDCl_3$) δ 7.77 – 7.49 (m, 1H), 4.65-4.59 (m, 2H), 4.48 (t, *J* = 5.0 Hz, 2H), 4.26 (s, 1H), 4.09 (s, 1H), 3.55-3.11 (m, 4H), 2.79 (m, 1H), 2.40-2.28 (m, 2H), 2.13-1.96 (m, 2H), 1.89 – 1.50 (m, 6H), 1.51 – 1.14 (m, 22H). ^{13}C NMR (75 MHz, $CDCl_3$) δ 173.6, 155.5, 153.9, 119.8, 63.5, 49.4, 49.3, 48.8, 47.64, 46.8, 35.3, 33.8, 33.1, 27.5, 27.3, 26.81, 26.1, 24.9, 24.9, 24.8, 24.5, 23.9. HRMS (m/z) calcd for $C_{25}H_{42}N_4O_4$ [M+H]+ 463.3279, found 463.3272.

Z16-14. *N*-((1-(2-oxo-2-(16-oxo-1-oxa-4-azacyclohexadecan-4-yl)ethyl)-1H-1,2,3-triazol-4-yl)methyl)cyclohexanecarboxamide

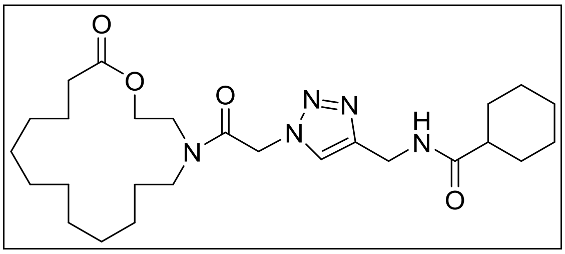

The compound **q** (0.50 g, 1.54 mmol) and *N*-(prop-2-yn-1-yl)cyclohexanecarboxamide (0.27 g, 1.62 mmol) were added in a three-necked flask, dissolved in methanol (10ml), then $CuSO_4$ (0.20 g, 1.25 mmol) and vitamin C sodium salt(0.25 g, 1.25 mmol) were added in the three-necked flask, and reacted at room temperature for 0.5 h. The mixture rotary evaporated to get dark green viscous liquid, silica gel column chromatography (ethyl acetate), to get a white solid(0.71 g, 1.36 mmol), a yield of 94 %. ^{1}H NMR (300 MHz, $CDCl_3$) δ 7.67 (s, 1H), 6.30 (s, 1H), 5.29-5.17 (m, 2H), 4.50 (d, *J* = 5.4 Hz, 2H), 4.26 – 4.19 (m, 2H), 3.71-3.53 (m, 2H), 3.48 – 3.33 (m, 2H), 2.43 – 2.28 (m, 2H), 2.15-2.02 (m, 1H), 1.90 – 1.50 (m, 8H), 1.50 – 1.10 (m, 20H). ^{13}C NMR (75 MHz, $CDCl_3$) δ 176.2, 173.5, 165.0, 145.1, 123.9, 63.0, 62.6, 51.1, 50.7, 49.0, 48.4, 47.3, 46.6, 45.2, 34.9, 33.7, 29.5, 27.7, 27.4, 26.9, 26.9, 25.5, 25.0, 23.9.

HRMS (m/z) calcd for $C_{26}H_{43}N_5O_4$ [M+H]+ 490.3388, found 490.3380.

Table S1 Inhibition rate of **Z12** and AMZ (positive control)

**Comp.**	C.(mg/L)	**Enzyme activity U**	**ΔU**	Inhibition rate %
AZM	200	0.04	1.375	97.17
	100	0.054	1.361	96.18
	50	0.043	1.372	96.96
	25	0.099	1.316	93.00
	12.5	0.303	1.112	78.59
Z12-1	200	0.659	0.901	57.76
	100	0.635	0.925	59.29
	50	1.469	0.091	5.83
Z12-2	200	1.373	0.205	12.99
	100	1.663	-0.085	-5.39
	50	1.354	0.224	14.2
Z12-3	200	0.512	1.048	67.18
	100	1.088	0.472	30.26
	50	1.316	0.244	15.64
Z12-4	200	1.552	-0.152	-10.86
	100	1.914	-0.514	-36.71
	50	1.939	-0.539	-38.5
Z12-5	200	0.572	1.035	64.41
	100	0.616	0.991	61.67
	50	1.01	0.597	37.15
Z12-6	200	1.996	0.401	16.73
	100	2.367	0.03	1.25
	50	2.275	0.122	5.09
Z12-7	200	2.19	0.207	8.64
	100	2.058	0.339	14.14
	50	2.054	0.343	14.31
Z12-8	200	1.36	0.2	12.82
	100	1.256	0.304	19.49
	50	1.34	0.22	14.1
Z12-9	200	1.597	-0.019	-1.2
	100	1.458	0.12	7.6
	50	1.496	0.082	5.2
Z12-10	200	0.889	0.511	36.5
	100	1.152	0.248	17.71
	50	1.14	0.26	18.57
Z12-11	200	1.893	-0.493	-35.21
	100	1.63	-0.23	-16.43
	50	1.958	-0.558	-39.86
Z12-12	200	1.587	-0.187	-13.36
	100	1.52	-0.12	-8.57

	50	1.44	-0.04	-2.86
	200	0.274	0.090	-48.3
Z12-13	100	0.333	0.145	-77.6
	50	0.295	0.107	-57.3
	200	0.224	0.037	-19.6
Z12-14	100	0.248	0.060	-32.2
	50	0.241	0.054	-28.7

Table S2 Inhibition rate of **Z13**

**Comp.**	C.（mg/L）	**Enzyme activity U**	**ΔU**	Inhibition rate %
	200	1.723	-0.145	-9.19
Z13-1	100	1.547	0.031	1.96
	50	1.431	0.147	9.32
	200	1.167	0.53	31.23
Z13-2	100	0.959	0.738	43.49
	50	1.345	0.352	20.74
	200	1.723	-0.145	-9.19
Z13-3	100	1.495	0.083	5.26
	50	1.785	-0.207	-13.12
	200	0.759	0.641	45.79
Z13-4	100	1.369	0.031	2.21
	50	1.67	-0.27	-19.29
	200	0.735	0.872	54.26
Z13-5	100	0.818	0.789	49.1
	50	0.927	0.68	42.31
	200	1.579	-0.001	-0.06
Z13-6	100	1.603	-0.025	-1.58
	50	1.292	0.286	18.12
	200	2.085	0.312	13.02
Z13-7	100	2.156	0.241	10.05
	50	2.374	0.023	0.96
	200	1.815	-0.255	-16.35
Z13-8	100	0.799	0.761	48.78
	50	1.117	0.443	28.4
	200	1.477	0.22	12.96
Z13-9	100	1.422	0.275	16.21
	50	2.115	-0.418	-24.63
	200	1.636	0.061	3.59
Z13-10	100	1.802	-0.105	-6.19
	50	1.394	0.303	17.86
	200	1.8	-0.103	-6.07
Z13-11	100	1.758	-0.061	-3.59
	50	1.719	-0.022	-1.3

	200	1.267	0.43	25.34
Z13-12	100	1.822	-0.125	-7.37
	50	1.02	0.677	39.89
	200	0.204	0.017	-9.09
Z13-13	100	0.233	0.046	-24.48
	50	0.267	0.080	-42.66
	200	0.242	0.055	-29.37
Z13-14	100	0.240	0.052	-27.97
	50	0.236	0.048	-25.87

Table S3 Inhibition rate of **Z16**

**Comp.**	C(mg/L)	**Enzyme activity U**	**ΔU**	Inhibition rate %
	200	0.887	0.72	44.8
Z16-1	100	1.155	0.452	28.13
	50	1.143	0.464	28.87
	200	2.106	0.291	12.14
Z16-2	100	2.381	0.016	0.67
	50	2.179	0.218	9.09
	200	0.754	0.824	52.22
Z16-3	100	1.046	0.532	33.71
	50	0.887	0.691	43.79
	200	1.641	-0.241	-17.21
Z16-4	100	1.744	-0.344	-24.57
	50	1.947	-0.547	-39.07
	200	0.64	0.967	60.17
Z16-5	100	1.06	0.547	34.04
	50	1.013	0.594	36.96
	200	0.971	0.429	30.64
Z16-6	100	1.398	0.002	0.14
	50	2.599	-1.199	-85.64
	200	1.177	0.401	25.41
Z16-7	100	1.703	-0.125	-7.92
	50	1.334	0.244	15.46
	200	1.638	0.059	3.48
Z16-8	100	1.801	-0.104	-6.13
	50	1.777	-0.08	-4.71
	200	1.426	0.152	9.63
Z16-9	100	1.566	0.012	0.76
	50	1.57	0.008	0.51
	200	2.304	-0.726	-46.01
Z16-10	100	2.228	-0.65	-41.19
	50	2.337	-0.759	-48.1
Z16-11	200	1.454	0.124	7.86

	100	1.896	-0.318	-20.15
	50	2.576	-0.998	-63.24
	200	1.486	0.092	5.83
Z16-12	100	2.086	-0.508	-32.19
	50	2.1	-0.522	-33.08
	200	0.197	0.009	-4.90
Z16-13	100	0.191	0.004	-2.10
	50	0.231	0.043	-23.08
	200	0.212	0.025	-13.29
Z16-14	100	0.350	0.162	-27.27
	50	0.238	0.051	-86.71

References

[1]. Yanhong Dong, Potential green fungicide: 16-oxo-1-oxa-4-azoniacyclohexadecan-4-ium tetrafluoroborate. *Green Chemistry.* 2008,10, 990-994. DOI : 10.1039/B805797D.

Z12-1

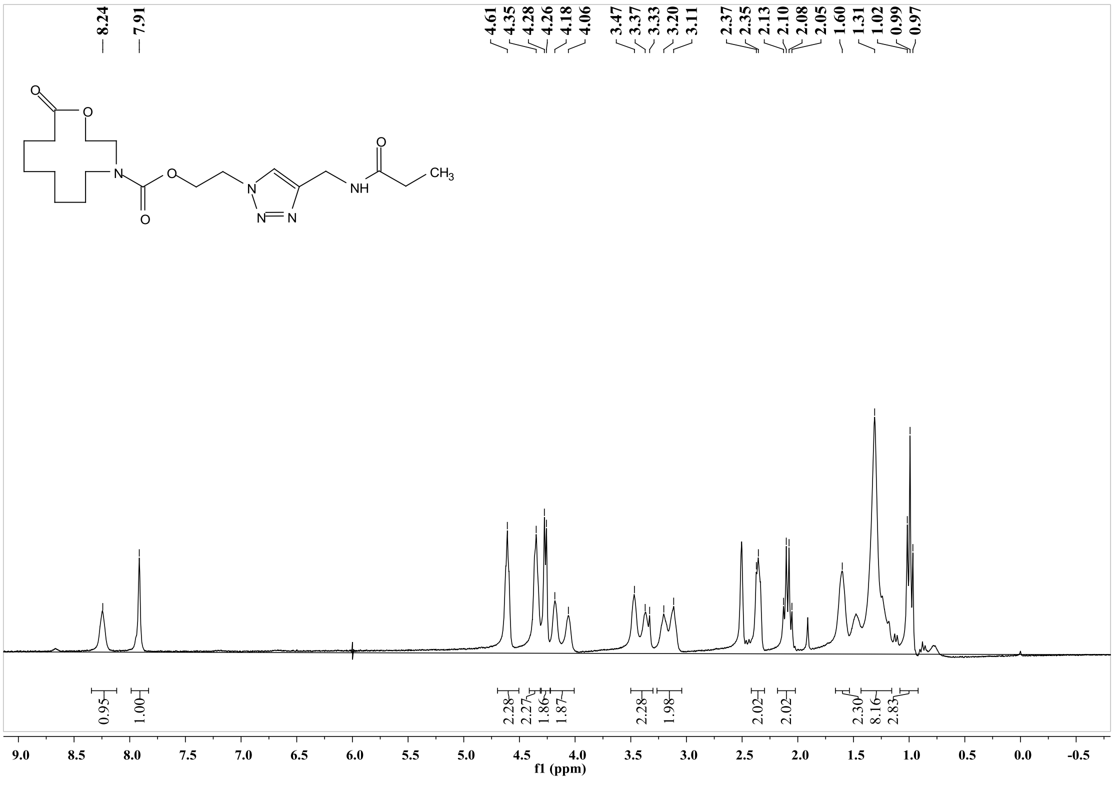

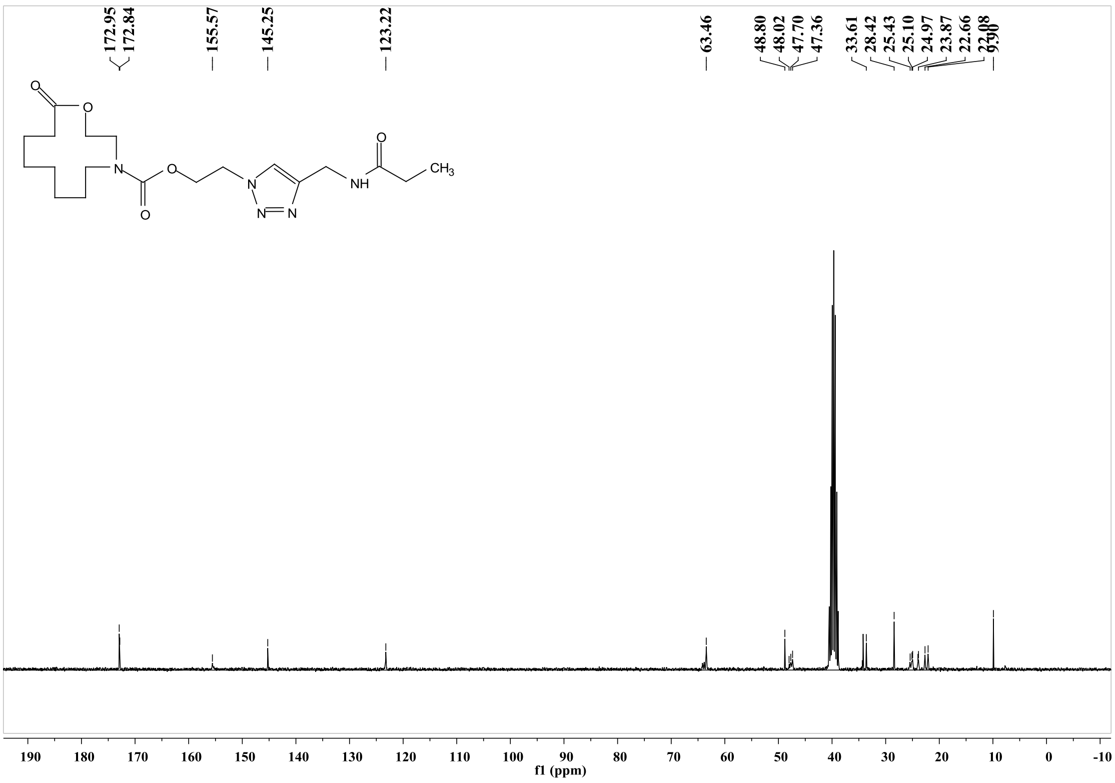

Z12-2

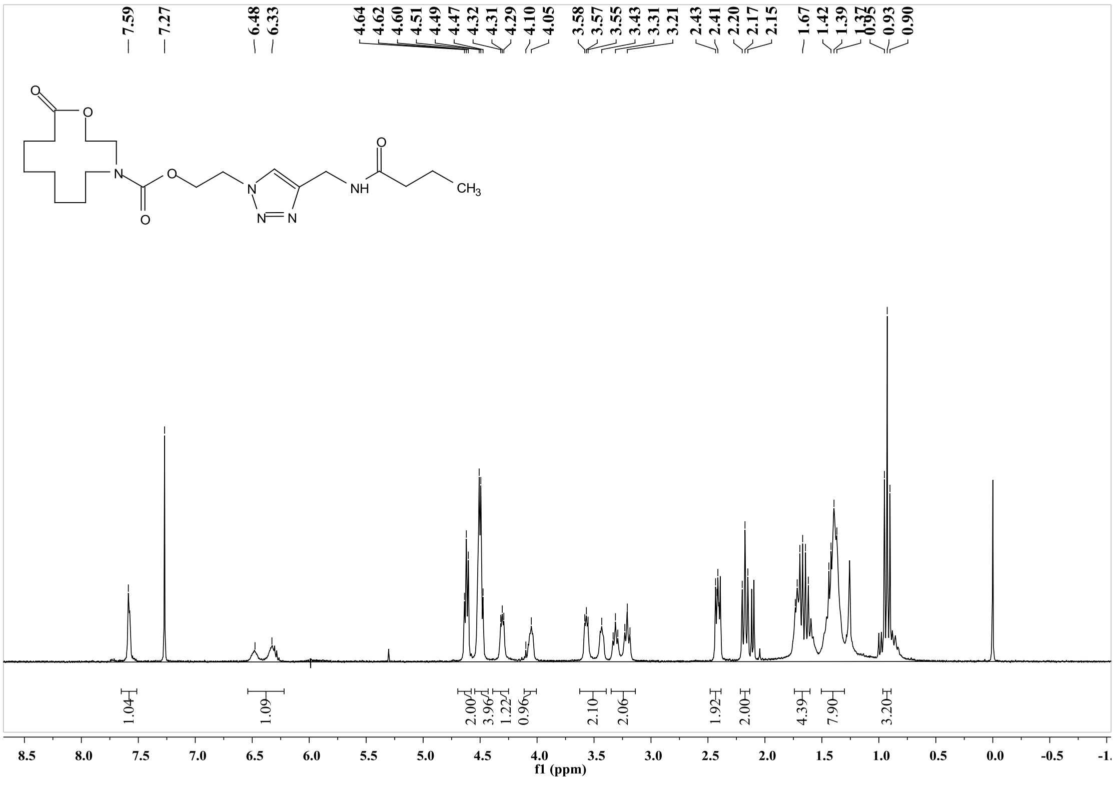

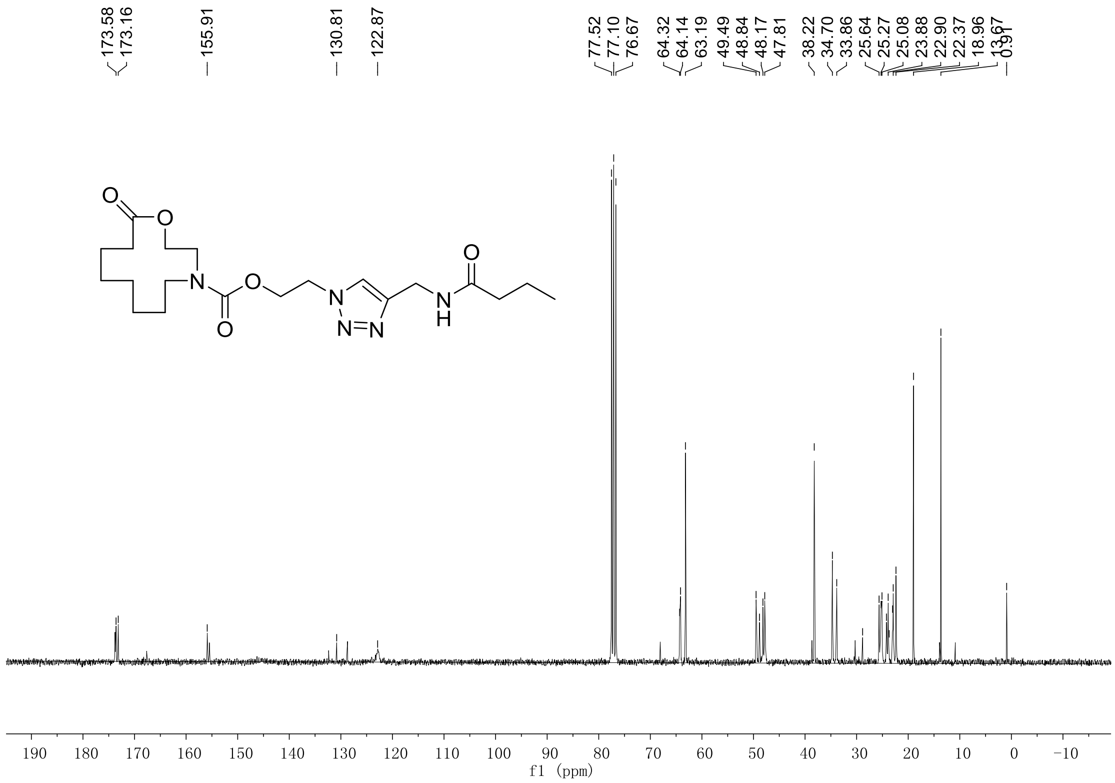
173.58
173.16
155.91
130.81
122.87
77.52
77.10
76.67
64.32
64.14
63.19
49.49
48.84
48.17
47.81
38.22
34.70
33.86
25.64
25.27
25.08
23.88
22.90
22.37
18.96
13.67
0.91
190 180 170 160 150 140 130 120 110 100 90 80 70 60 50 40 30 20 10 0 -10
f1 (ppm)

Z12-3

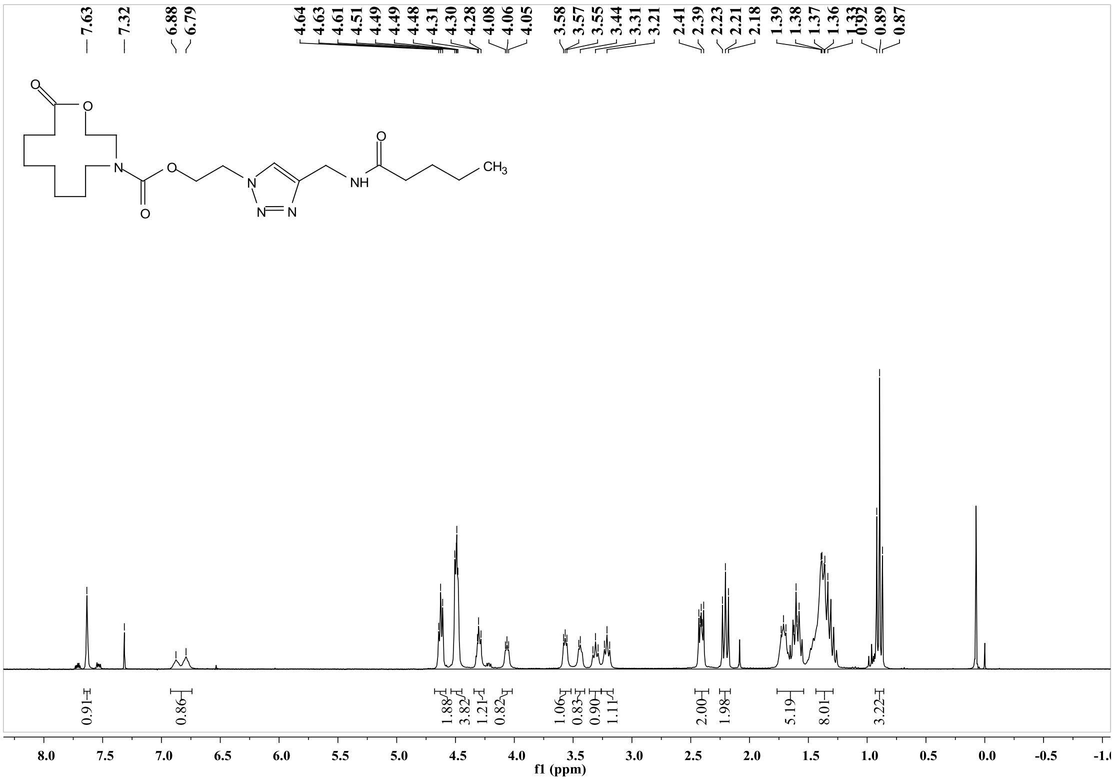
7.63
7.32
6.88
6.79
4.64
4.63
4.61
4.51
4.49
4.49
4.48
4.31
4.30
4.28
4.08
4.06
4.05
3.58
3.57
3.55
3.44
3.31
3.21
2.41
2.39
2.23
2.21
2.18
1.39
1.38
1.37
1.36
1.33
0.92
0.89
0.87
O
N
O
O
N
N
N
NH
O
CH3
0.91
0.86
1.88
3.82
1.21
0.82
1.06
0.83
0.90
1.11
2.00
1.98
5.19
8.01
3.22
f1 (ppm)

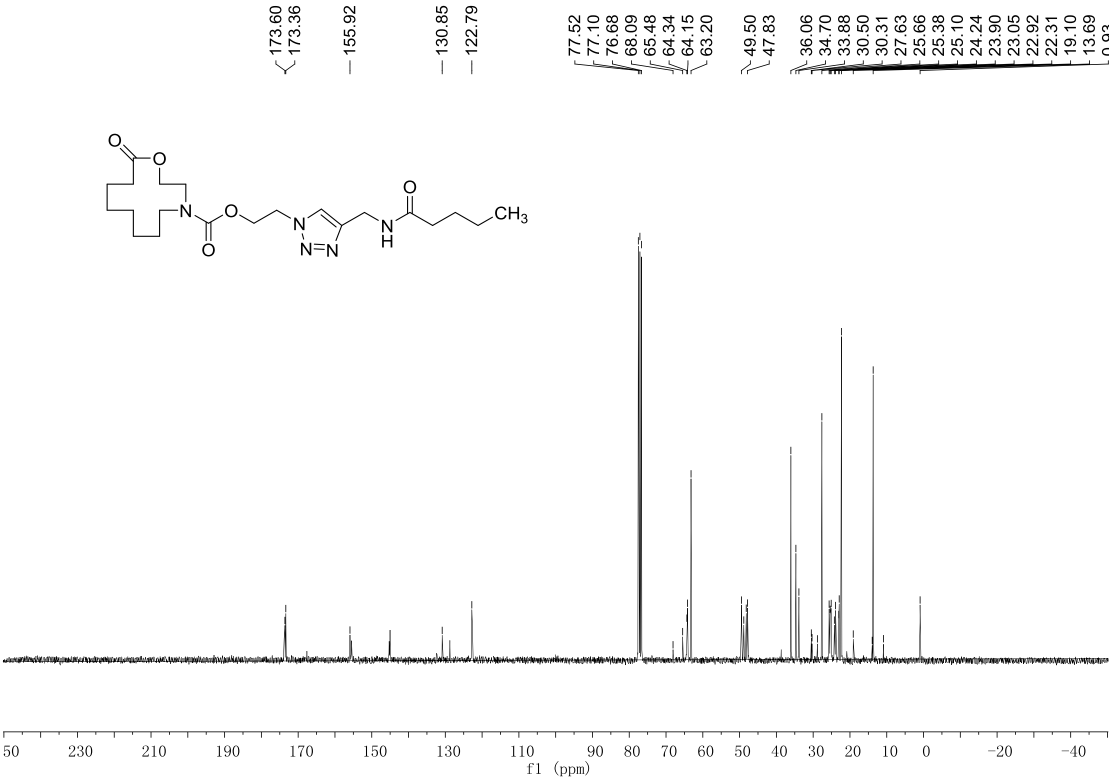
173.60
173.36
155.92
130.85
122.79
77.52
77.10
76.68
68.09
65.48
64.34
64.15
63.20
49.50
47.83
36.06
34.70
33.88
30.50
30.31
27.63
25.66
25.38
25.10
24.24
23.90
23.05
22.92
22.31
19.10
13.69
O
N
O
O
N
N
N
N
H
O
CH3
f1 (ppm)

12-4

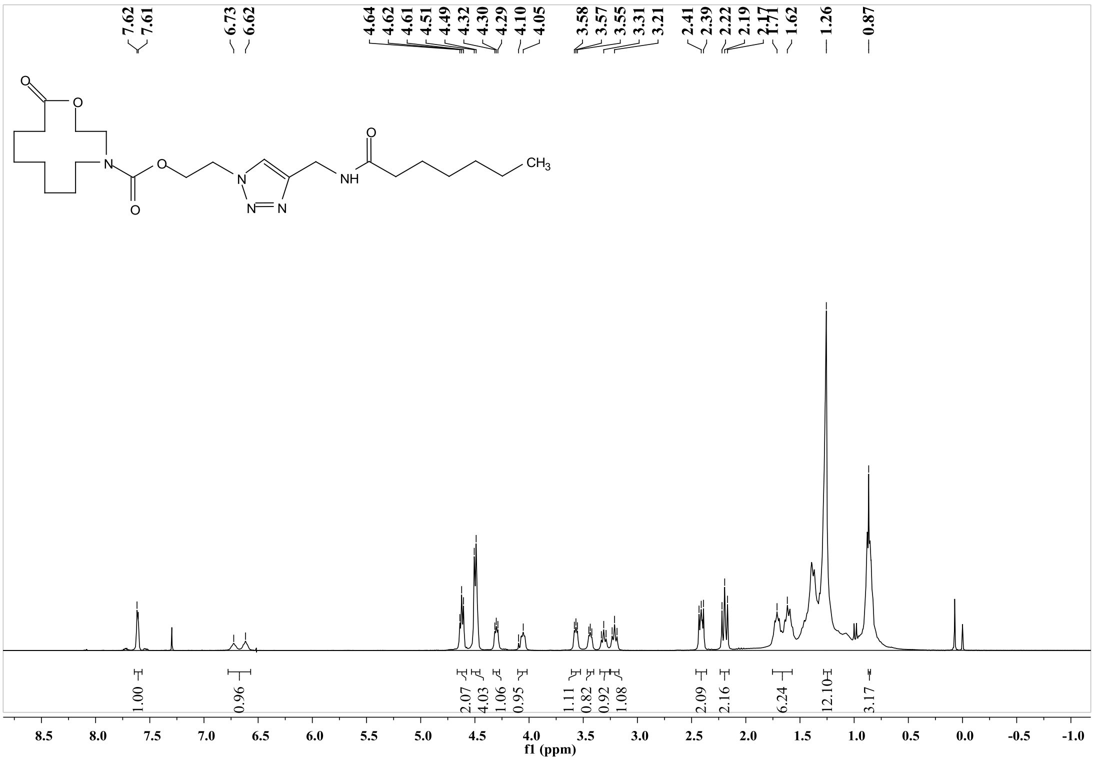
7.62
7.61
6.73
6.62
4.64
4.62
4.61
4.51
4.49
4.32
4.30
4.29
4.10
4.05
3.58
3.57
3.55
3.31
3.21
2.41
2.39
2.22
2.19
2.17
1.71
1.62
1.26
0.87
O
N
NH
N
N=N
CH3
1.00
0.96
2.07
4.03
1.06
0.95
1.11
0.82
0.92
1.08
2.09
2.16
6.24
12.10
3.17
f1 (ppm)

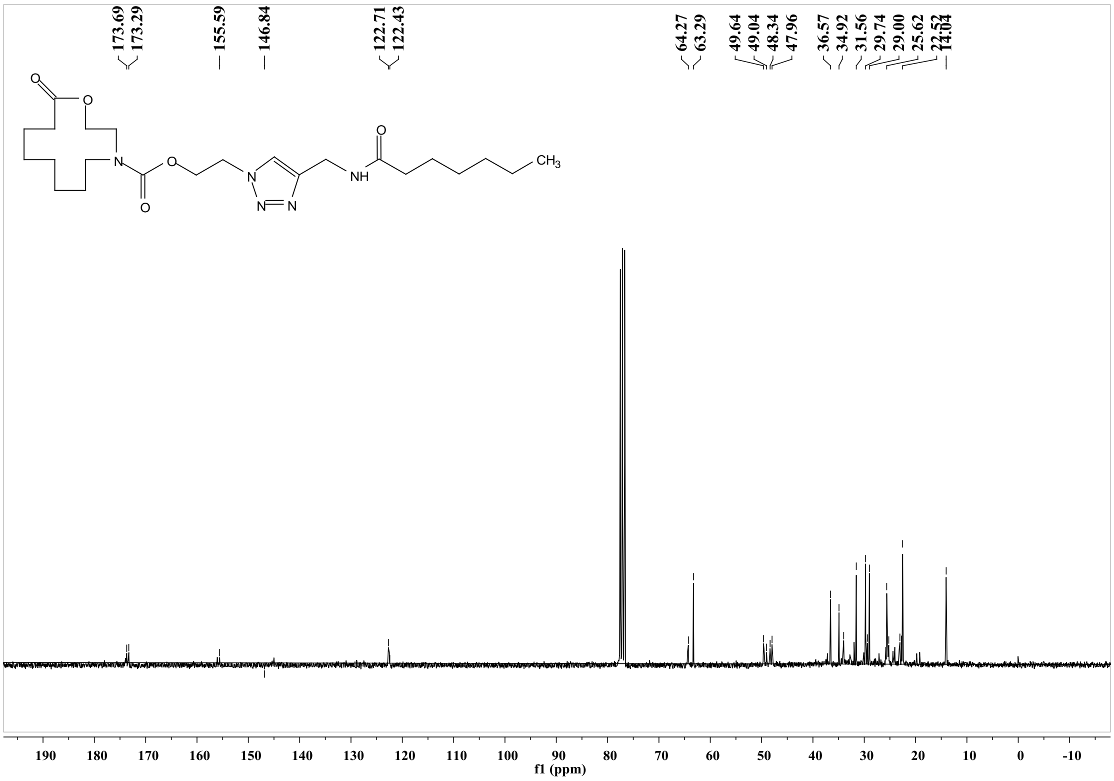
173.69
173.29
155.59
146.84
122.71
122.43
64.27
63.29
49.64
49.04
48.34
47.96
36.57
34.92
31.56
29.74
29.00
25.62
22.52
14.04
CH3
NH
f1 (ppm)

Z12-5

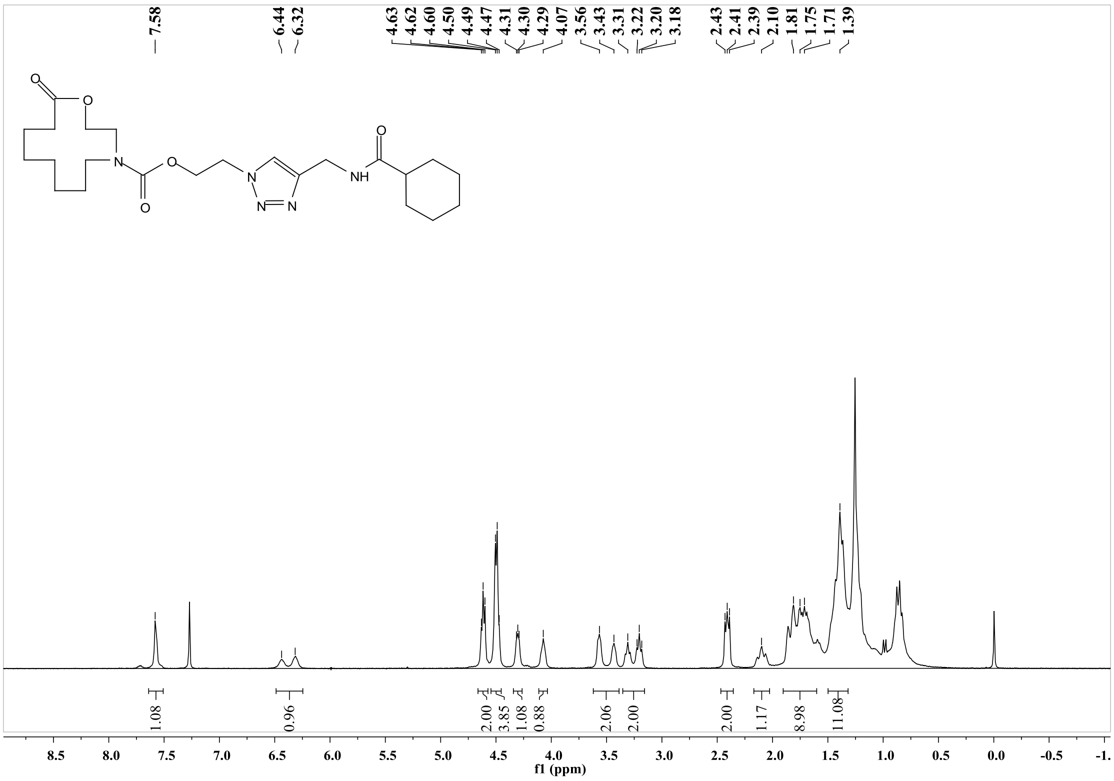
7.58
6.44
6.32
4.63
4.62
4.60
4.50
4.49
4.47
4.31
4.30
4.29
4.07
3.56
3.43
3.31
3.22
3.20
3.18
2.43
2.41
2.39
2.10
1.81
1.75
1.71
1.39
O
O
N
O
O
N
N
N
NH
O
1.08
0.96
2.00
3.85
1.08
0.88
2.06
2.00
2.00
1.17
8.98
11.08
8.5
8.0
7.5
7.0
6.5
6.0
5.5
5.0
4.5
4.0
3.5
3.0
2.5
2.0
1.5
1.0
0.5
0.0
-0.5
-1.
f1 (ppm)

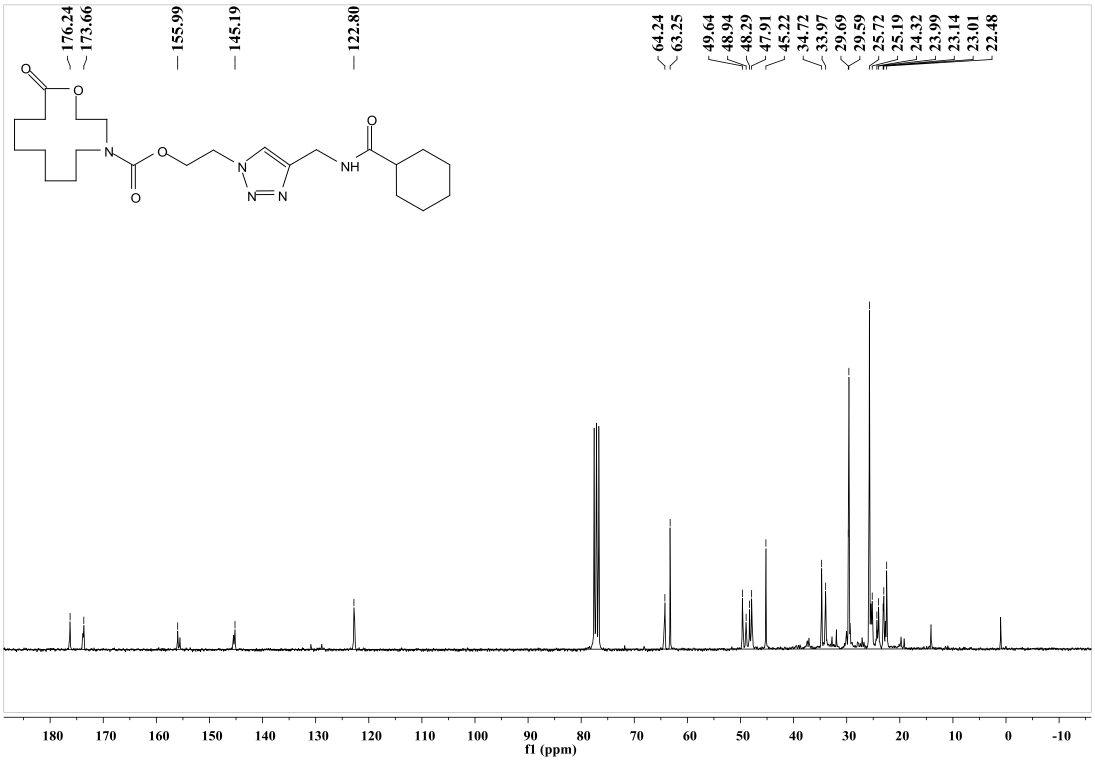
176.24
173.66
155.99
145.19
122.80
64.24
63.25
49.64
48.94
48.29
47.91
45.22
34.72
33.97
29.69
29.59
25.72
25.19
24.32
23.99
23.14
23.01
22.48
O
O
N
O
O
N
N
N
NH
O
180
170
160
150
140
130
120
110
100
90
80
70
60
50
40
30
20
10
0
-10
f1 (ppm)

Z12-6

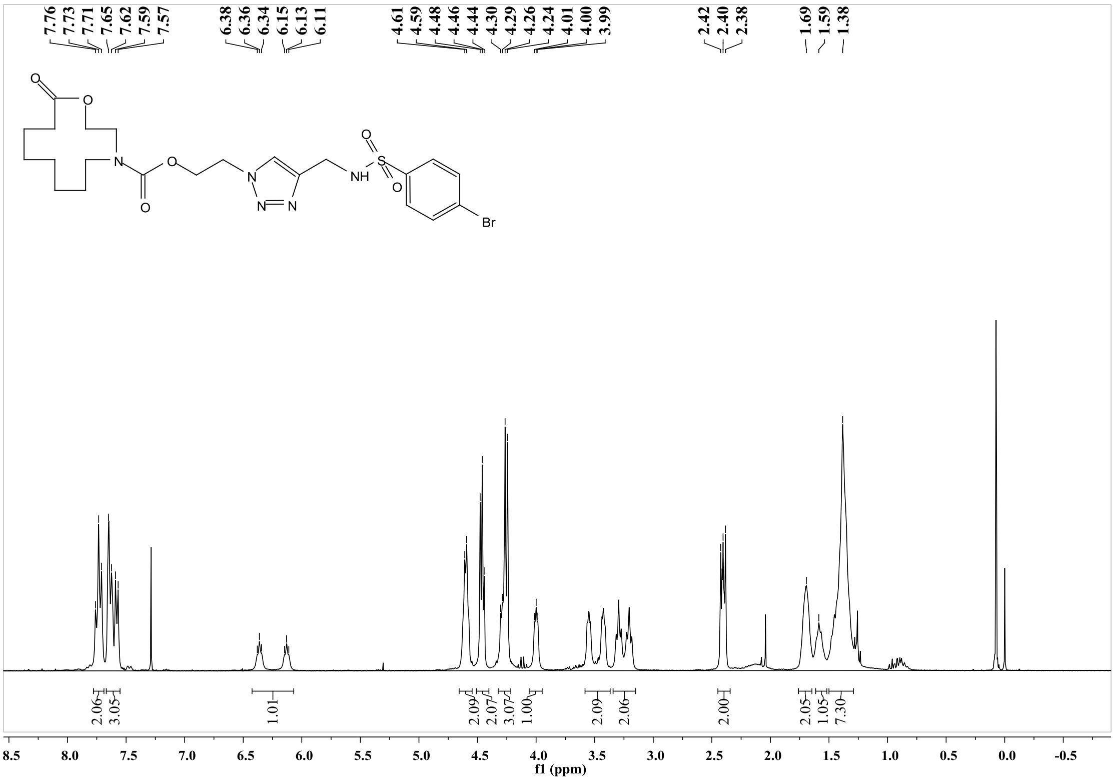
7.76
7.73
7.71
7.65
7.62
7.59
7.57
6.38
6.36
6.34
6.15
6.13
6.11
4.61
4.59
4.48
4.46
4.44
4.30
4.29
4.26
4.24
4.01
4.00
3.99
2.42
2.40
2.38
1.69
1.59
1.38
O
O
N
O
O
N
N
N
NH
S
O
O
Br
2.06
3.05
1.01
2.09
2.07
3.07
1.00
2.09
2.06
2.00
2.05
1.05
7.30
f1 (ppm)

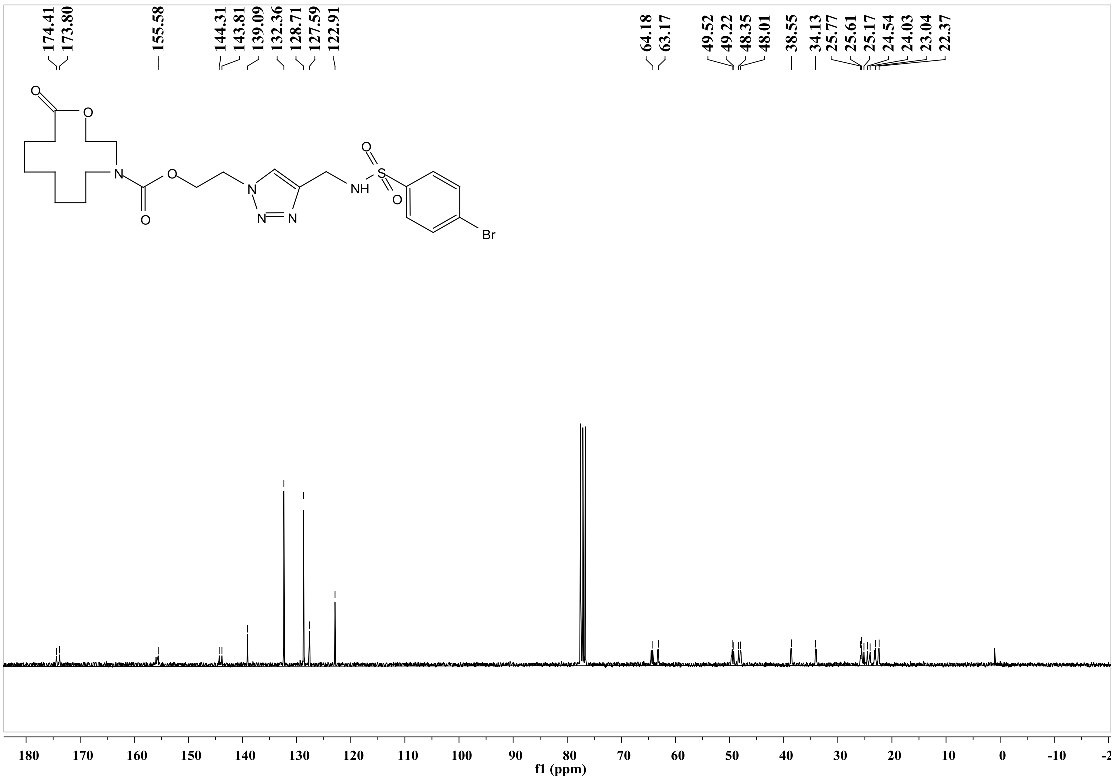
174.41
173.80
155.58
144.31
143.81
139.09
132.36
128.71
127.59
122.91
64.18
63.17
49.52
49.22
48.35
48.01
38.55
34.13
25.77
25.61
25.17
24.54
24.03
23.04
22.37
O
O
N
O
O
N
N
N
NH
S
O
O
Br
f1 (ppm)

Z12-7

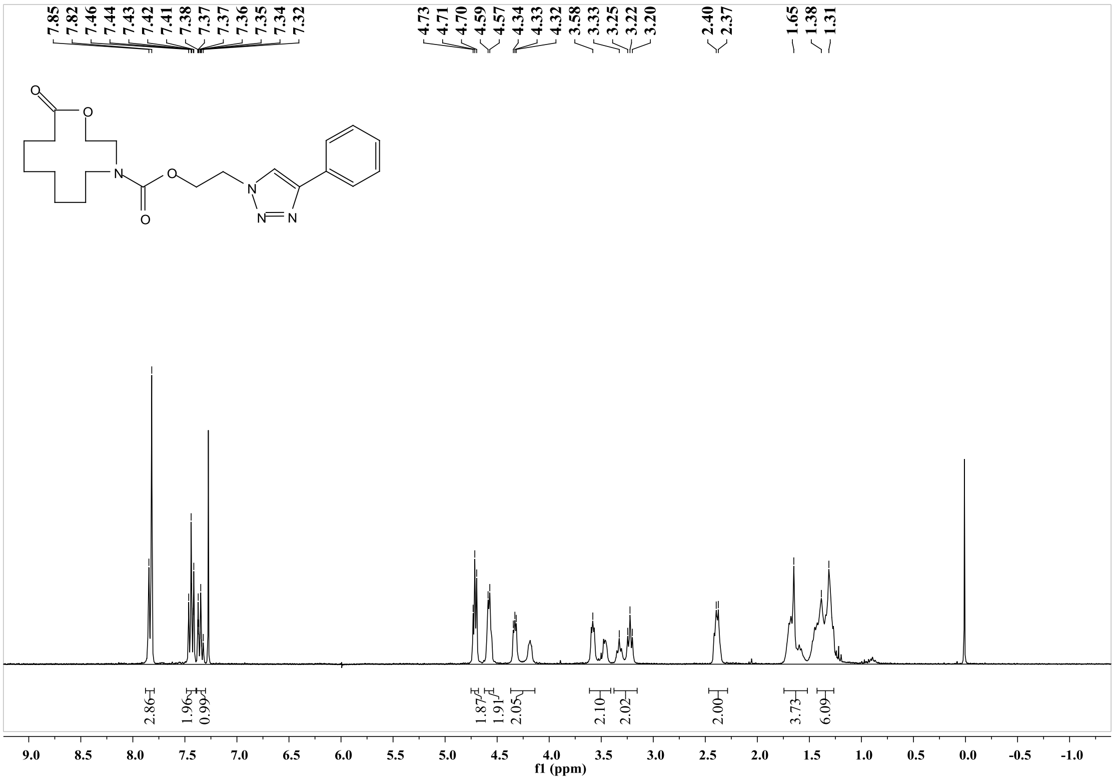
7.85
7.82
7.46
7.44
7.43
7.42
7.41
7.38
7.37
7.37
7.36
7.35
7.34
7.32
4.73
4.71
4.70
4.59
4.57
4.34
4.33
4.32
3.58
3.33
3.25
3.22
3.20
2.40
2.37
1.65
1.38
1.31
O
O
N
O
O
N
N=N
2.86
1.96
0.99
1.87
1.91
2.05
2.10
2.02
2.00
3.73
6.09
9.0 8.5 8.0 7.5 7.0 6.5 6.0 5.5 5.0 4.5 4.0 3.5 3.0 2.5 2.0 1.5 1.0 0.5 0.0 -0.5 -1.0
f1 (ppm)

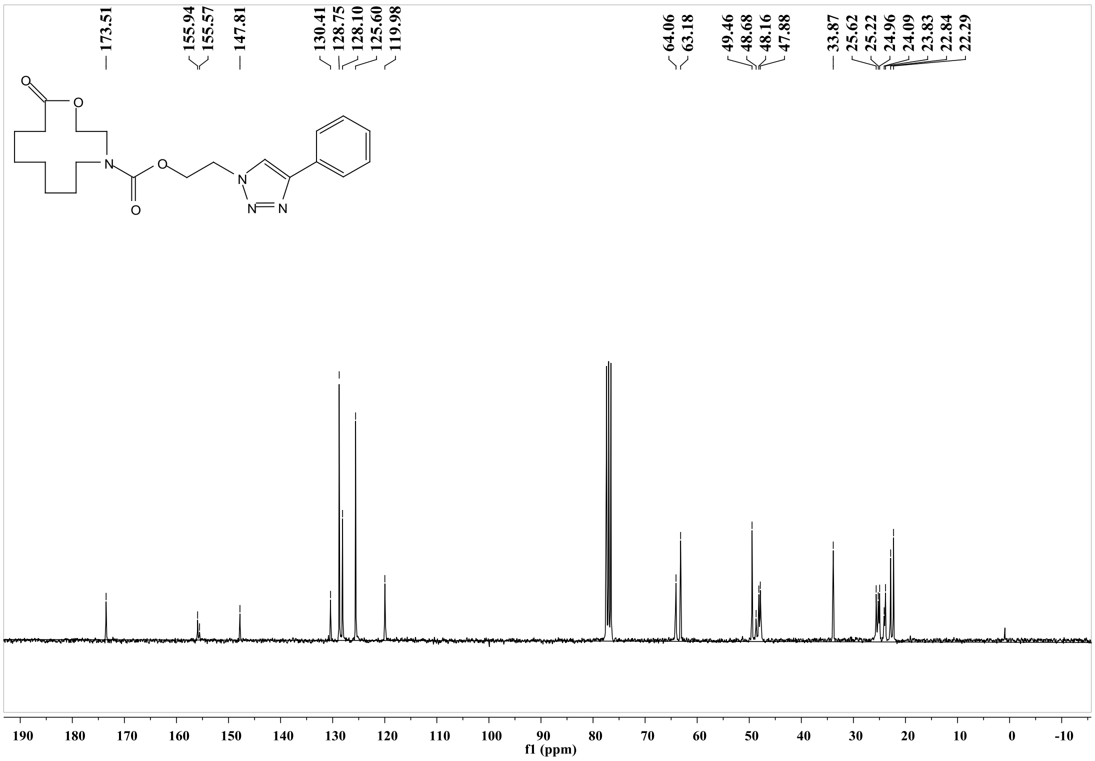
173.51
155.94
155.57
147.81
130.41
128.75
128.10
125.60
119.98
64.06
63.18
49.46
48.68
48.16
47.88
33.87
25.62
25.22
24.96
24.09
23.83
22.84
22.29
O
O
N
O
O
N
N=N
190 180 170 160 150 140 130 120 110 100 90 80 70 60 50 40 30 20 10 0 -10
f1 (ppm)

Z12-8

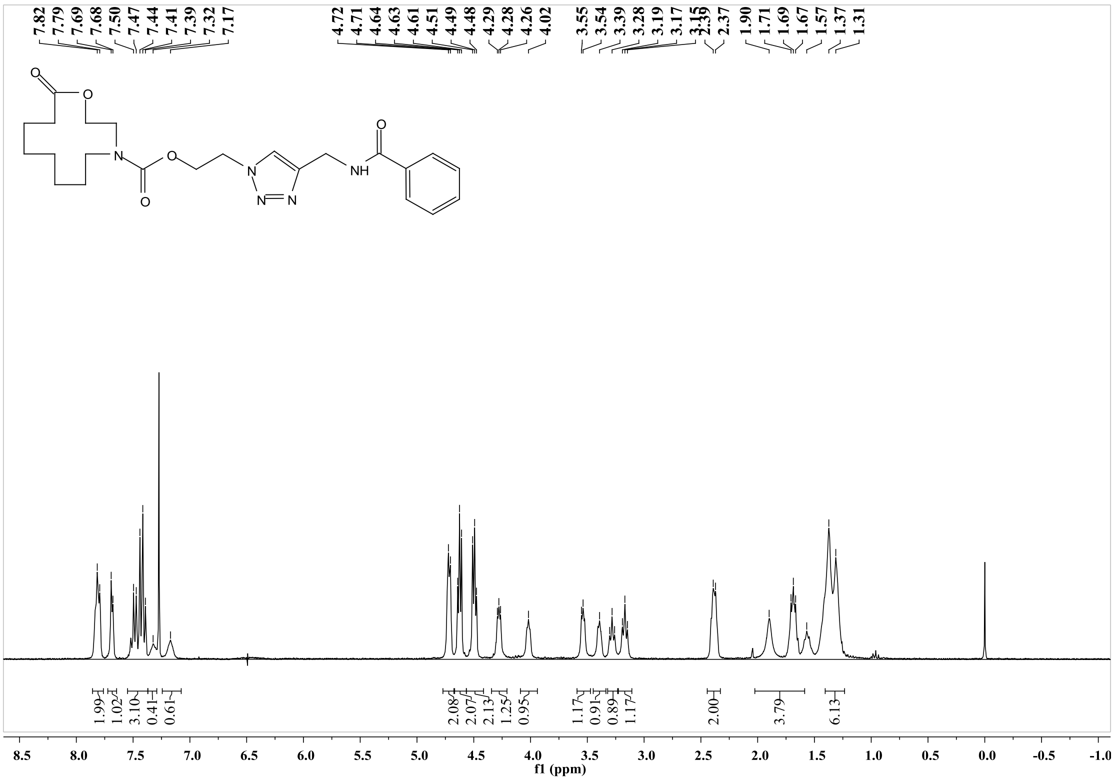

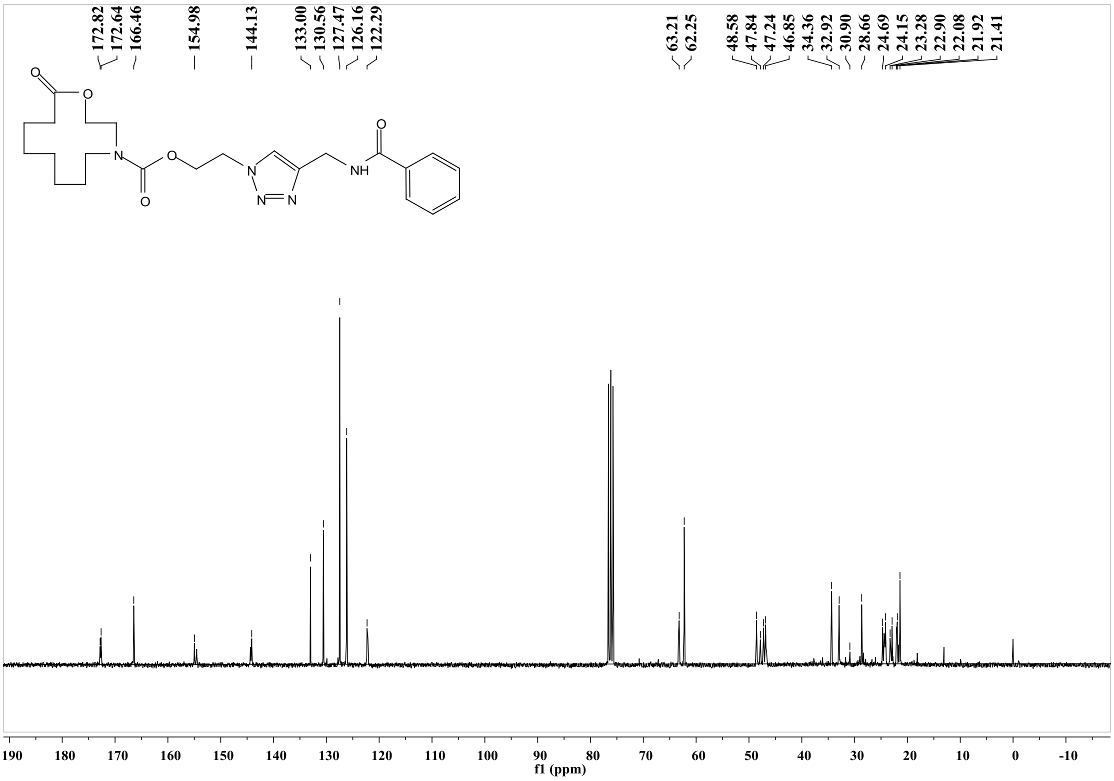

Z12-9

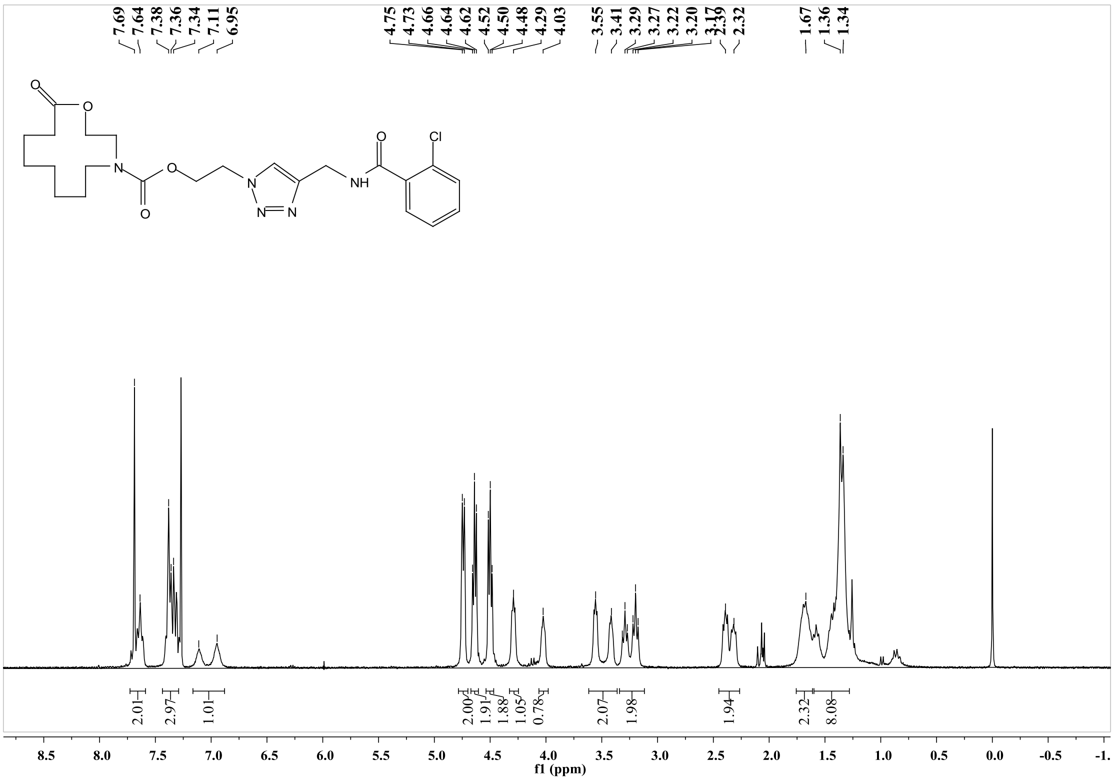
7.69
7.64
7.38
7.36
7.34
7.11
6.95
4.75
4.73
4.66
4.64
4.62
4.52
4.50
4.48
4.29
4.03
3.55
3.41
3.29
3.27
3.22
3.20
3.17
2.39
2.32
1.67
1.36
1.34
2.01
2.97
1.01
2.00
1.91
1.88
1.05
0.78
2.07
1.98
1.94
2.32
8.08
f1 (ppm)

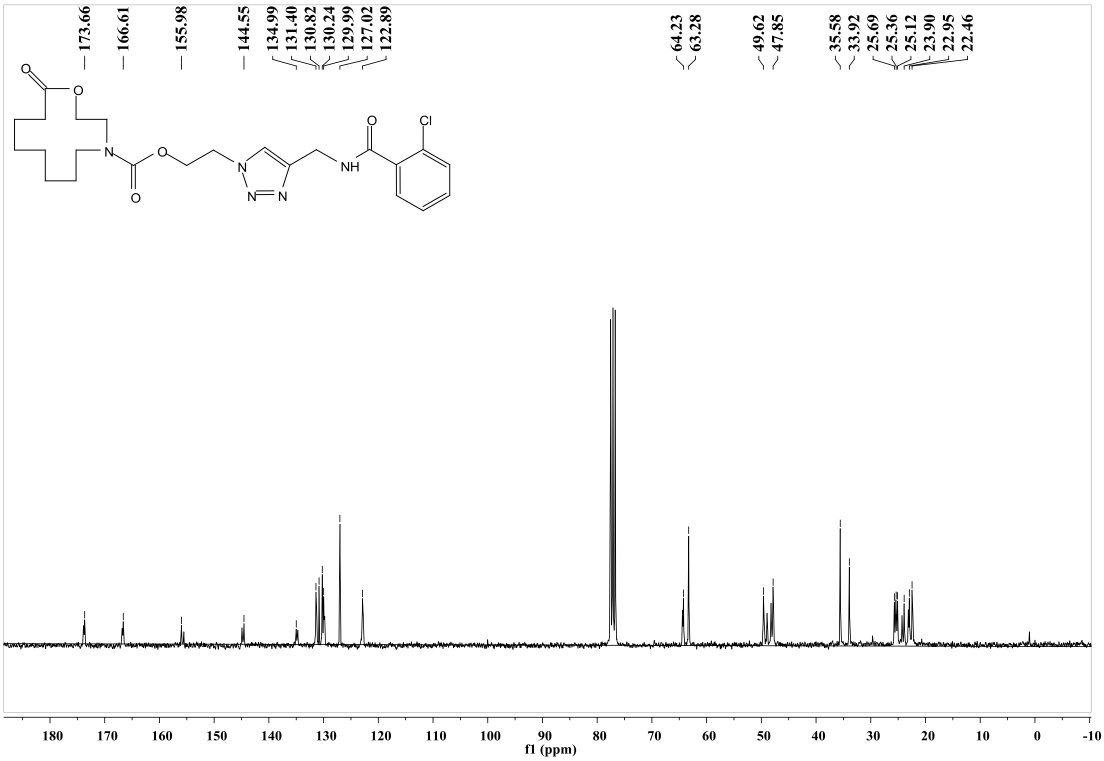
173.66
166.61
155.98
144.55
134.99
131.40
130.82
130.24
129.99
127.02
122.89
64.23
63.28
49.62
47.85
35.58
33.92
25.69
25.36
25.12
23.90
22.95
22.46
f1 (ppm)

Z12-10

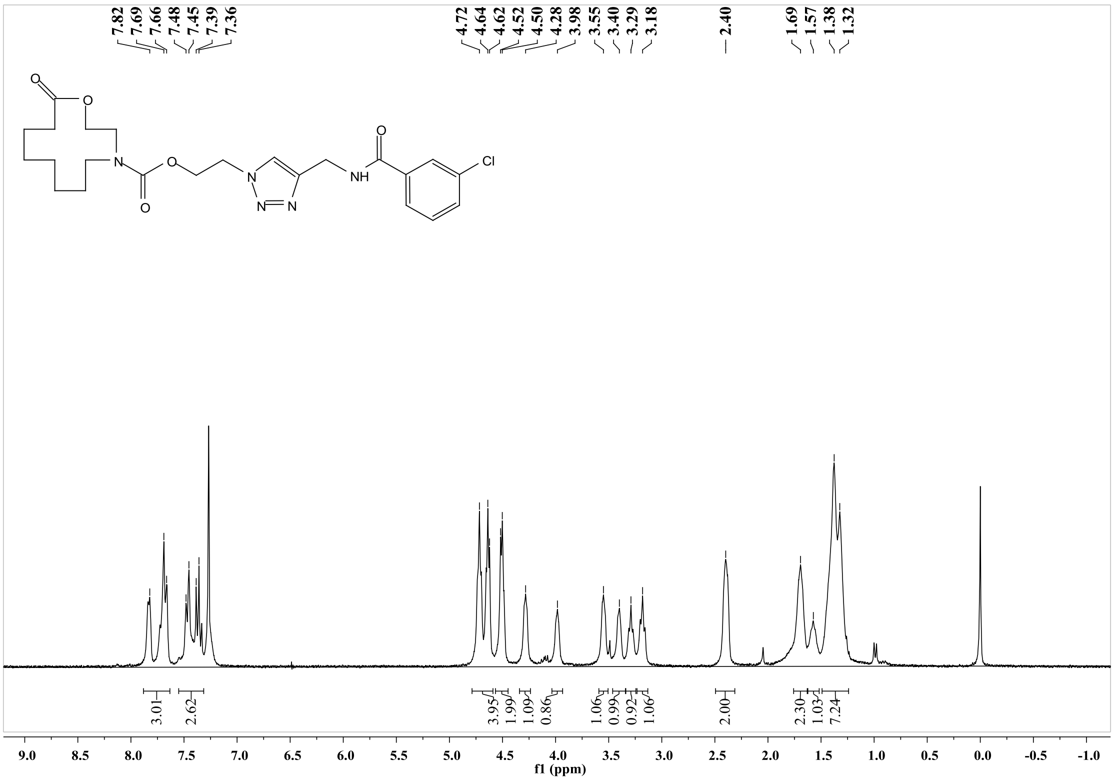

7.82
7.69
7.66
7.48
7.45
7.39
7.36
4.72
4.64
4.62
4.52
4.50
4.28
3.98
3.55
3.40
3.29
3.18
2.40
1.69
1.57
1.38
1.32
O
O
N
O
O
N
N
N
NH
O
Cl
3.01
2.62
3.95
1.99
1.09
0.86
1.06
0.99
0.92
1.06
2.00
2.30
1.03
7.24
9.0
8.5
8.0
7.5
7.0
6.5
6.0
5.5
5.0
4.5
4.0
3.5
3.0
2.5
2.0
1.5
1.0
0.5
0.0
-0.5
-1.0
f1 (ppm)

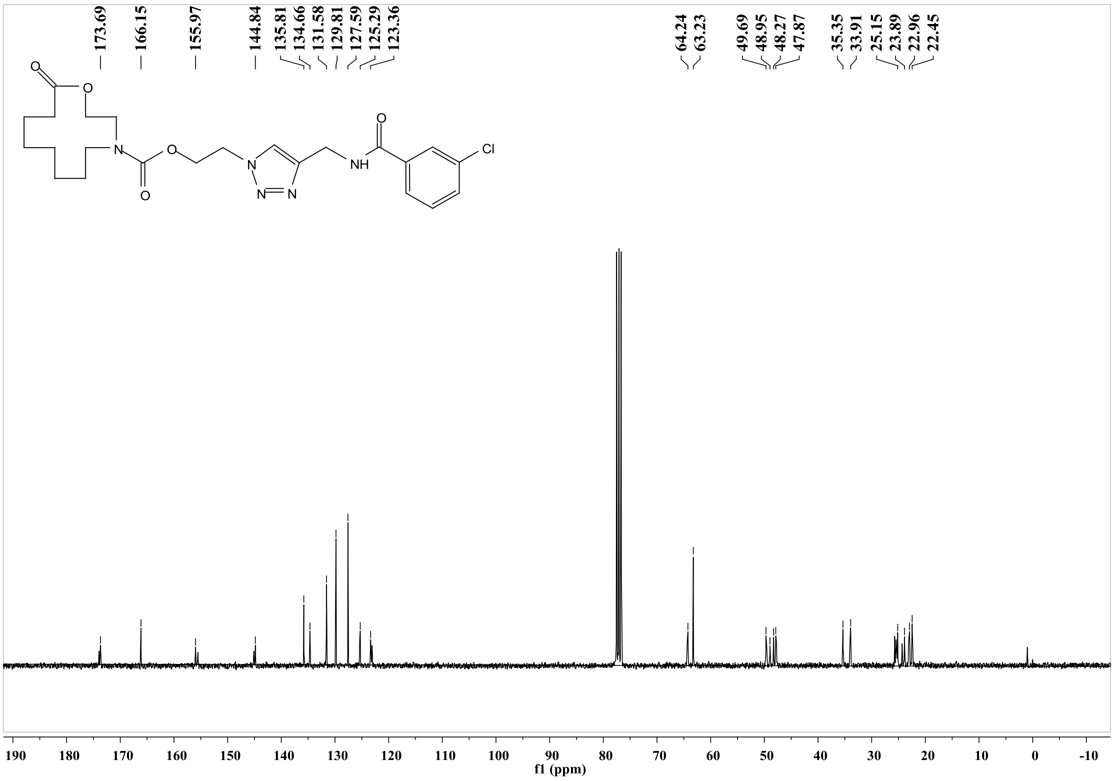

173.69
166.15
155.97
144.84
135.81
134.66
131.58
129.81
127.59
125.29
123.36
64.24
63.23
49.69
48.95
48.27
47.87
35.35
33.91
25.15
23.89
22.96
22.45
O
O
N
O
O
N
N
N
NH
O
Cl
190
180
170
160
150
140
130
120
110
100
90
80
70
60
50
40
30
20
10
0
-10
f1 (ppm)

Z12-11

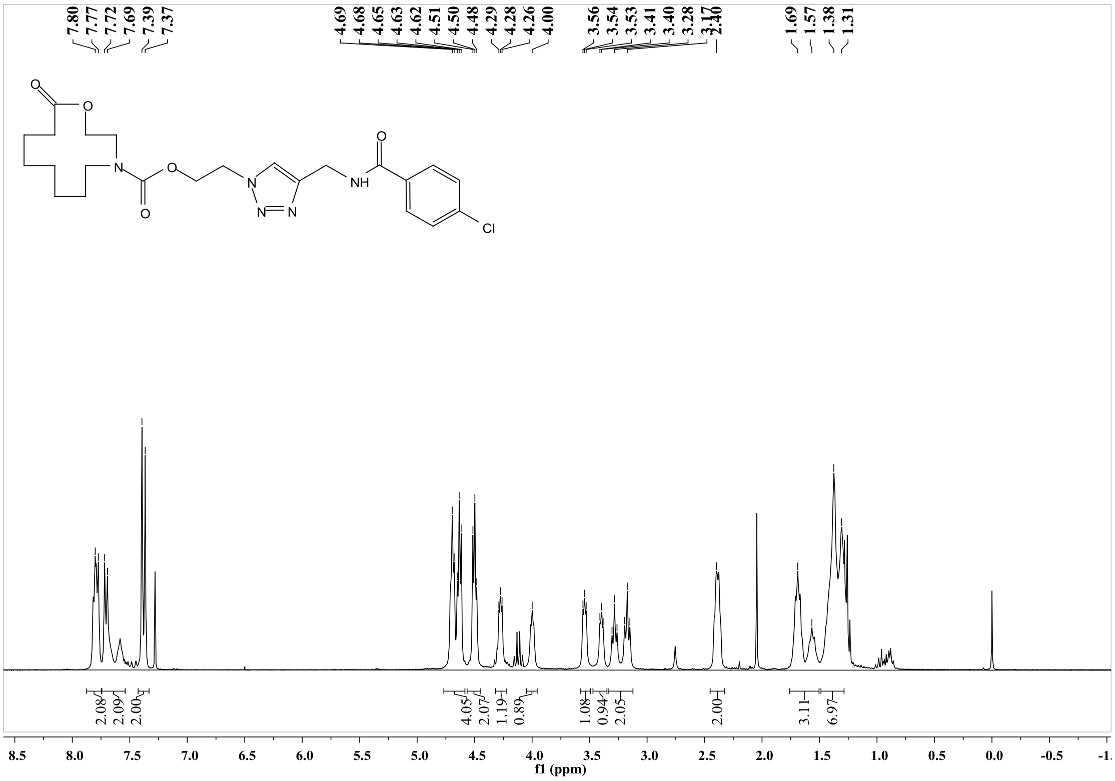

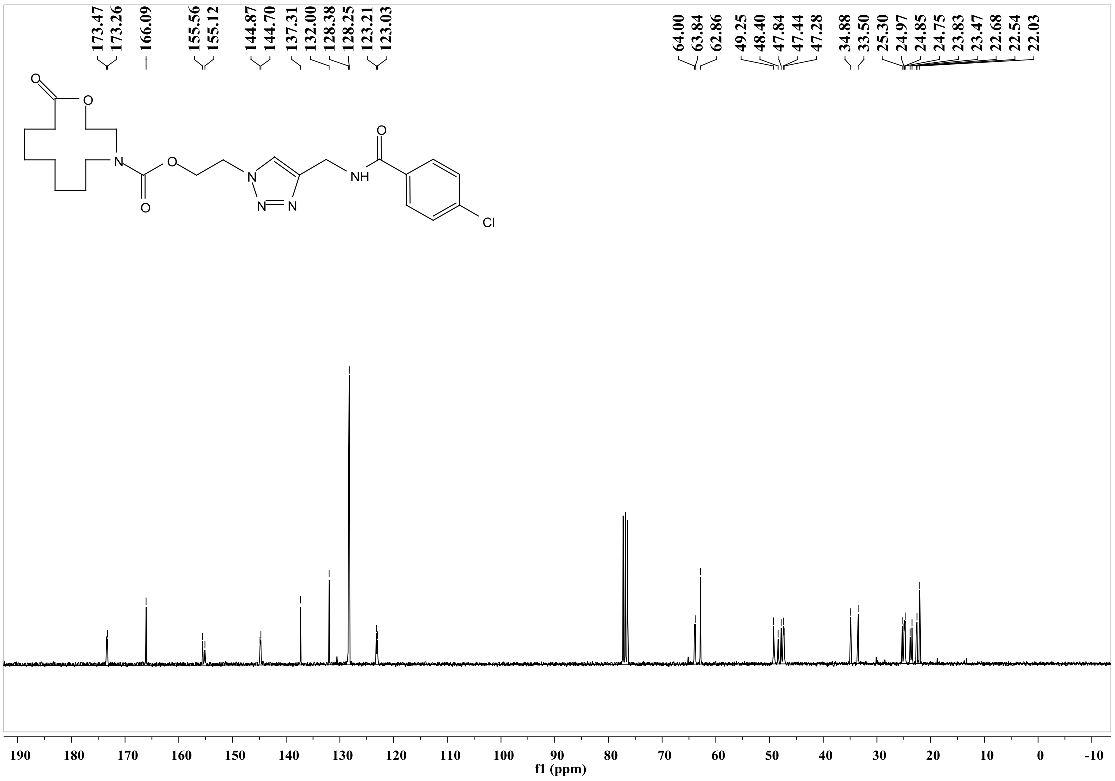

Z12-12

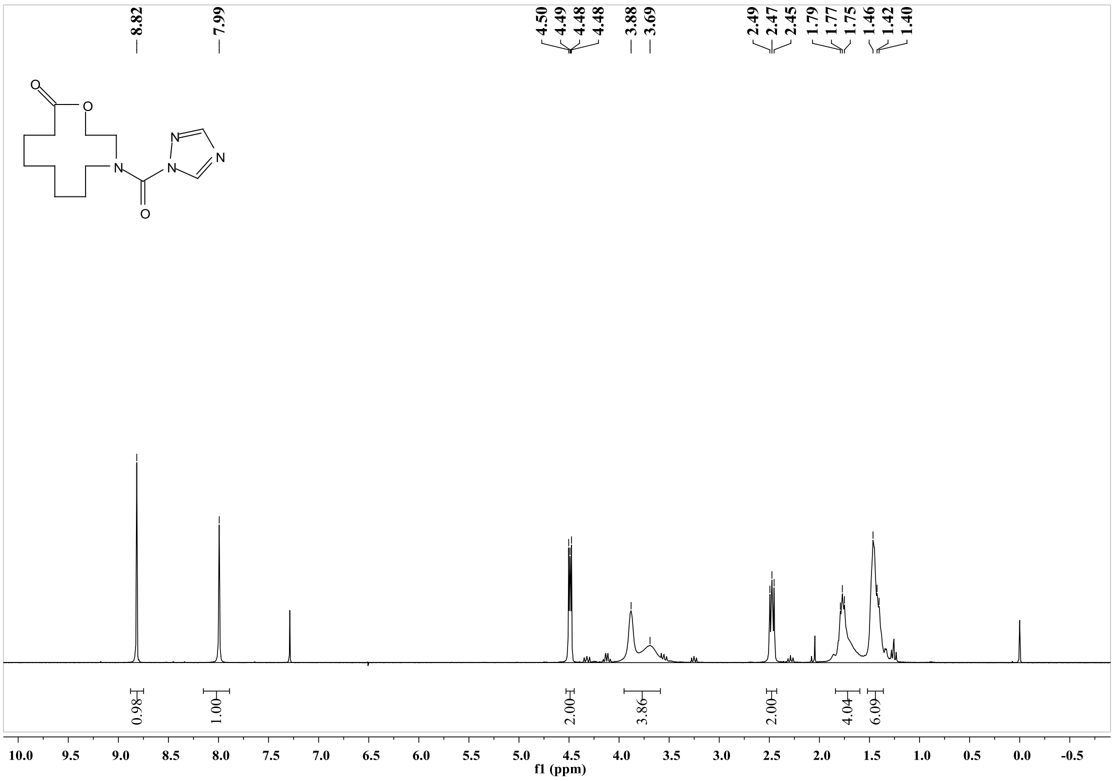
8.82
7.99
4.50
4.49
4.48
4.48
3.88
3.69
2.49
2.47
2.45
1.79
1.77
1.75
1.46
1.42
1.40
0.98
1.00
2.00
3.86
2.00
4.04
6.09
10.0 9.5 9.0 8.5 8.0 7.5 7.0 6.5 6.0 5.5 5.0 4.5 4.0 3.5 3.0 2.5 2.0 1.5 1.0 0.5 0.0 -0.5
f1 (ppm)

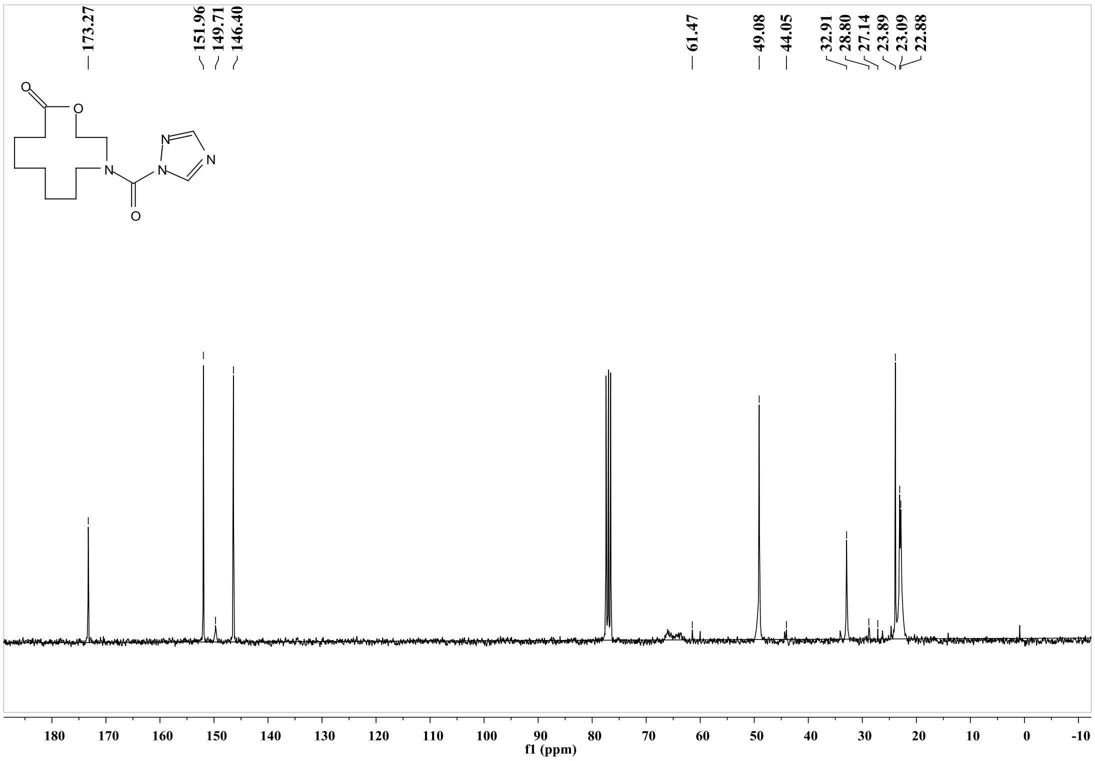
173.27
151.96
149.71
146.40
61.47
49.08
44.05
32.91
28.80
27.14
23.89
23.09
22.88
180 170 160 150 140 130 120 110 100 90 80 70 60 50 40 30 20 10 0 -10
f1 (ppm)

Z12-13

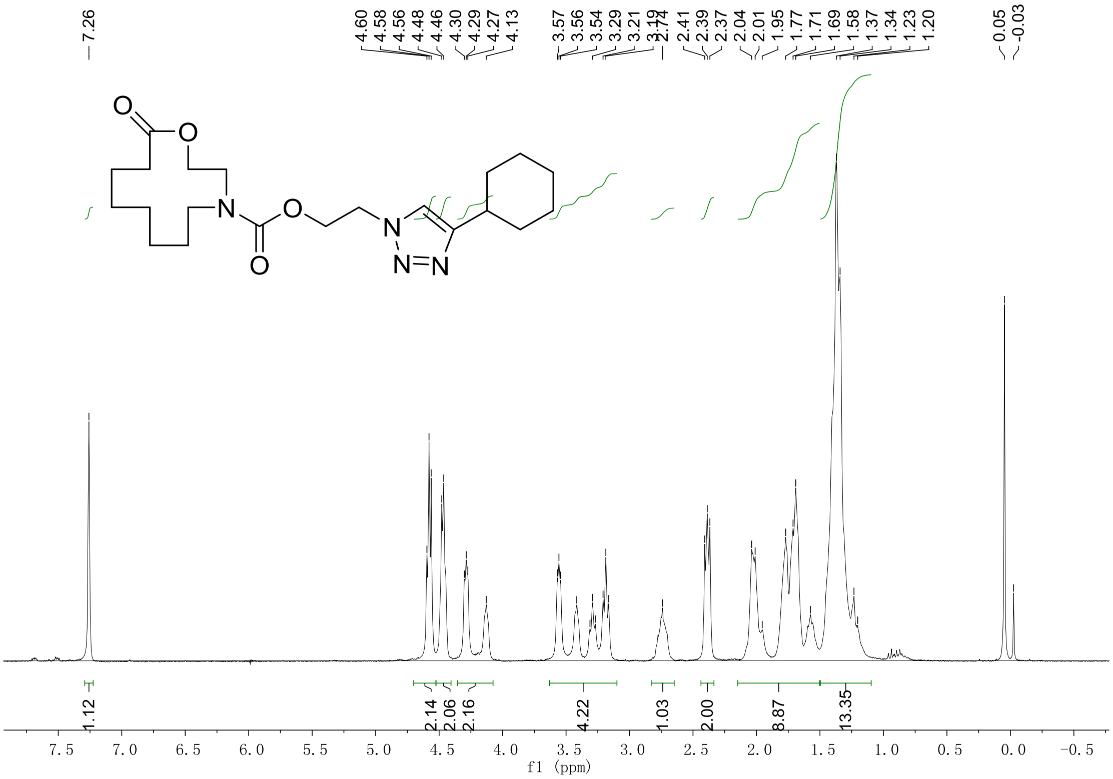
7.26
4.60
4.58
4.56
4.48
4.46
4.30
4.29
4.27
4.13
3.57
3.56
3.54
3.29
3.21
3.19
2.74
2.41
2.39
2.37
2.04
2.01
1.95
1.77
1.71
1.69
1.58
1.37
1.34
1.23
1.20
0.05
-0.03
1.12
2.14
2.06
2.16
4.22
1.03
2.00
8.87
13.35
f1 (ppm)

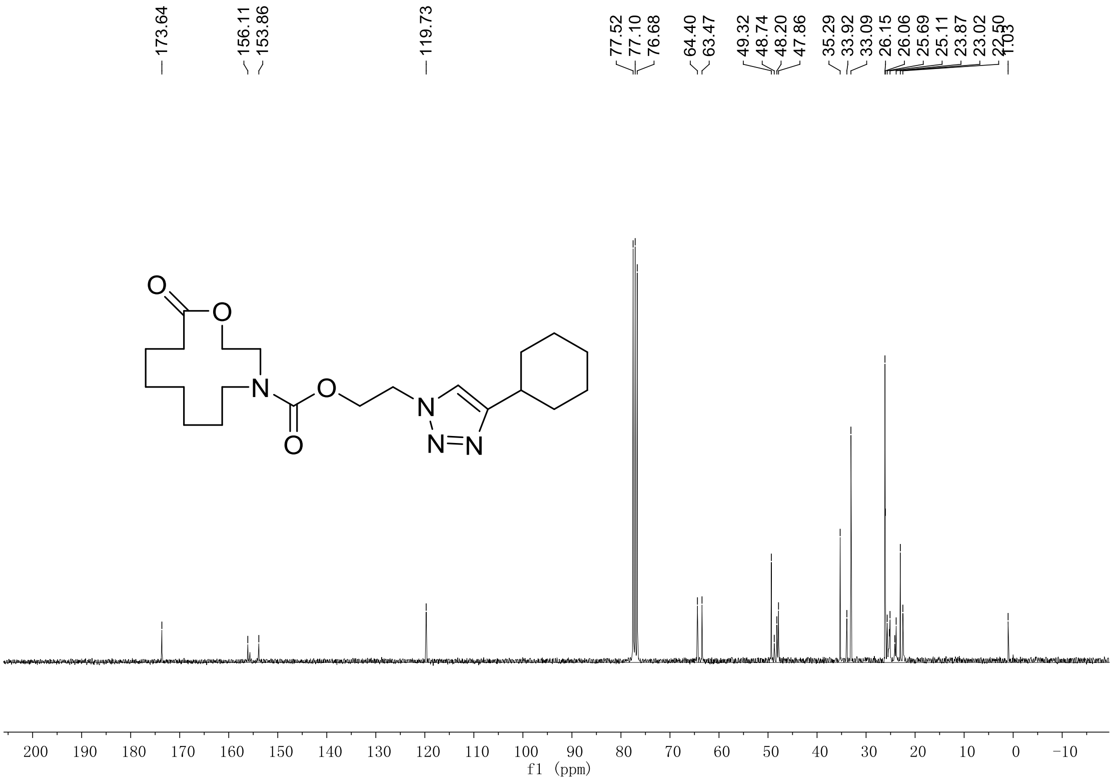
173.64
156.11
153.86
119.73
77.52
77.10
76.68
64.40
63.47
49.32
48.74
48.20
47.86
35.29
33.92
33.09
26.15
26.06
25.69
25.11
23.87
23.02
22.50
1.03
f1 (ppm)

Z12-14

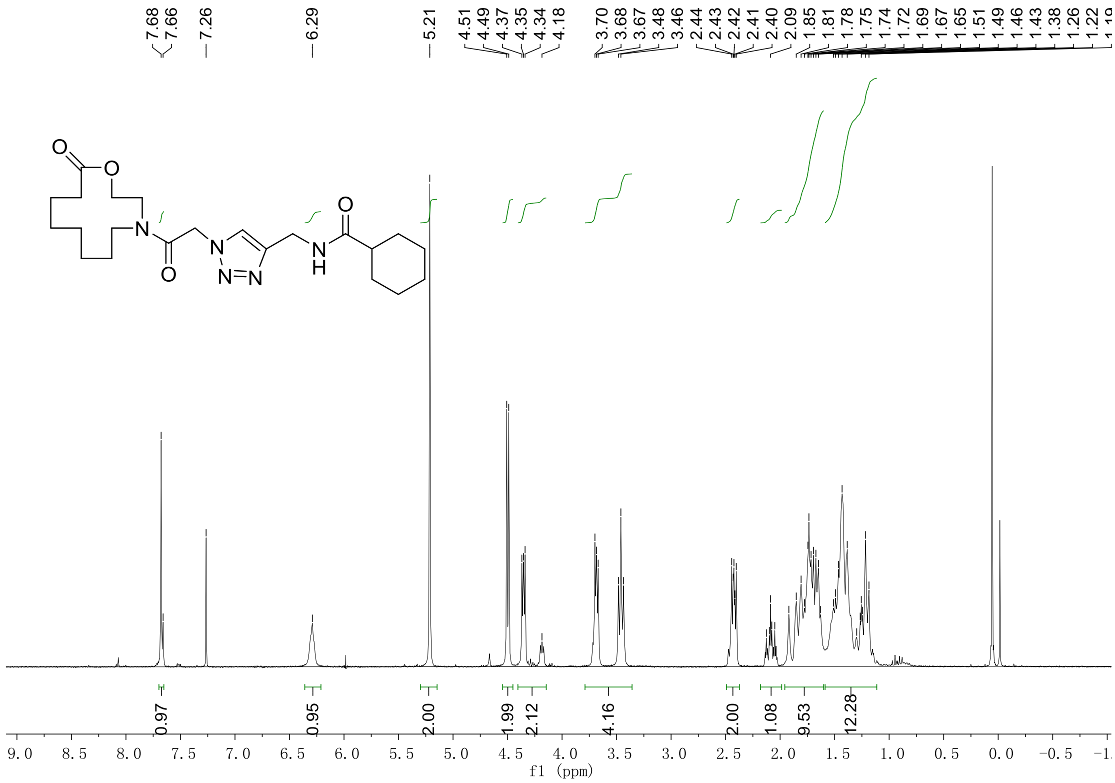

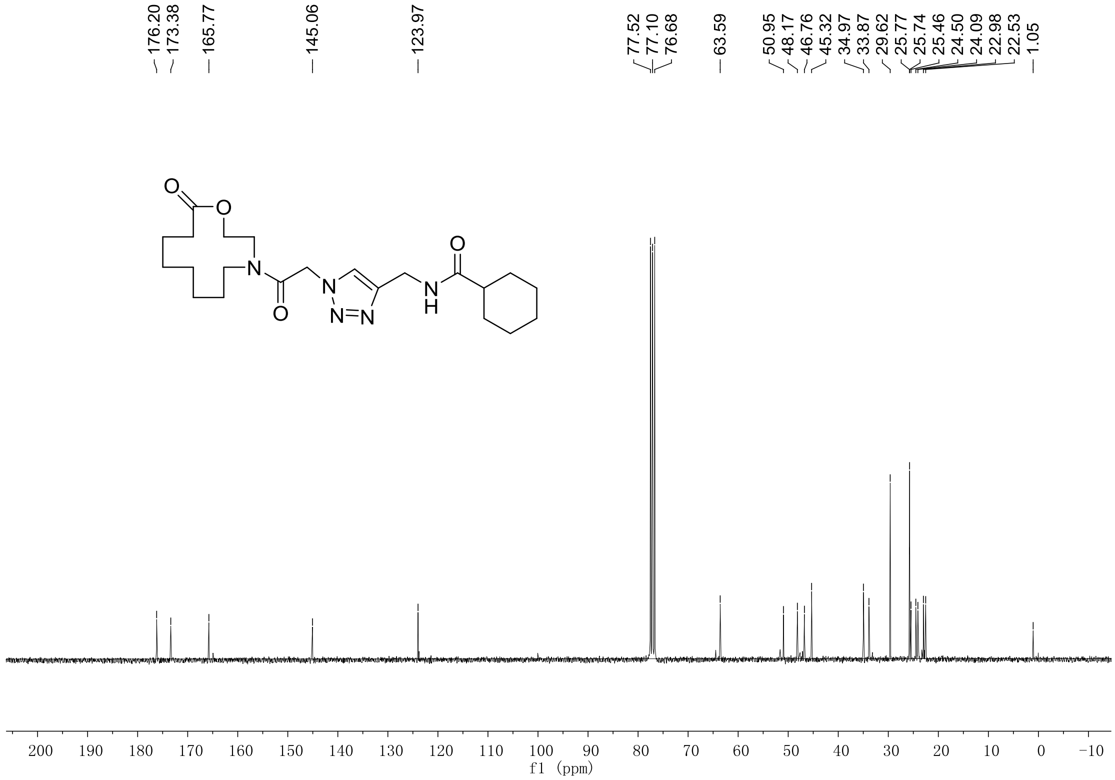

Z13-1

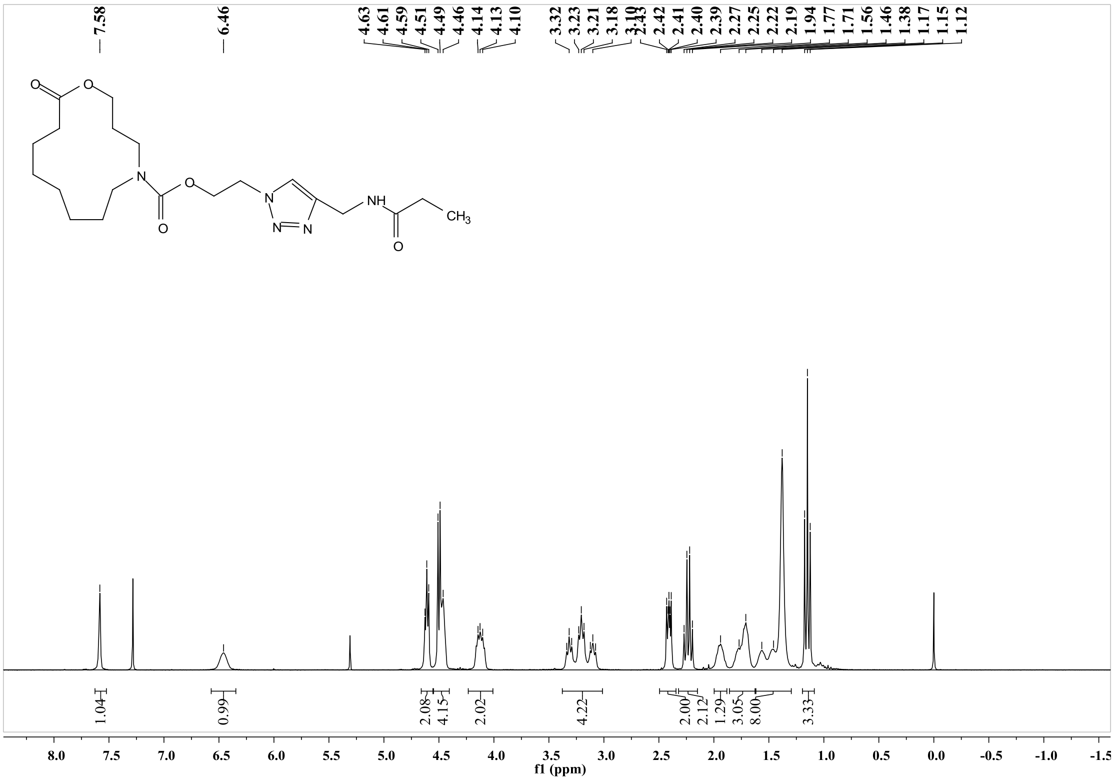
7.58
6.46
4.63
4.61
4.59
4.51
4.49
4.46
4.14
4.13
4.10
3.32
3.23
3.21
3.18
3.10
2.43
2.42
2.41
2.40
2.39
2.27
2.25
2.22
2.19
1.94
1.77
1.71
1.56
1.46
1.38
1.17
1.15
1.12
O
O
N
O
O
N
N
N
NH
O
CH_3
1.04
0.99
2.08
4.15
2.02
4.22
2.00
2.12
1.29
3.05
8.00
3.33
8.0
7.5
7.0
6.5
6.0
5.5
5.0
4.5
4.0
3.5
3.0
2.5
2.0
1.5
1.0
0.5
0.0
-0.5
-1.0
-1.5
f1 (ppm)

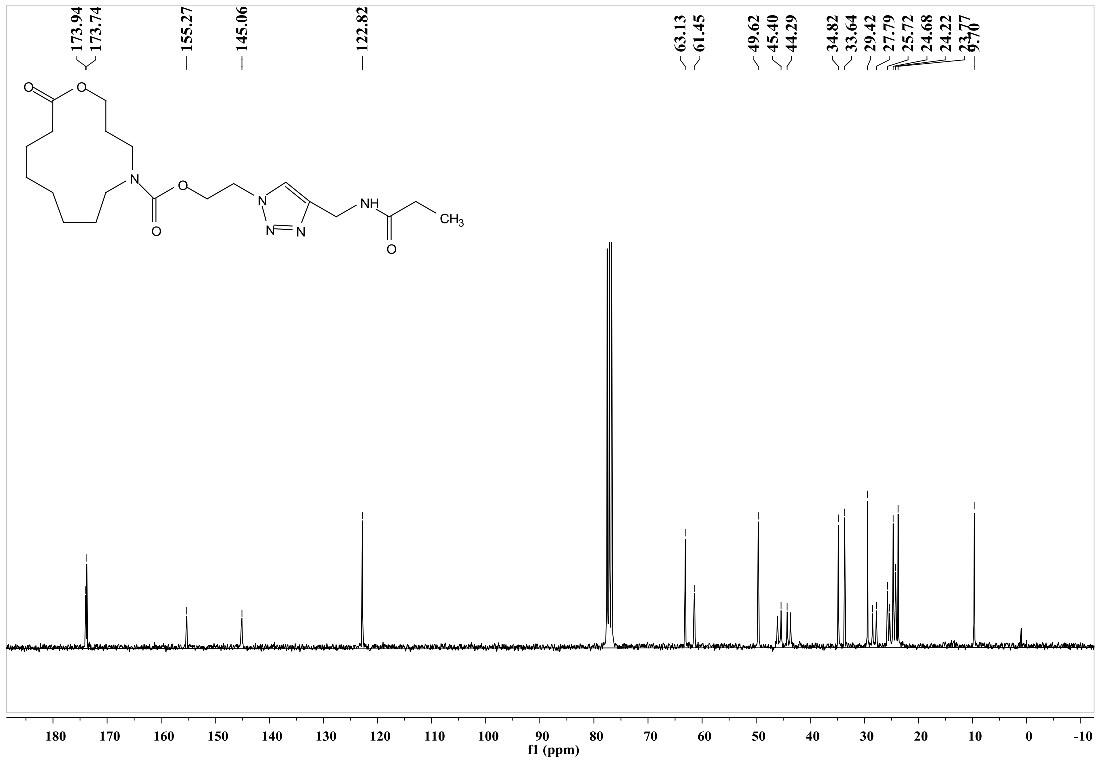
173.94
173.74
155.27
145.06
122.82
63.13
61.45
49.62
45.40
44.29
34.82
33.64
29.42
27.79
25.72
24.68
24.22
23.77
9.70
O
O
N
O
O
N
N
N
NH
O
CH_3
180
170
160
150
140
130
120
110
100
90
80
70
60
50
40
30
20
10
0
-10
f1 (ppm)

Z13-2

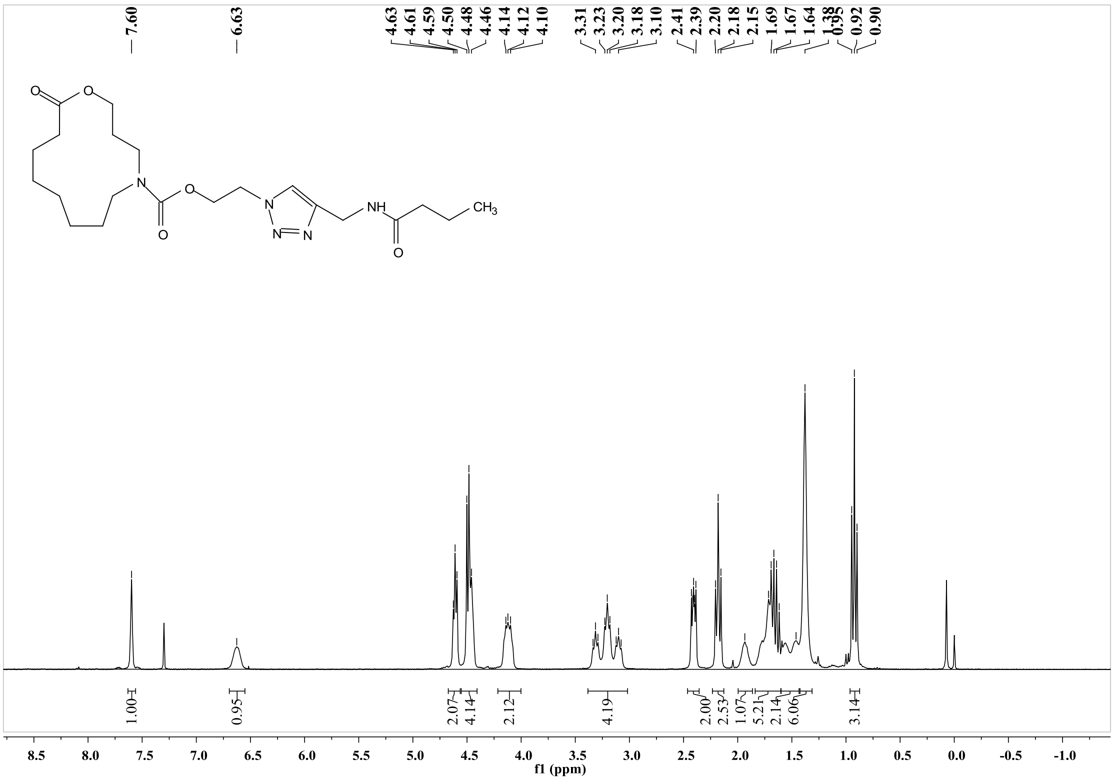
7.60
6.63
4.63
4.61
4.59
4.50
4.48
4.46
4.14
4.12
4.10
3.31
3.23
3.20
3.18
3.10
2.41
2.39
2.20
2.18
2.15
1.69
1.67
1.64
1.38
0.95
0.92
0.90
1.00
0.95
2.07
4.14
2.12
4.19
2.00
2.53
1.07
5.21
2.14
6.06
3.14
f1 (ppm)

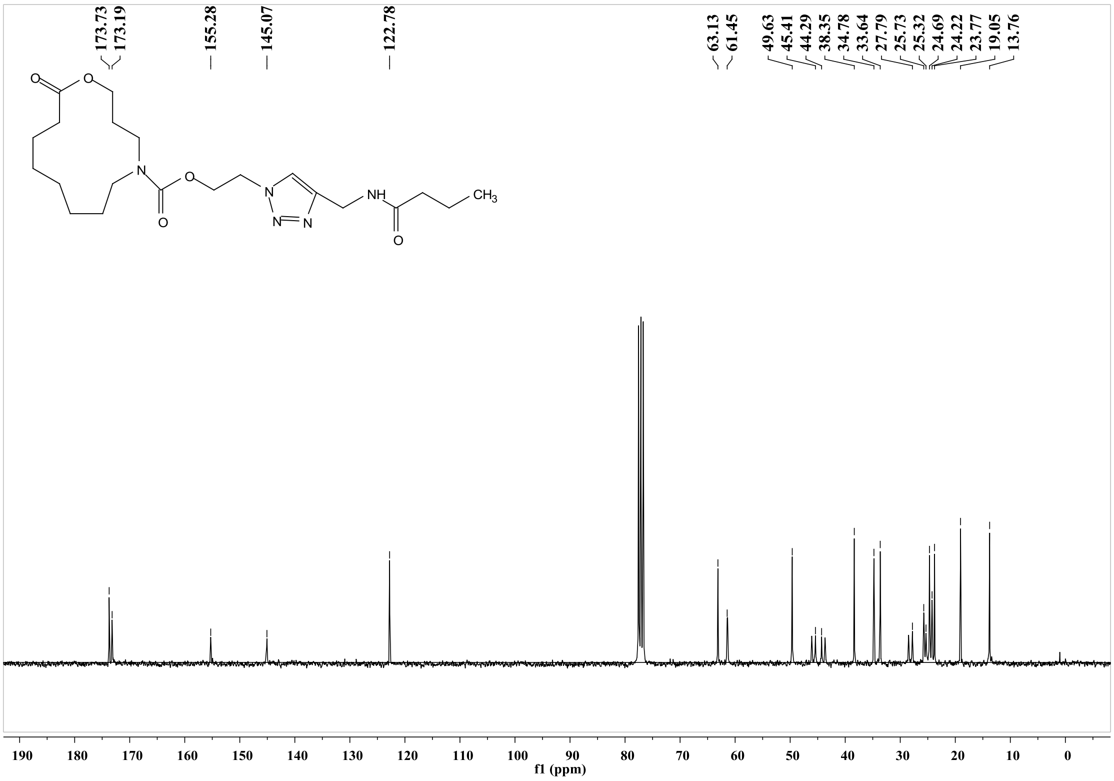
173.73
173.19
155.28
145.07
122.78
63.13
61.45
49.63
45.41
44.29
38.35
34.78
33.64
27.79
25.73
25.32
24.69
24.22
23.77
19.05
13.76
f1 (ppm)

Z13-3

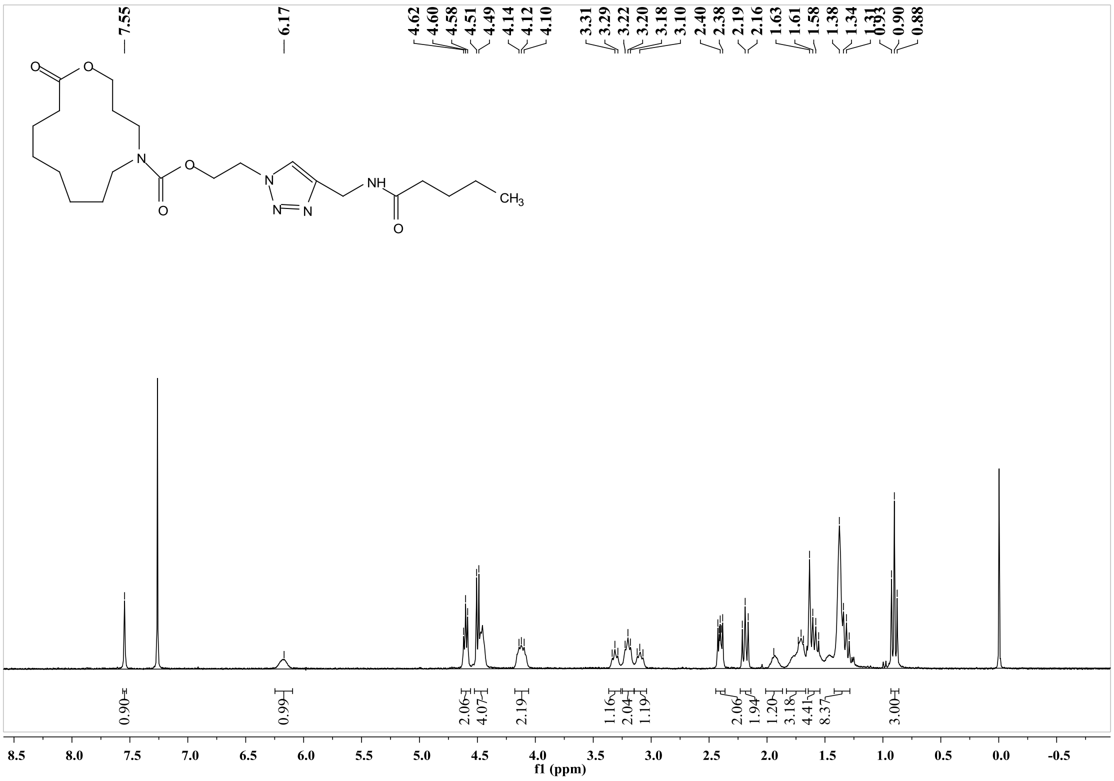

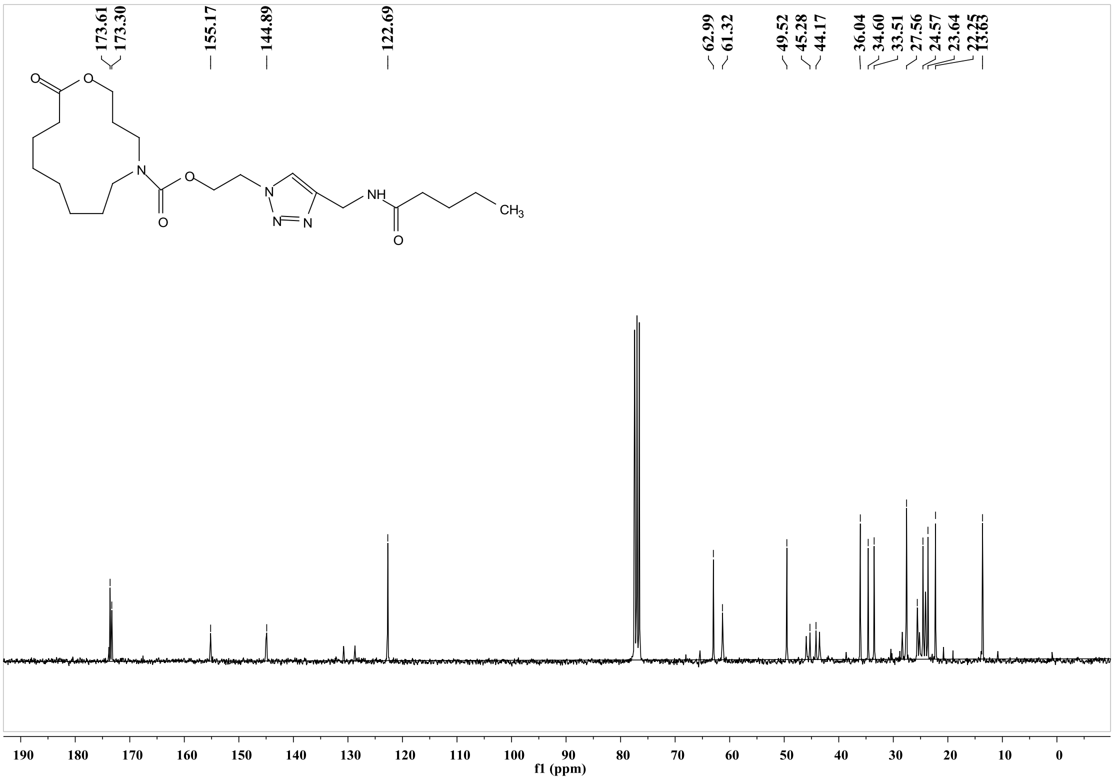

Z13-4

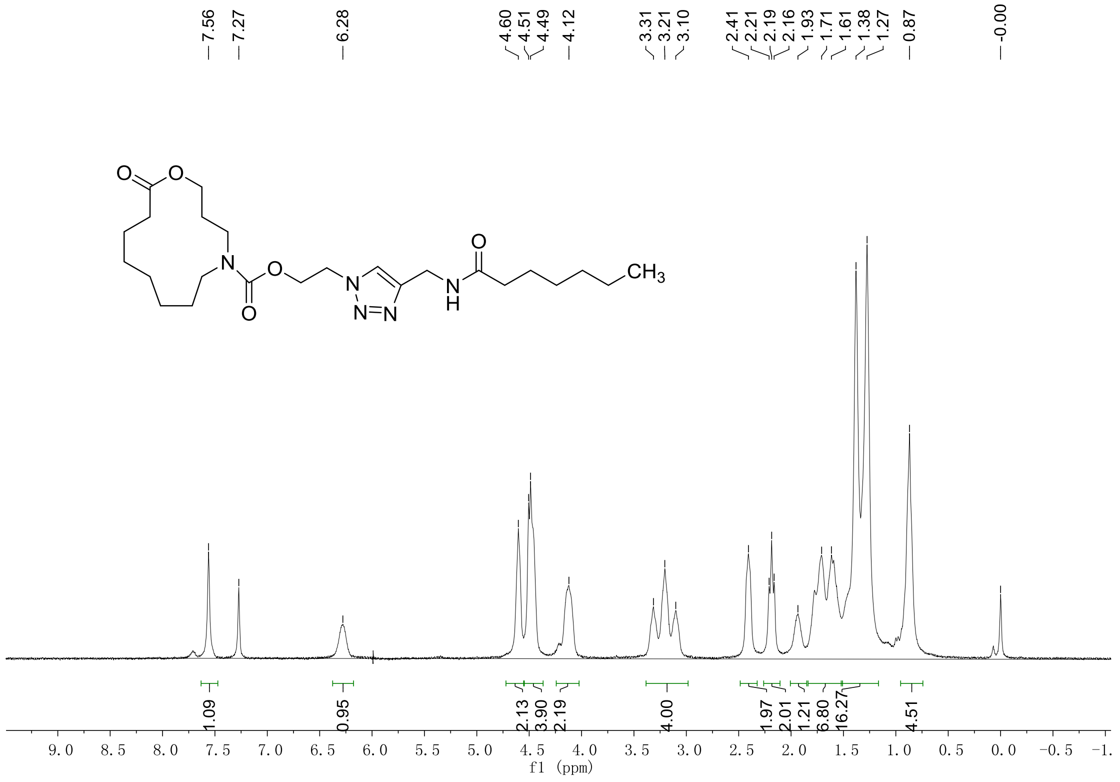

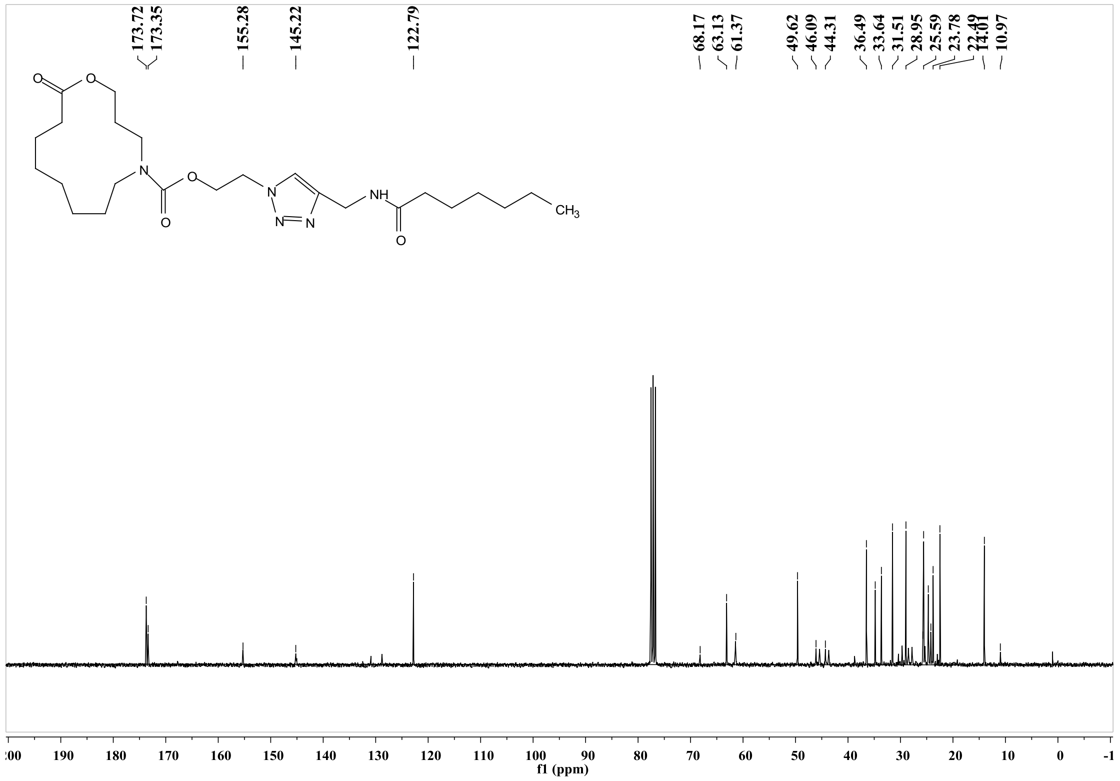

Z13-5

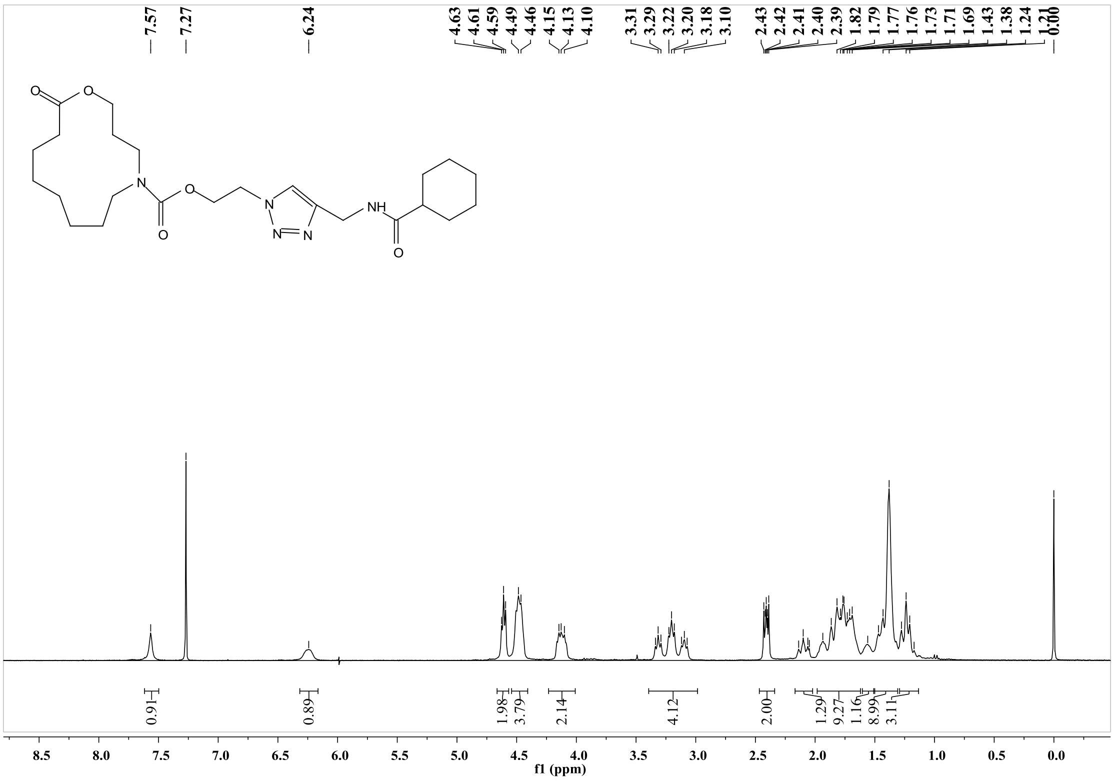

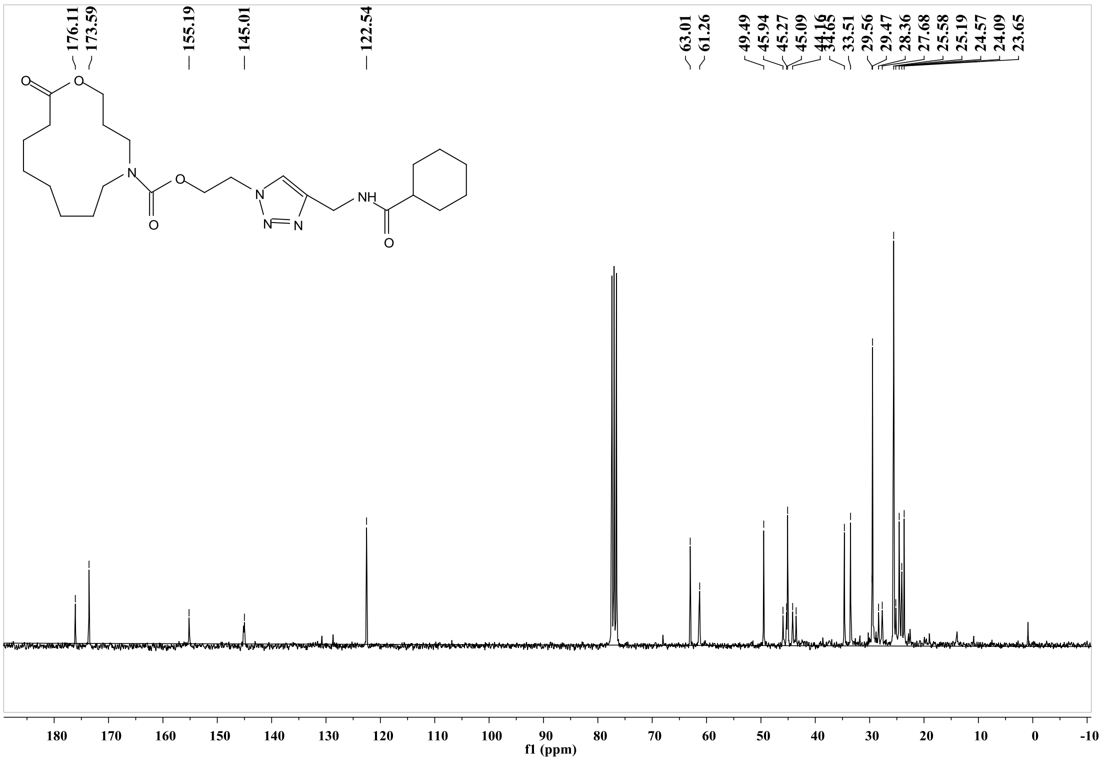

Z13-6

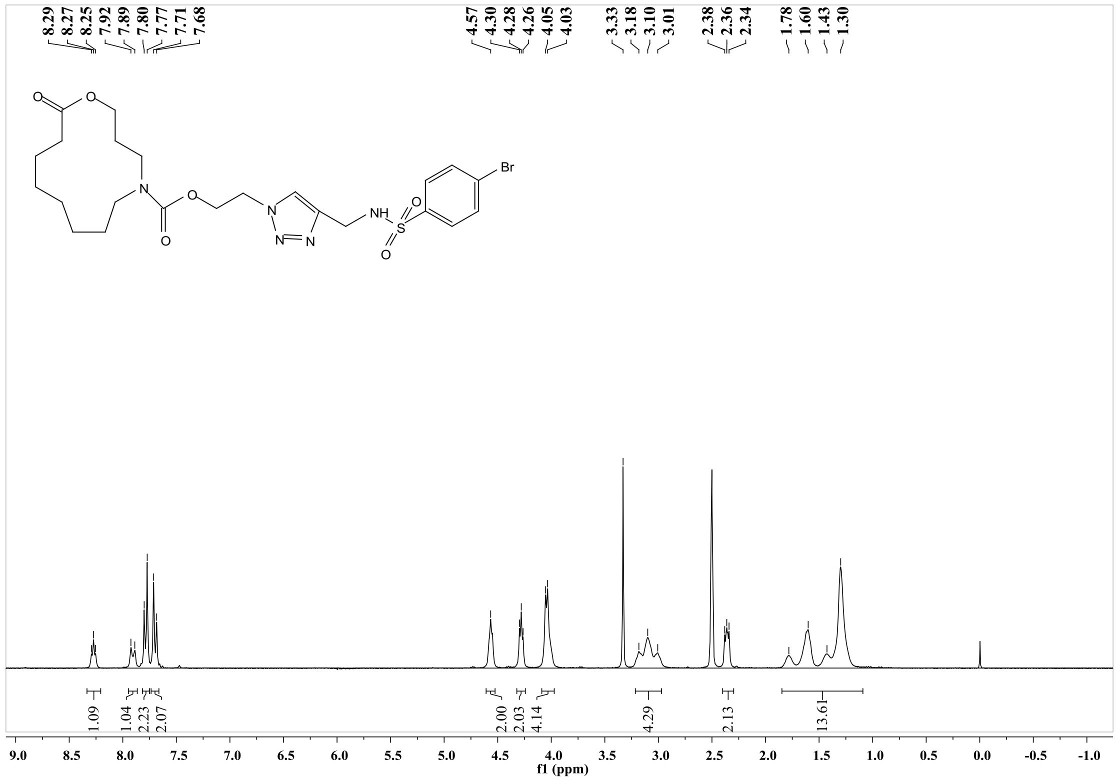

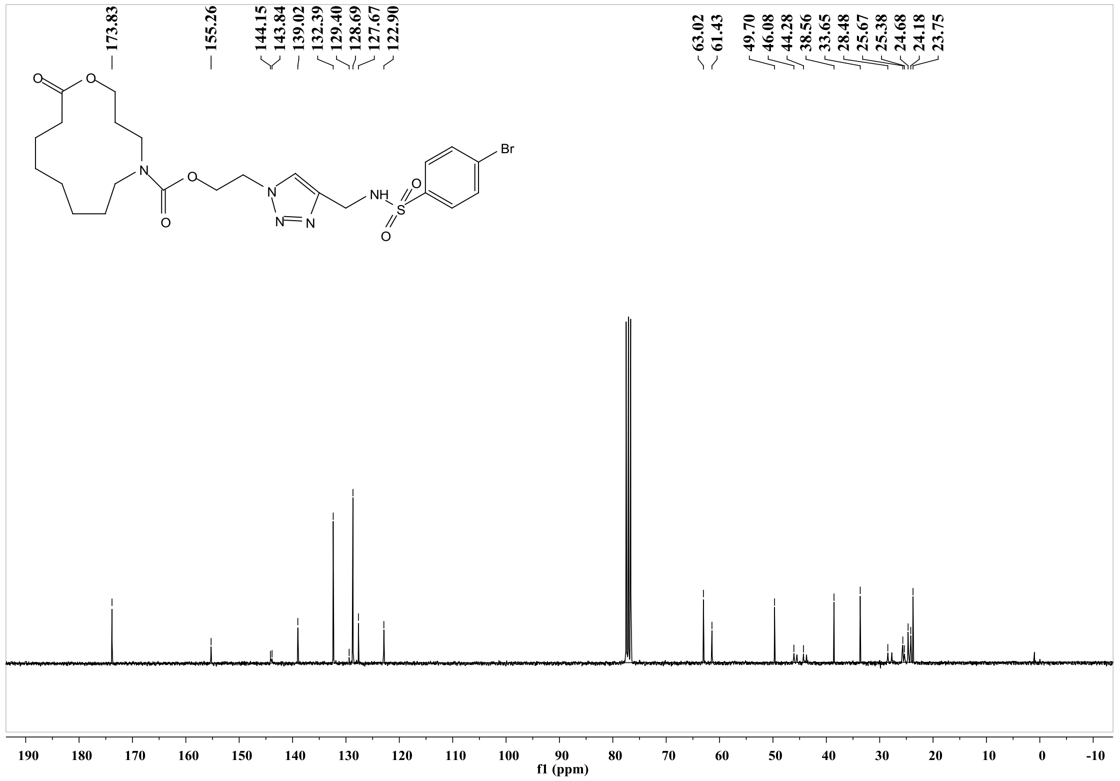

Z13-7

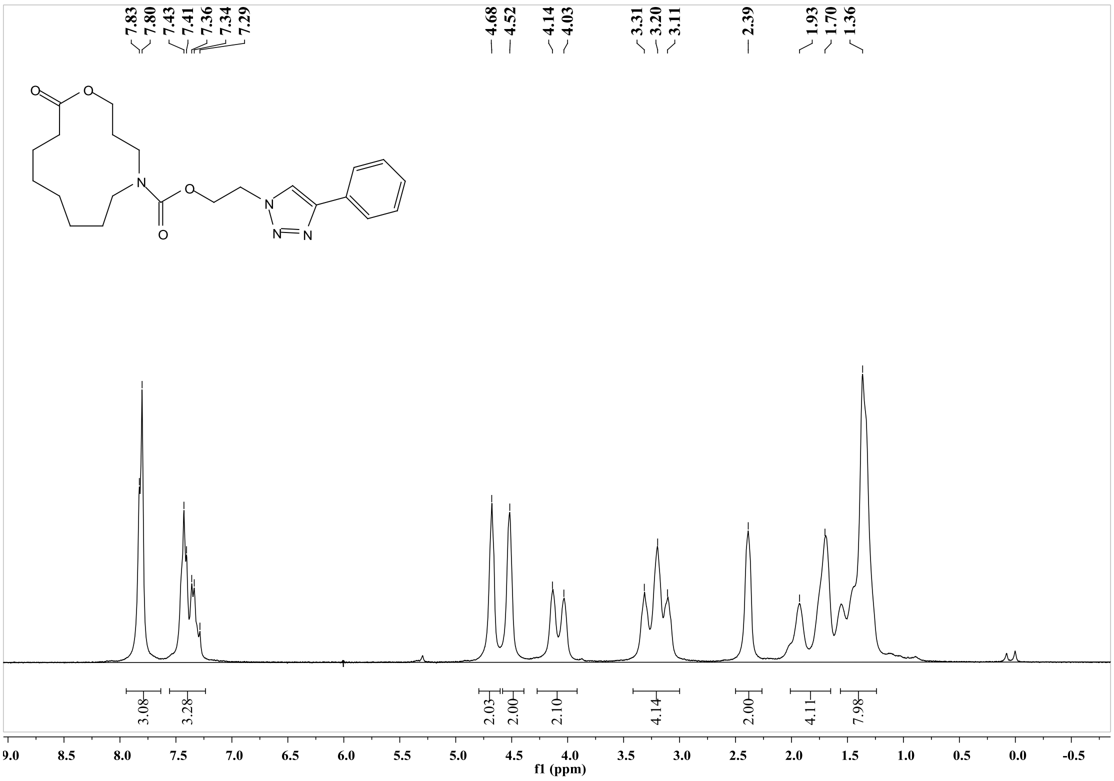
7.83
7.80
7.43
7.41
7.36
7.34
7.29
4.68
4.52
4.14
4.03
3.31
3.20
3.11
2.39
1.93
1.70
1.36
O
O
N
O
O
N
N
N
3.08
3.28
2.03
2.00
2.10
4.14
2.00
4.11
7.98
9.0 8.5 8.0 7.5 7.0 6.5 6.0 5.5 5.0 4.5 4.0 3.5 3.0 2.5 2.0 1.5 1.0 0.5 0.0 -0.5
f1 (ppm)

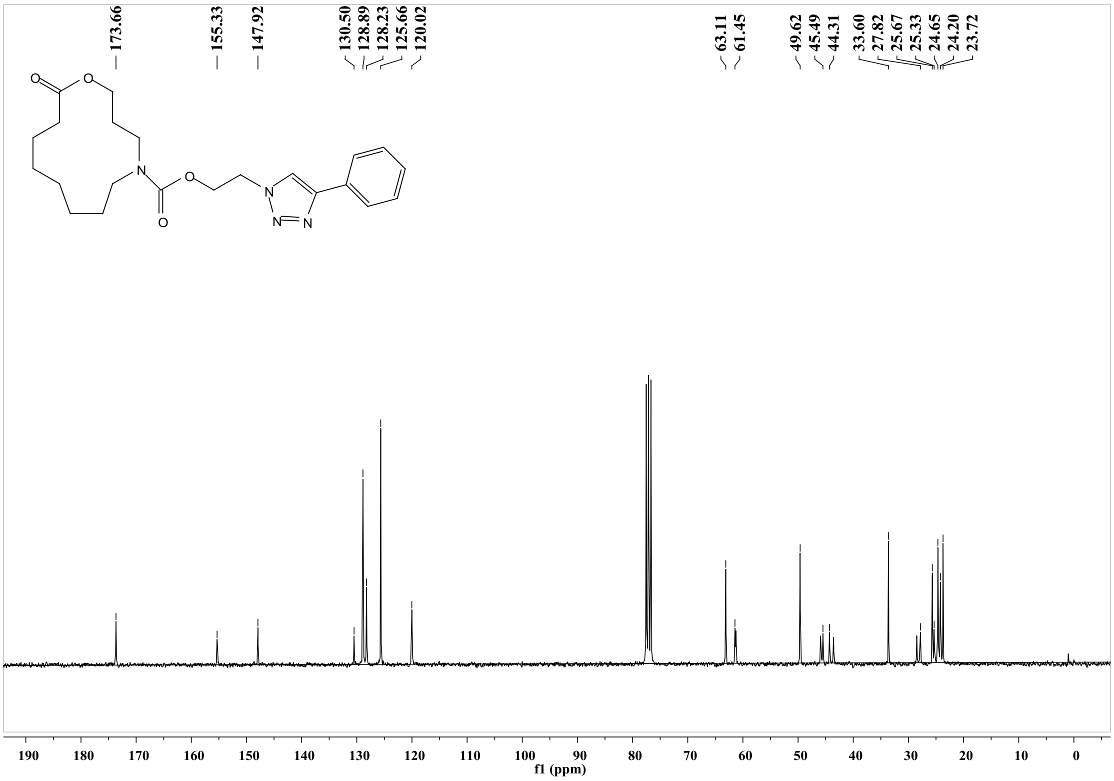
173.66
155.33
147.92
130.50
128.89
128.23
125.66
120.02
63.11
61.45
49.62
45.49
44.31
33.60
27.82
25.67
25.33
24.65
24.20
23.72
O
O
N
O
O
N
N
N
190 180 170 160 150 140 130 120 110 100 90 80 70 60 50 40 30 20 10 0
f1 (ppm)

Z13-8

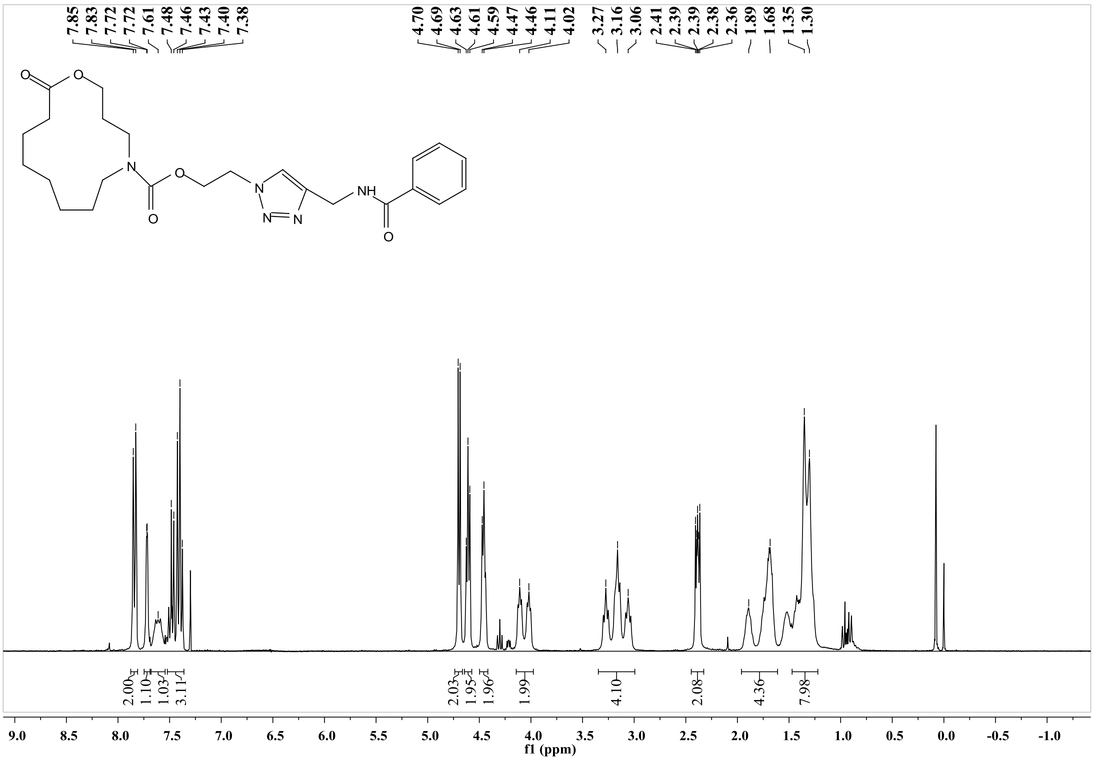

7.85
7.83
7.72
7.72
7.61
7.48
7.46
7.43
7.40
7.38
4.70
4.69
4.63
4.61
4.59
4.47
4.46
4.11
4.02
3.27
3.16
3.06
2.41
2.39
2.39
2.38
2.36
1.89
1.68
1.35
1.30
2.00
1.10
1.03
3.11
2.03
1.95
1.96
1.99
4.10
2.08
4.36
7.98
9.0
8.5
8.0
7.5
7.0
6.5
6.0
5.5
5.0
4.5
4.0
3.5
3.0
2.5
2.0
1.5
1.0
0.5
0.0
-0.5
-1.0
f1 (ppm)

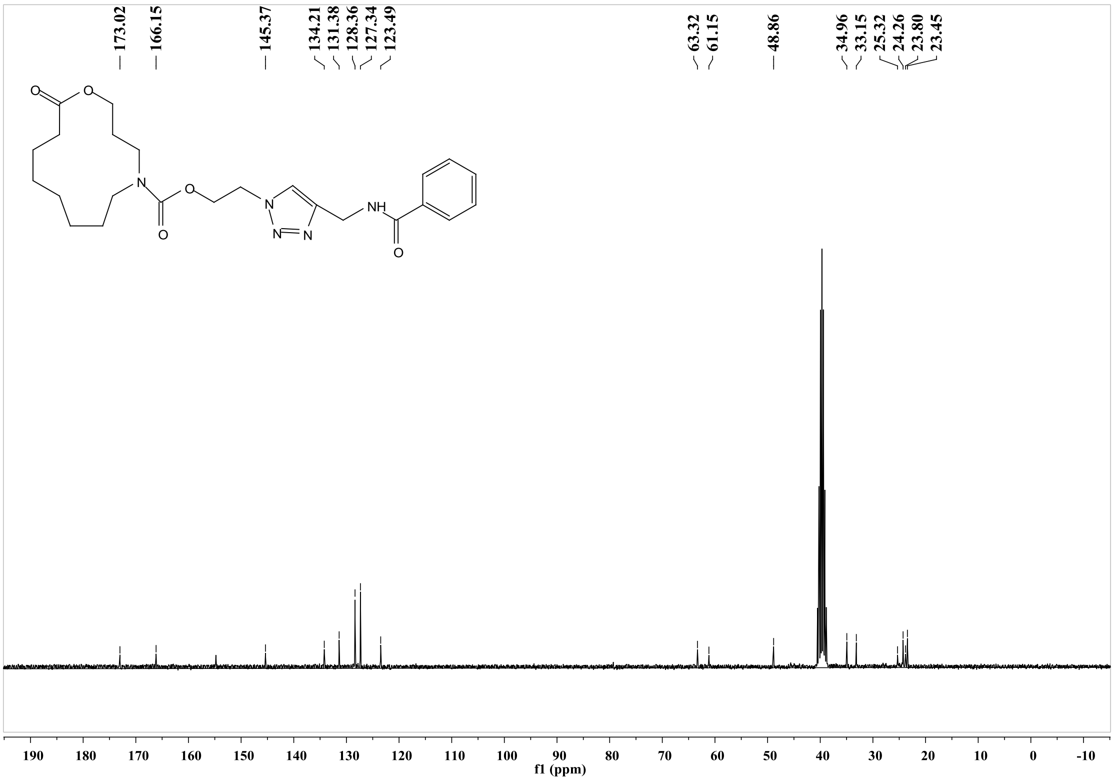

173.02
166.15
145.37
134.21
131.38
128.36
127.34
123.49
63.32
61.15
48.86
34.96
33.15
25.32
24.26
23.80
23.45
190
180
170
160
150
140
130
120
110
100
90
80
70
60
50
40
30
20
10
0
-10
f1 (ppm)

Z13-9

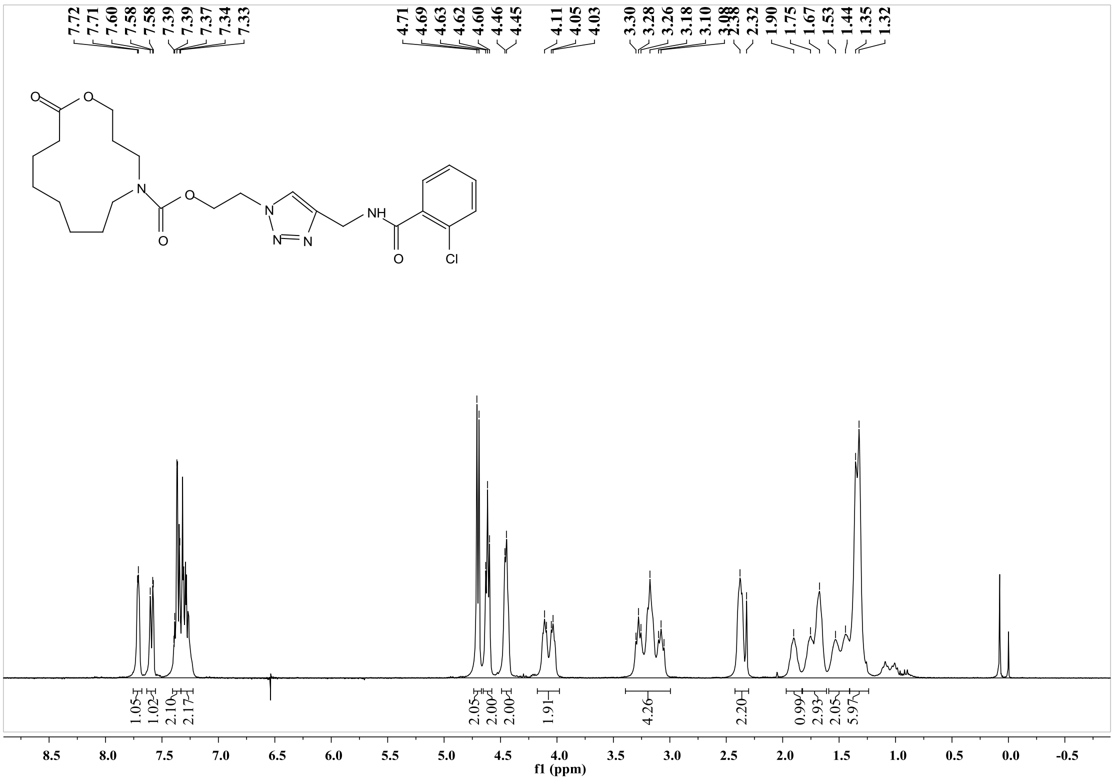
7.72
7.71
7.60
7.58
7.58
7.39
7.39
7.37
7.34
7.33
4.71
4.69
4.63
4.62
4.60
4.46
4.45
4.11
4.05
4.03
3.30
3.28
3.26
3.18
3.10
3.08
2.38
2.32
1.90
1.75
1.67
1.53
1.44
1.35
1.32
O
O
N
O
O
N
N
N
NH
O
Cl
1.05
1.02
2.10
2.17
2.05
2.00
2.00
1.91
4.26
2.20
0.99
2.93
2.05
5.97
8.5
8.0
7.5
7.0
6.5
6.0
5.5
5.0
4.5
4.0
3.5
3.0
2.5
2.0
1.5
1.0
0.5
0.0
-0.5
f1 (ppm)

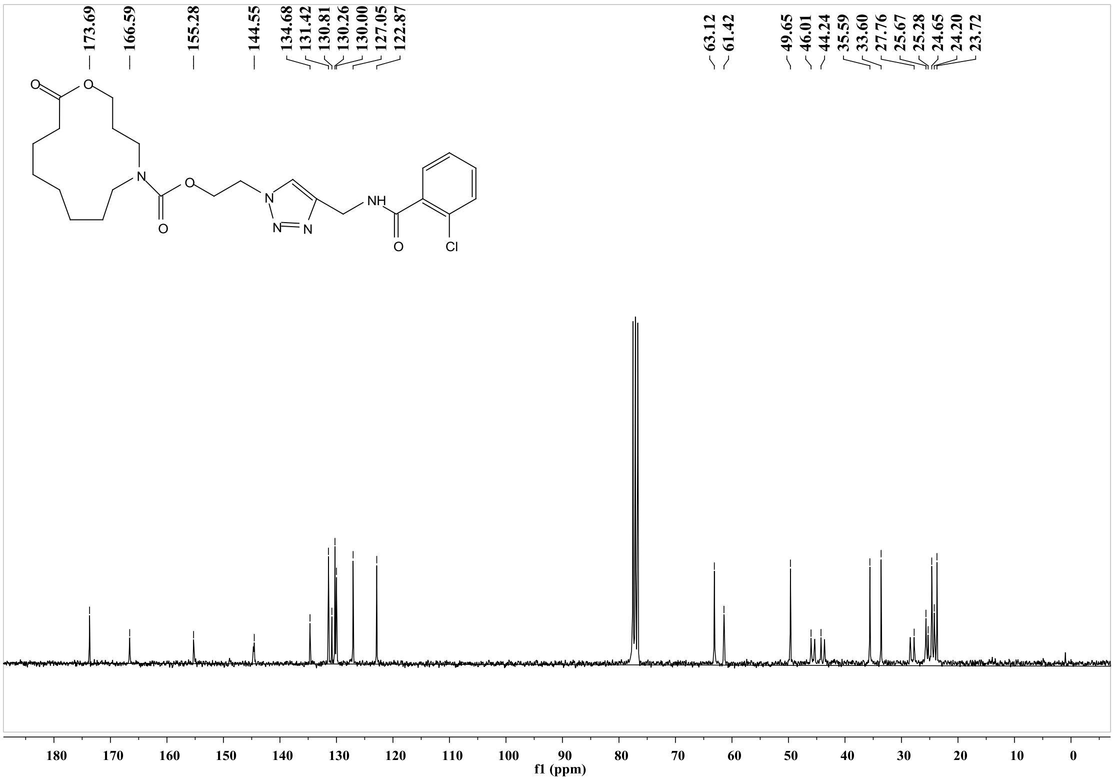
173.69
166.59
155.28
144.55
134.68
131.42
130.81
130.26
130.00
127.05
122.87
63.12
61.42
49.65
46.01
44.24
35.59
33.60
27.76
25.67
25.28
24.65
24.20
23.72
O
O
N
O
O
N
N
N
NH
O
Cl
180
170
160
150
140
130
120
110
100
90
80
70
60
50
40
30
20
10
0
f1 (ppm)

Z13-10

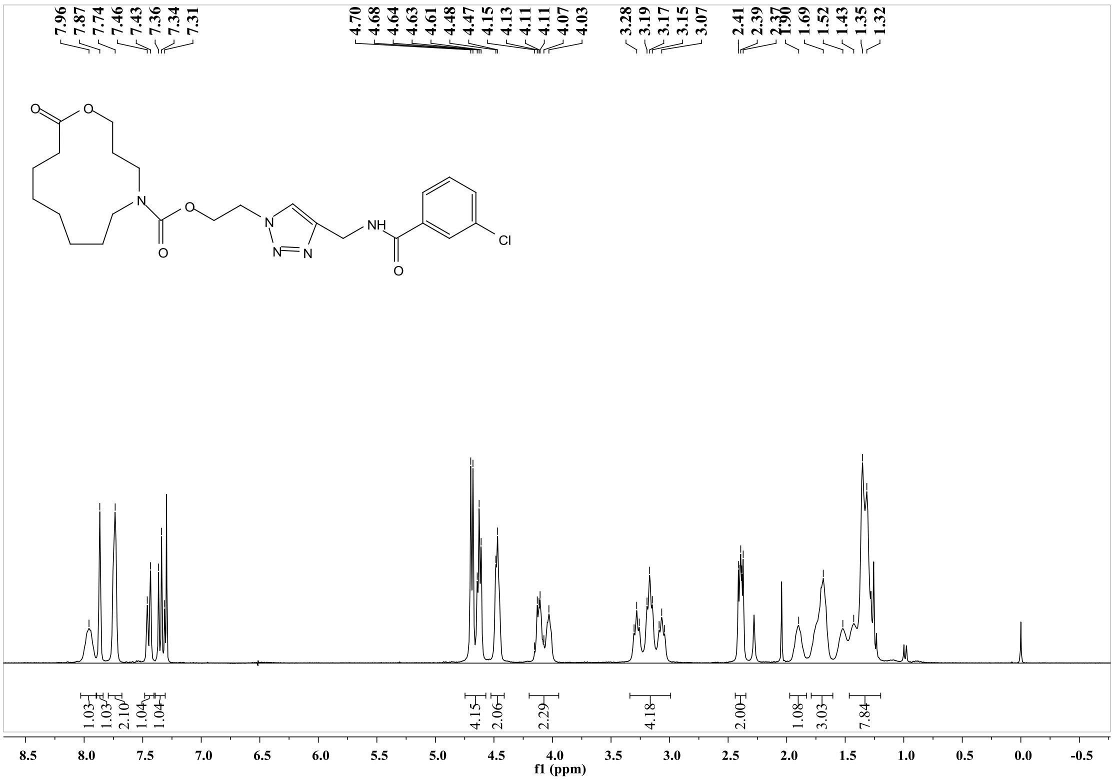
7.96
7.87
7.74
7.46
7.43
7.36
7.34
7.31
4.70
4.68
4.64
4.63
4.61
4.48
4.47
4.15
4.13
4.11
4.11
4.07
4.03
3.28
3.19
3.17
3.15
3.07
2.41
2.39
2.37
1.90
1.69
1.52
1.43
1.35
1.32
NH
O
N
N
N
N
O
O
O
O
Cl
1.03
1.03
2.10
1.04
1.04
4.15
2.06
2.29
4.18
2.00
1.08
3.03
7.84
8.5
8.0
7.5
7.0
6.5
6.0
5.5
5.0
4.5
4.0
3.5
3.0
2.5
2.0
1.5
1.0
0.5
0.0
-0.5
f1 (ppm)

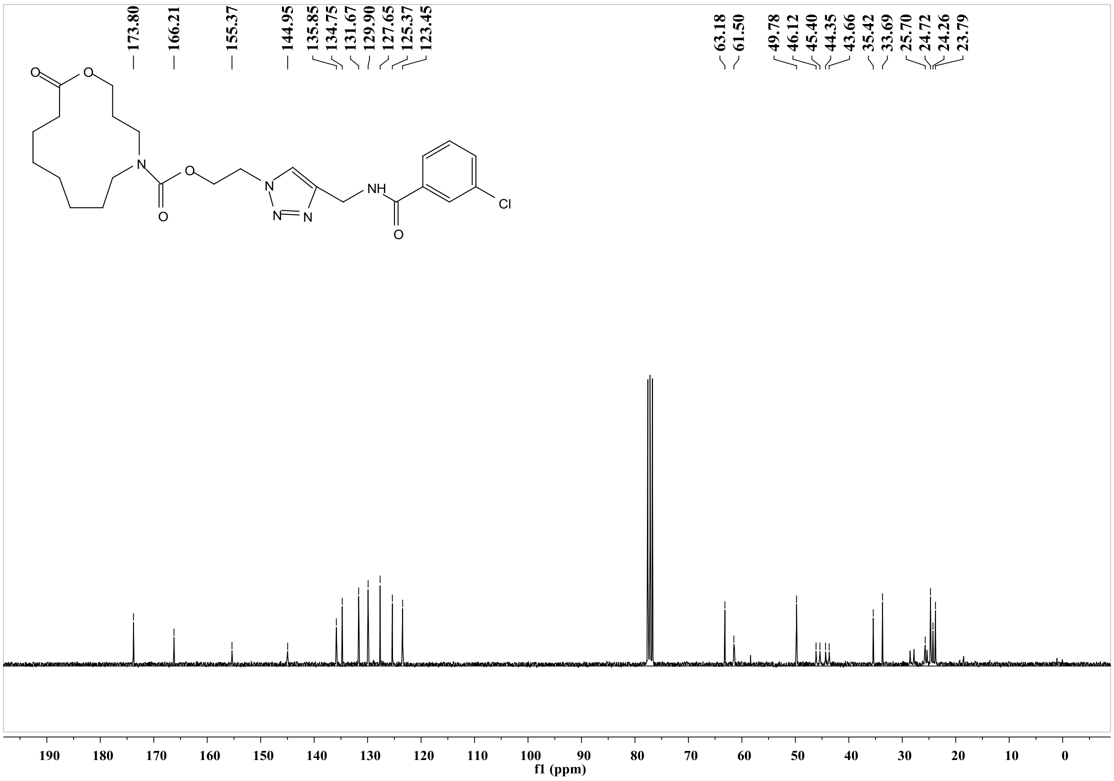
173.80
166.21
155.37
144.95
135.85
134.75
131.67
129.90
127.65
125.37
123.45
63.18
61.50
49.78
46.12
45.40
44.35
43.66
35.42
33.69
25.70
24.72
24.26
23.79
NH
O
N
N
N
N
O
O
O
O
Cl
190
180
170
160
150
140
130
120
110
100
90
80
70
60
50
40
30
20
10
0
f1 (ppm)

Z13-11

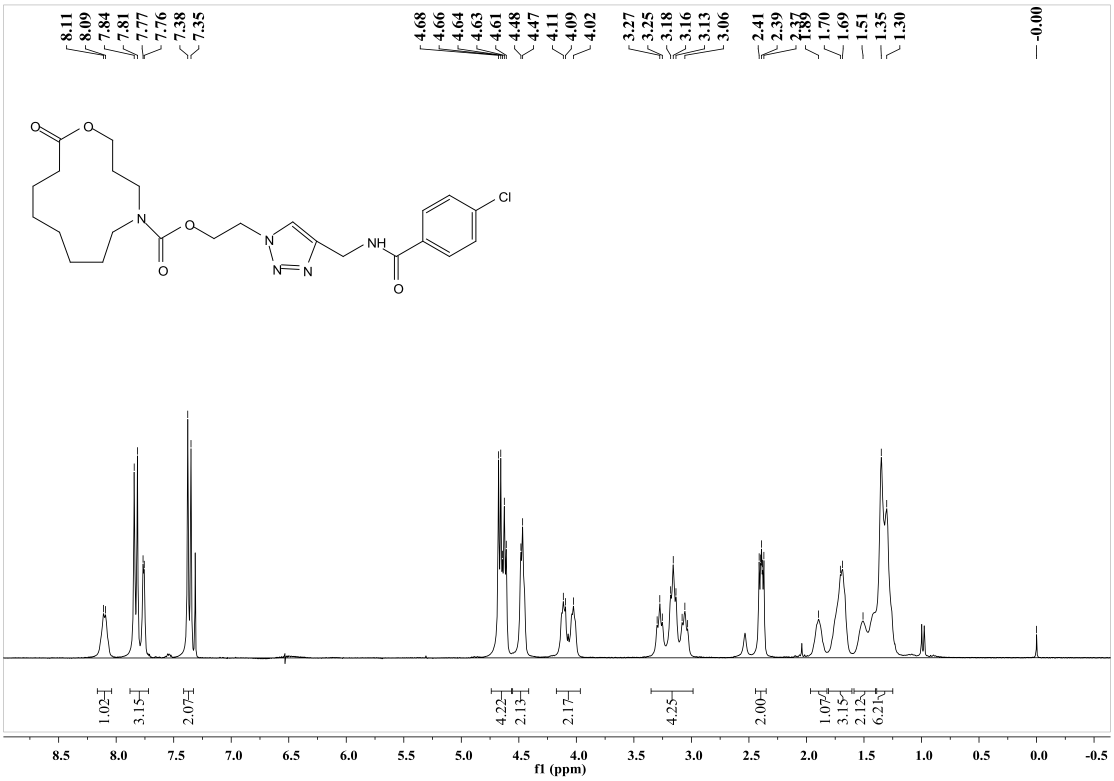
8.11 8.09 7.84 7.81 7.77 7.76 7.38 7.35
4.68 4.66 4.64 4.63 4.61 4.48 4.47 4.11 4.09 4.02
3.27 3.25 3.18 3.16 3.13 3.06
2.41 2.39 2.37 1.89 1.70 1.69 1.51 1.35 1.30
-0.00
O
O
N
O
O
N
N
N
NH
O
Cl
1.02 3.15 2.07 4.22 2.13 2.17 4.25 2.00 1.07 3.15 2.12 6.21
8.5 8.0 7.5 7.0 6.5 6.0 5.5 5.0 4.5 4.0 3.5 3.0 2.5 2.0 1.5 1.0 0.5 0.0 -0.5
f1 (ppm)

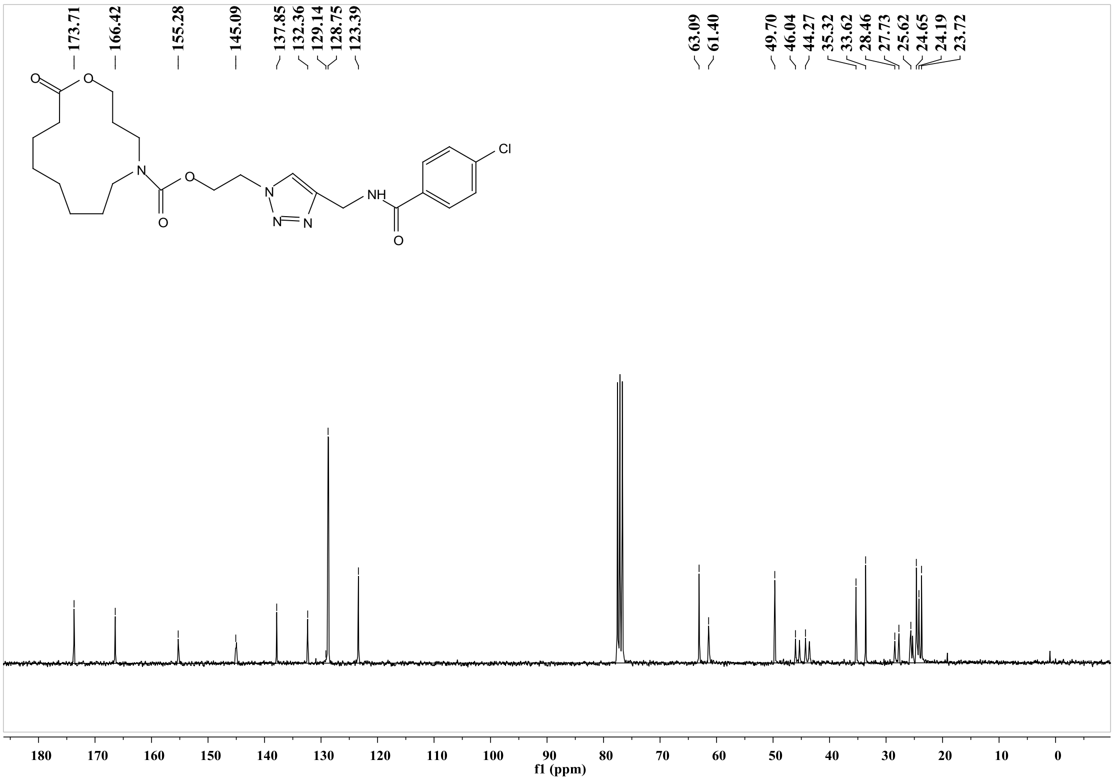
173.71 166.42 155.28 145.09 137.85 132.36 129.14 128.75 123.39
63.09 61.40
49.70 46.04 44.27 35.32 33.62 28.46 27.73 25.62 24.65 24.19 23.72
O
O
N
O
O
N
N
N
NH
O
Cl
180 170 160 150 140 130 120 110 100 90 80 70 60 50 40 30 20 10 0
f1 (ppm)

Z13-12

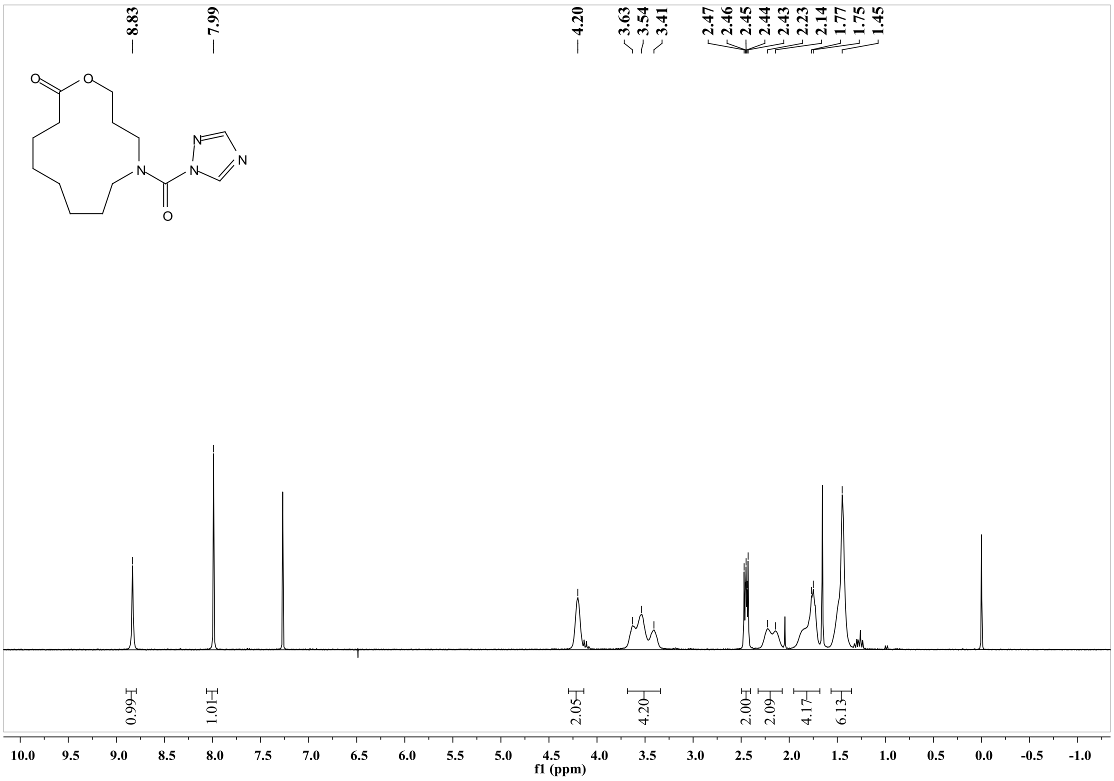

8.83
7.99
4.20
3.63
3.54
3.41
2.47
2.46
2.45
2.44
2.43
2.23
2.14
1.77
1.75
1.45
0.99
1.01
2.05
4.20
2.00
2.09
4.17
6.13
f1 (ppm)

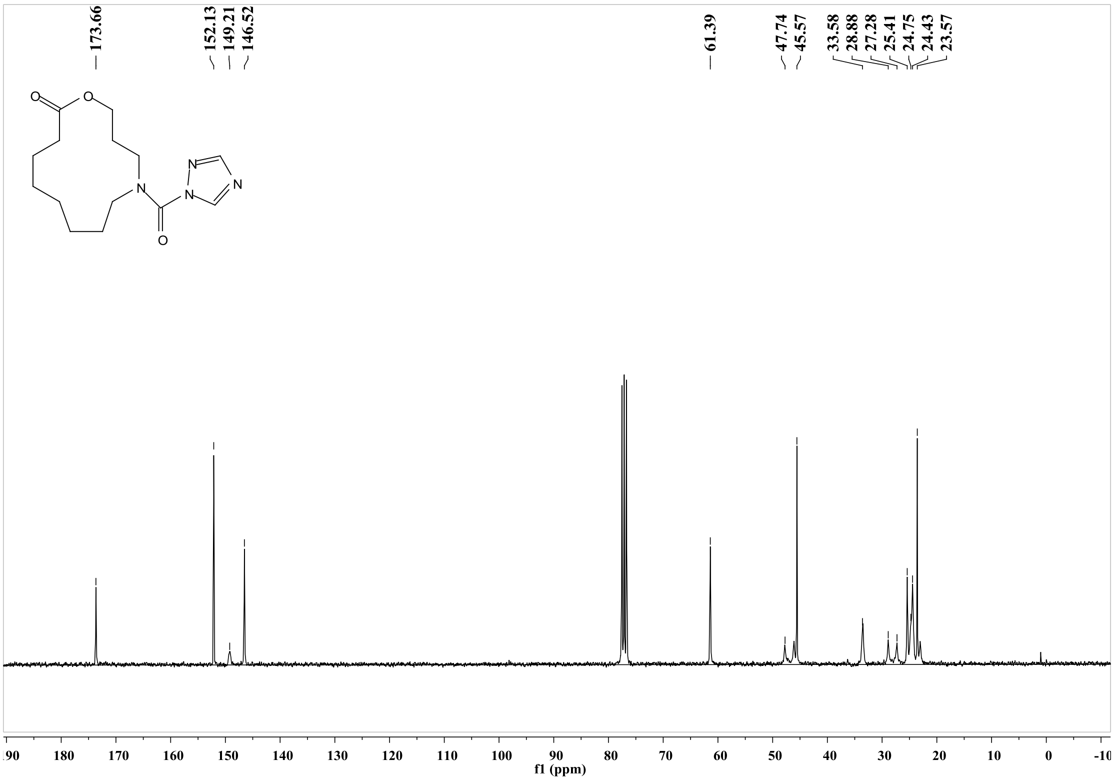

173.66
152.13
149.21
146.52
61.39
47.74
45.57
33.58
28.88
27.28
25.41
24.75
24.43
23.57
f1 (ppm)

Z13-13

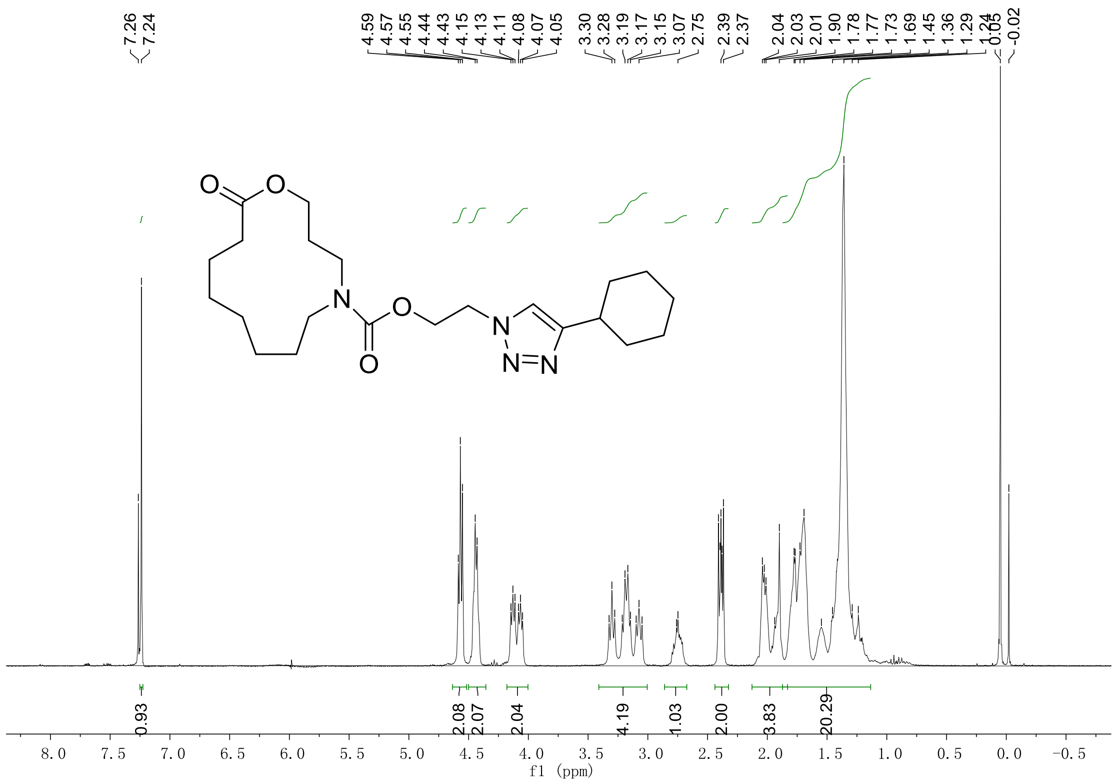
7.26
7.24
4.59 4.57 4.55 4.44 4.43 4.15 4.13 4.11 4.08 4.07 4.05
3.30 3.28 3.19 3.17 3.15 3.07 2.75
2.39 2.37
2.04 2.03 2.01 1.90 1.78 1.77 1.73 1.69 1.45 1.36 1.29 1.24 0.05
-0.02
O
O
N
O
O
N
N=N
0.93
2.08
2.07
2.04
4.19
1.03
2.00
3.83
20.29
8.0 7.5 7.0 6.5 6.0 5.5 5.0 4.5 4.0 3.5 3.0 2.5 2.0 1.5 1.0 0.5 0.0 -0.5
f1 (ppm)

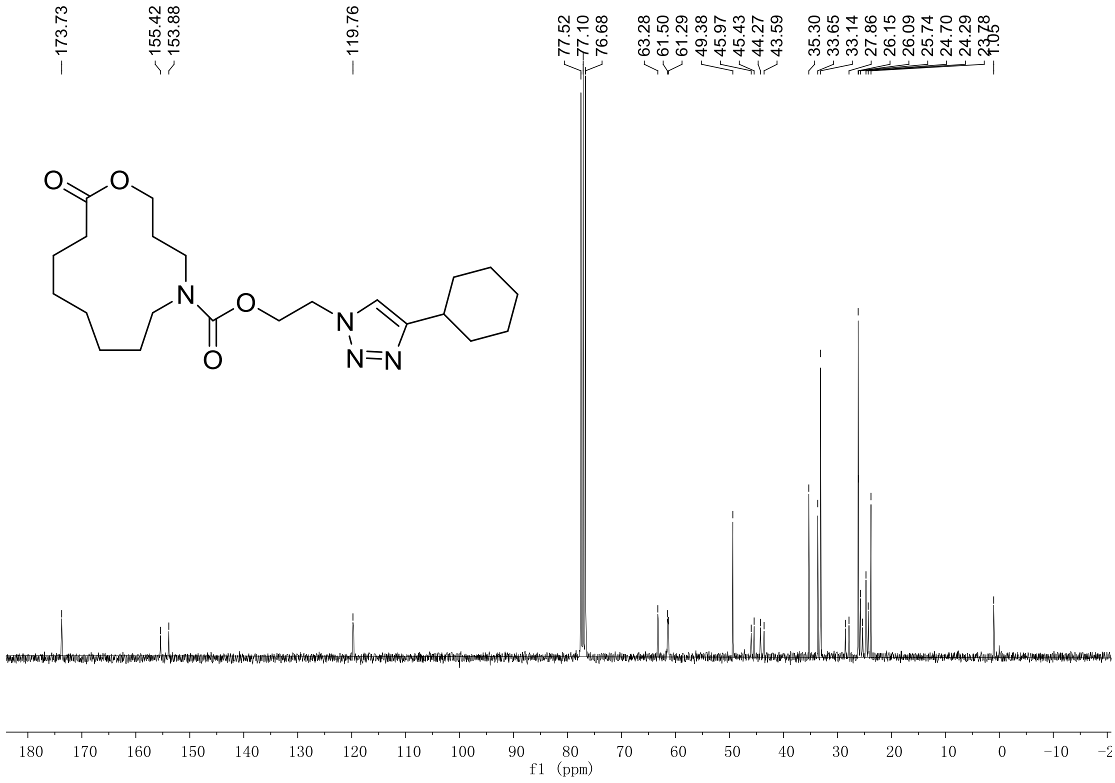
173.73
155.42
153.88
119.76
77.52
77.10
76.68
63.28 61.50 61.29 49.38 45.97 45.43 44.27 43.59
35.30 33.65 33.14 27.86 26.15 26.09 25.74 24.70 24.29 23.78 1.05
O
O
N
O
O
N
N=N
180 170 160 150 140 130 120 110 100 90 80 70 60 50 40 30 20 10 0 -10
f1 (ppm)

Z13-14

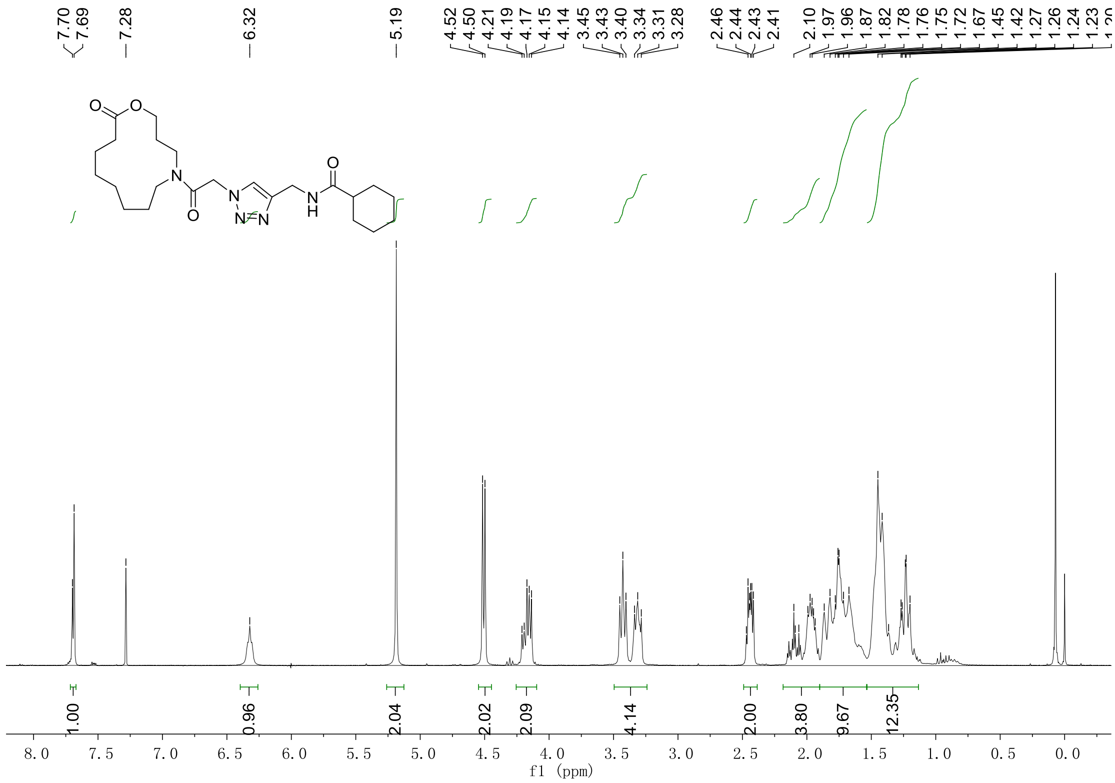

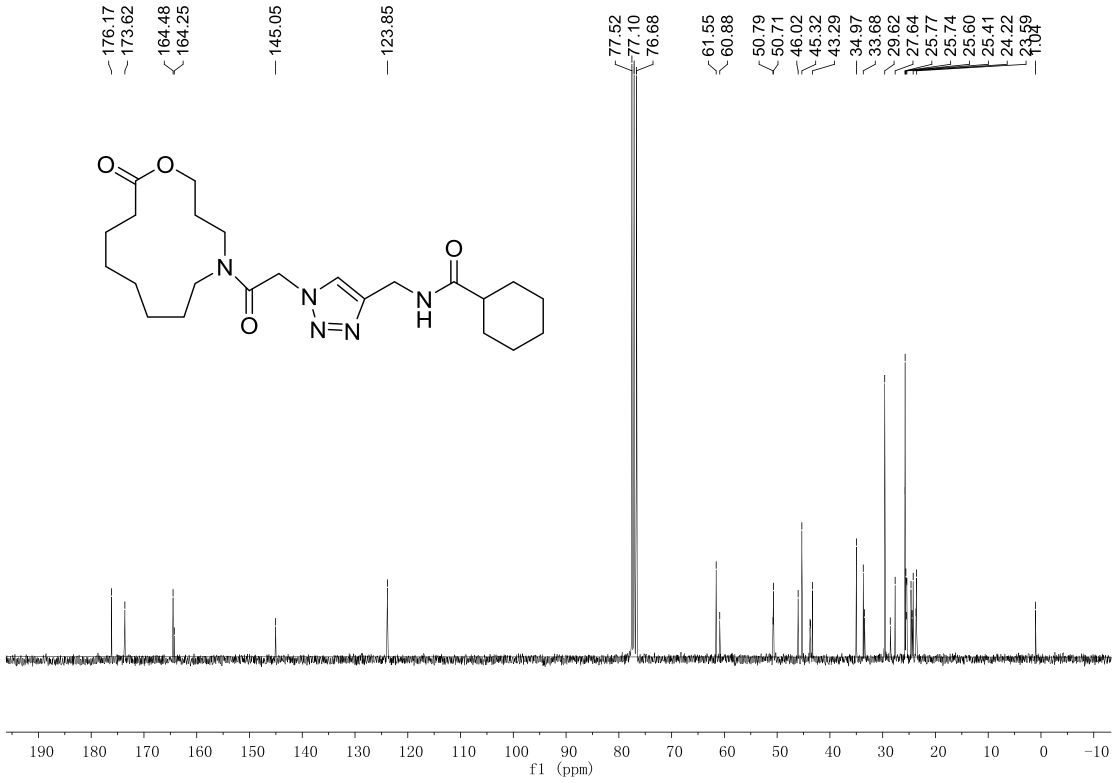

16-1

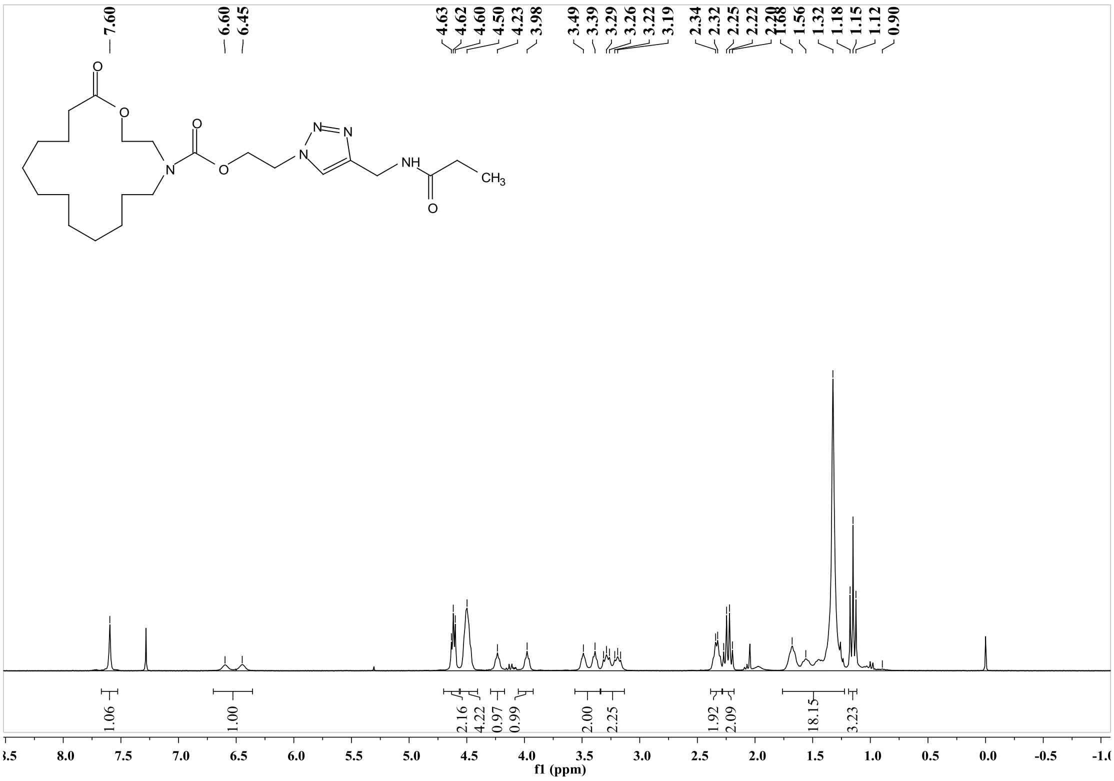

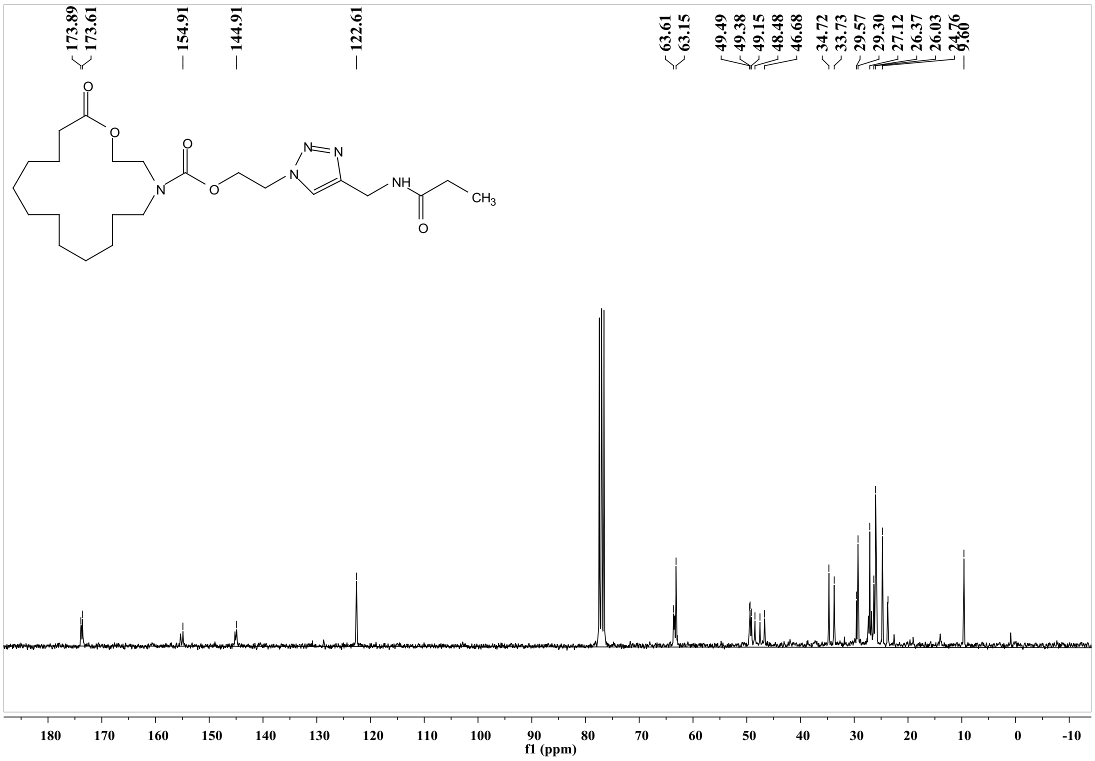
173.89
173.61
154.91
144.91
122.61
63.61
63.15
49.49
49.38
49.15
48.48
46.68
34.72
33.73
29.57
29.30
27.12
26.37
26.03
24.76
9.60
O
O
O
N
O
N
N
N
NH
O
CH_3
180
170
160
150
140
130
120
110
100
90
80
70
60
50
40
30
20
10
0
-10
f1 (ppm)

Z16-2

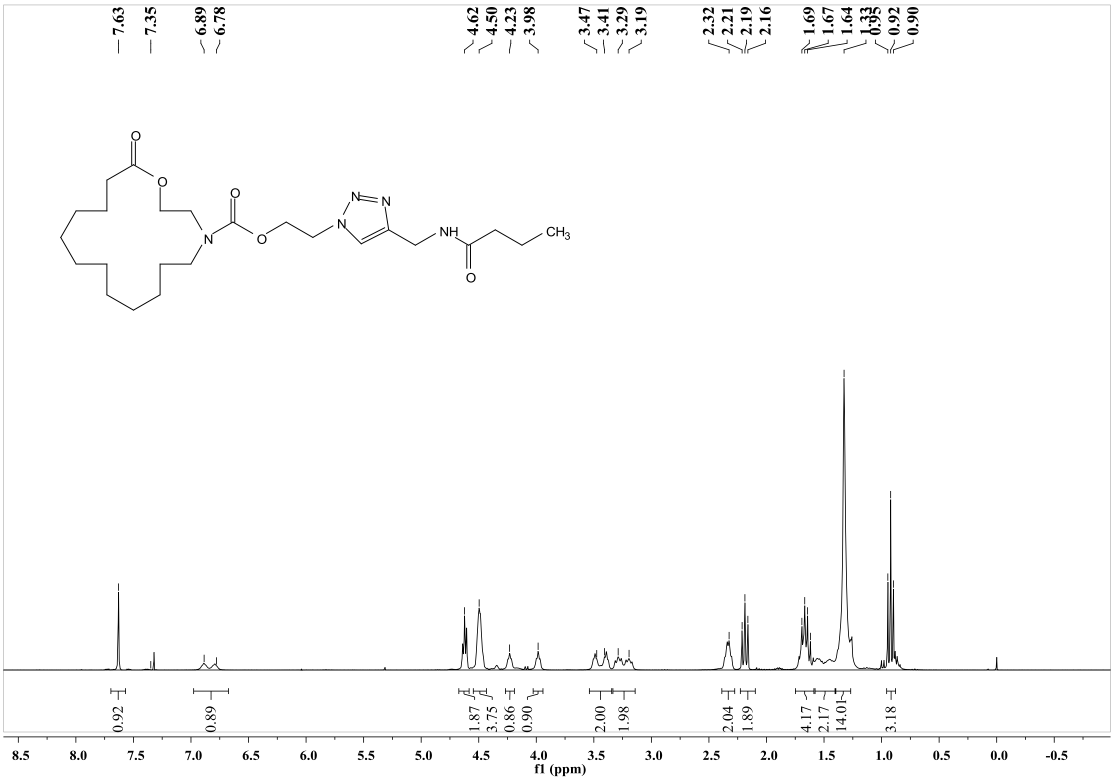
7.63
7.35
6.89
6.78
4.62
4.50
4.23
3.98
3.47
3.41
3.29
3.19
2.32
2.21
2.19
2.16
1.69
1.67
1.64
1.33
0.95
0.92
0.90
O
O
O
N
O
N
N
N
NH
O
CH3
0.92
0.89
1.87
3.75
0.86
0.90
2.00
1.98
2.04
1.89
4.17
2.17
14.01
3.18
8.5 8.0 7.5 7.0 6.5 6.0 5.5 5.0 4.5 4.0 3.5 3.0 2.5 2.0 1.5 1.0 0.5 0.0 -0.5
f1 (ppm)

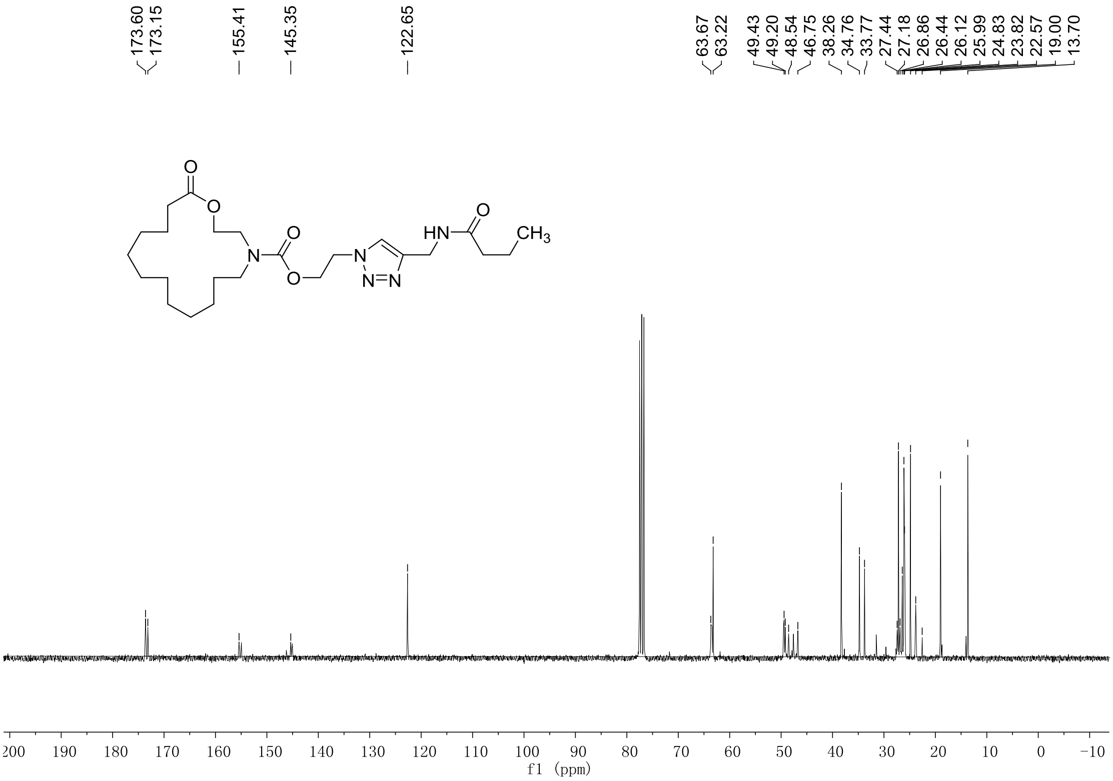
173.60
173.15
155.41
145.35
122.65
63.67
63.22
49.43
49.20
48.54
46.75
38.26
34.76
33.77
27.44
27.18
26.86
26.44
26.12
25.99
24.83
23.82
22.57
19.00
13.70
O
O
O
N
O
N
N
N
HN
O
CH3
200 190 180 170 160 150 140 130 120 110 100 90 80 70 60 50 40 30 20 10 0 −10
f1 (ppm)

Z16-3

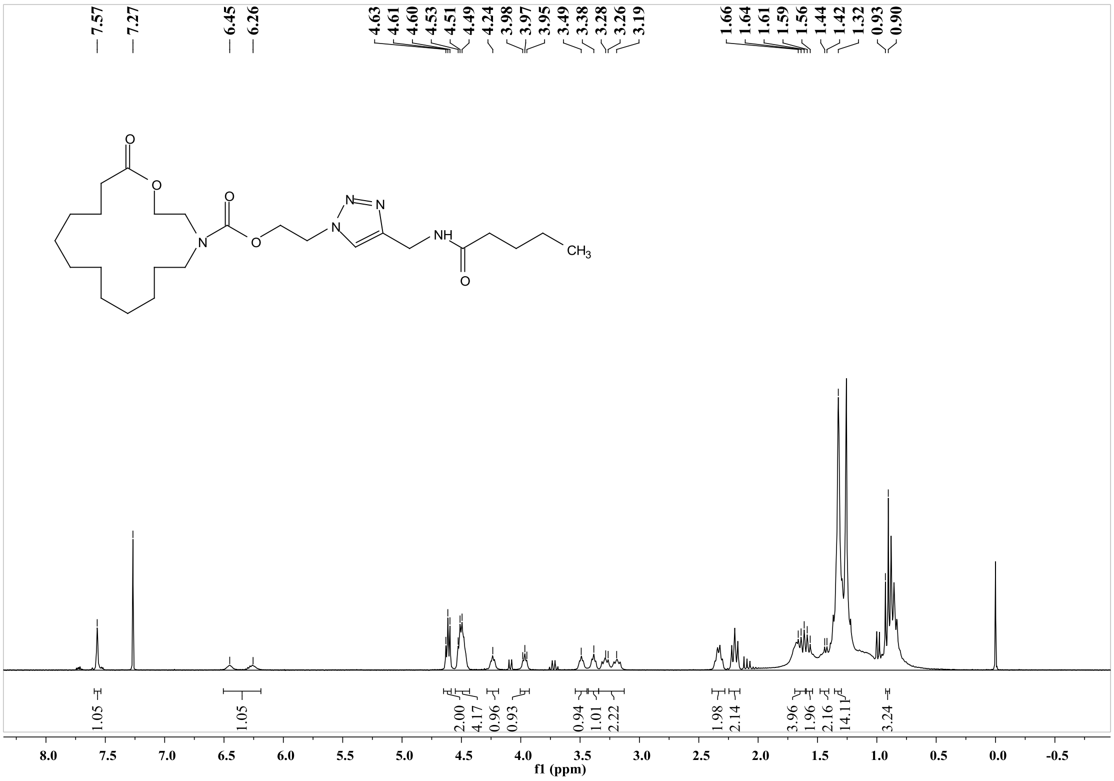

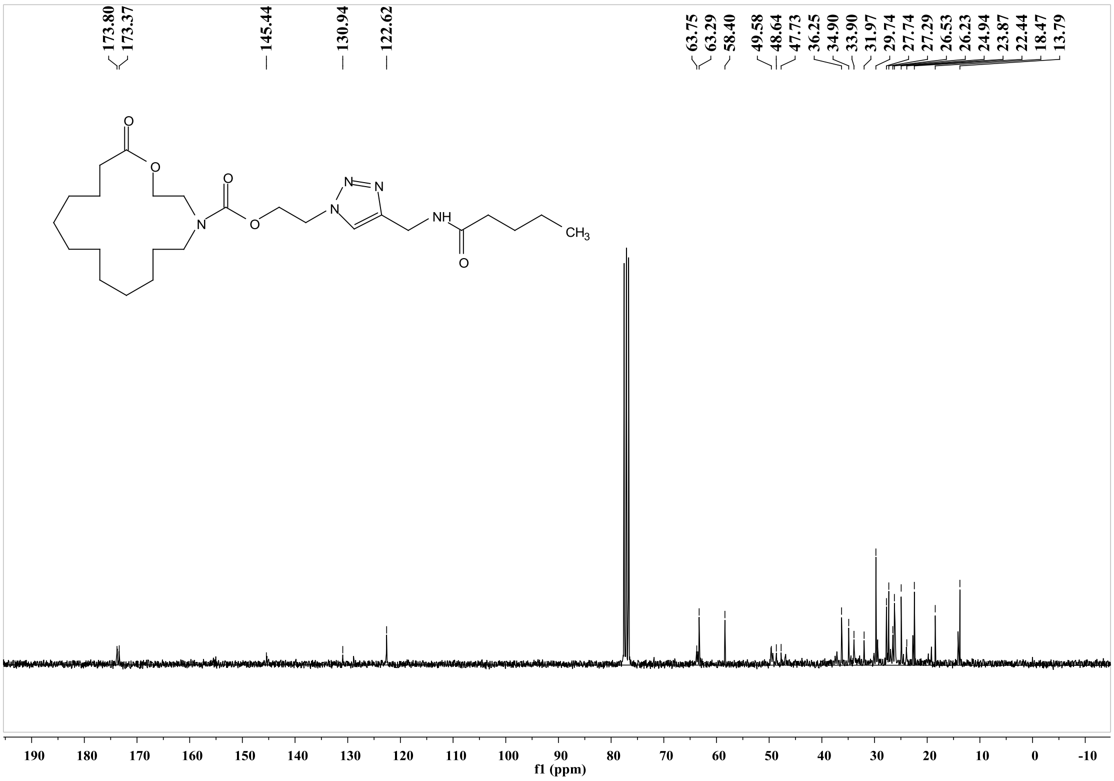

Z16-4

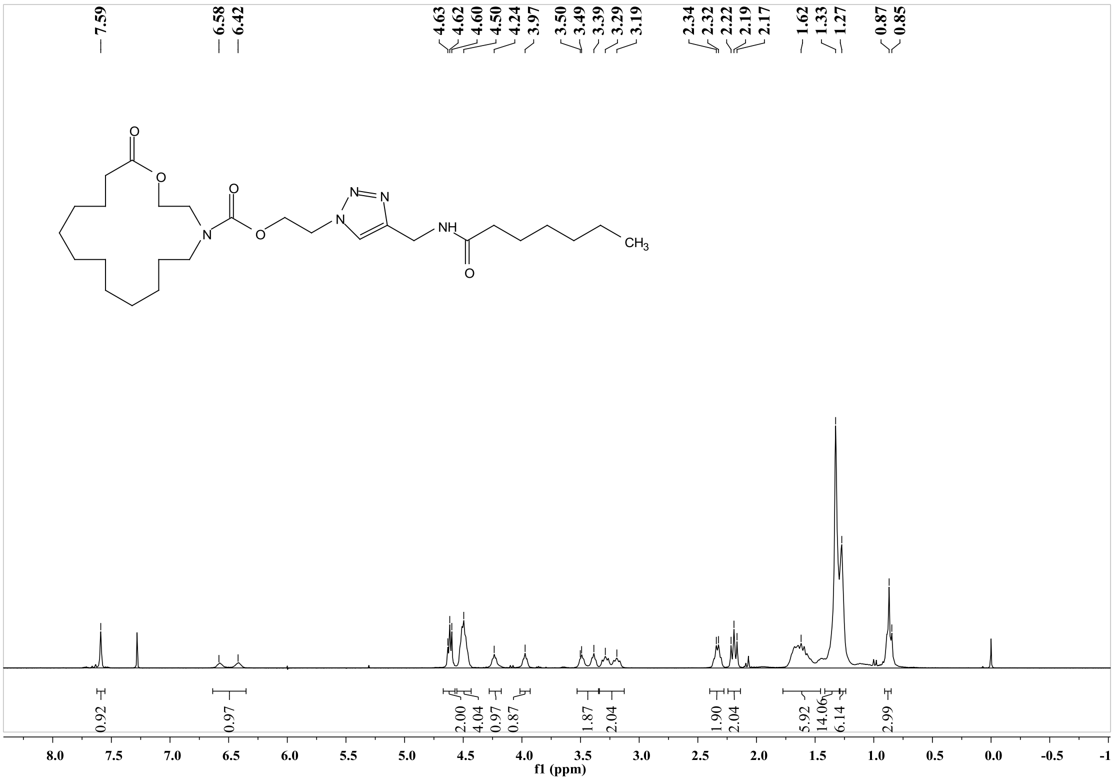
7.59
6.58
6.42
4.63
4.62
4.60
4.50
4.24
3.97
3.50
3.49
3.39
3.29
3.19
2.34
2.32
2.22
2.19
2.17
1.62
1.33
1.27
0.87
0.85
O
O
O
N
O
N
N=N
NH
O
CH3
0.92
0.97
2.00
4.04
0.97
0.87
1.87
2.04
1.90
2.04
5.92
14.06
6.14
2.99
8.0
7.5
7.0
6.5
6.0
5.5
5.0
4.5
4.0
3.5
3.0
2.5
2.0
1.5
1.0
0.5
0.0
-0.5
-1
f1 (ppm)

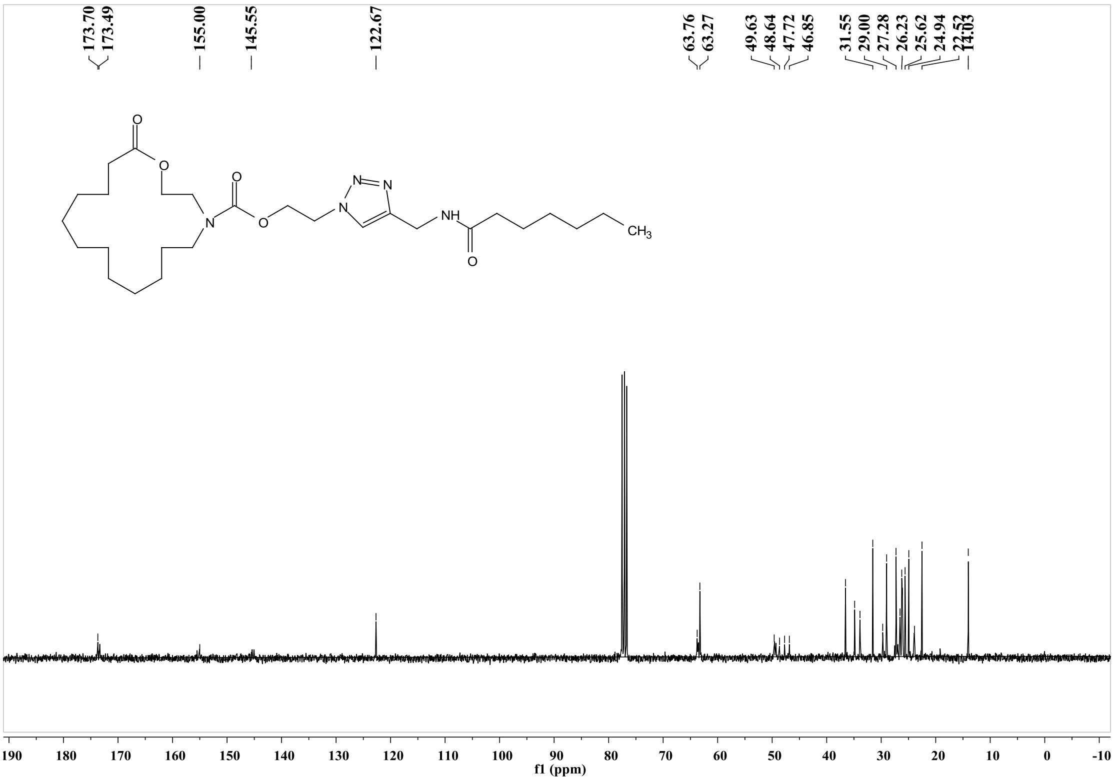
173.70
173.49
155.00
145.55
122.67
63.76
63.27
49.63
48.64
47.72
46.85
31.55
29.00
27.28
26.23
25.62
24.94
22.52
14.03
O
O
O
N
O
N
N=N
NH
O
CH3
190
180
170
160
150
140
130
120
110
100
90
80
70
60
50
40
30
20
10
0
-10
f1 (ppm)

Z16-5

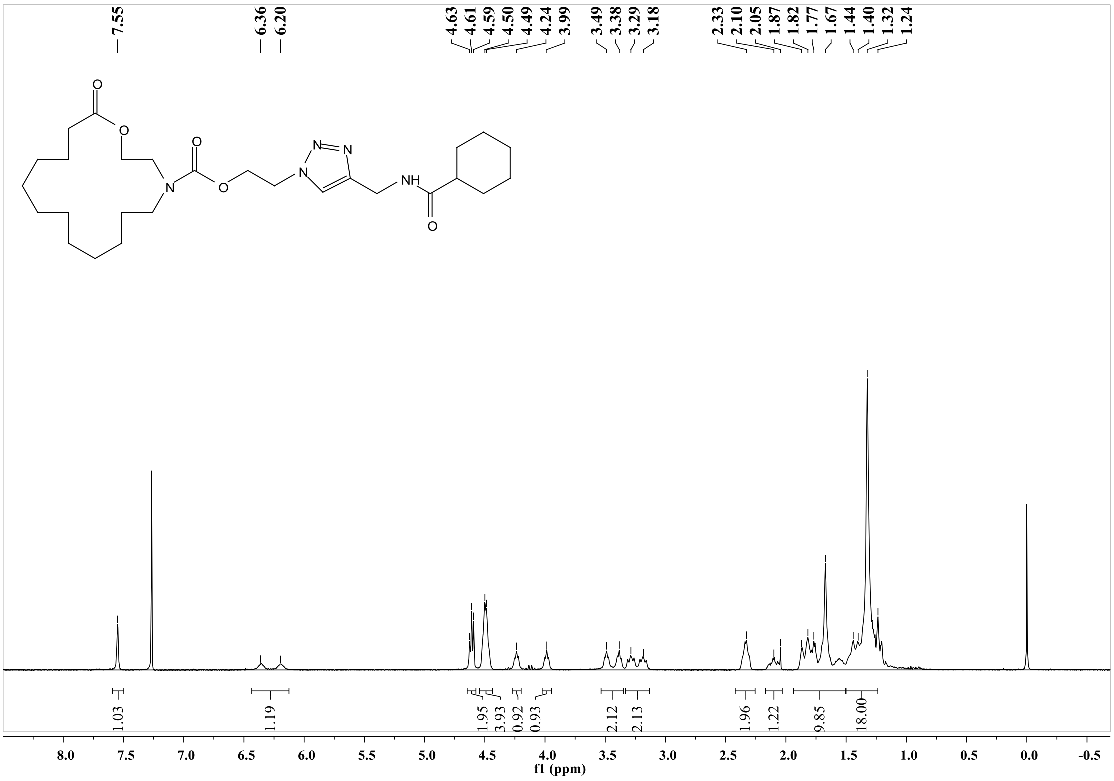
7.55
6.36
6.20
4.63
4.61
4.59
4.50
4.49
4.24
3.99
3.49
3.38
3.29
3.18
2.33
2.10
2.05
1.87
1.82
1.77
1.67
1.44
1.40
1.32
1.24
1.03
1.19
1.95
3.93
0.92
0.93
2.12
2.13
1.96
1.22
9.85
18.00
f1 (ppm)

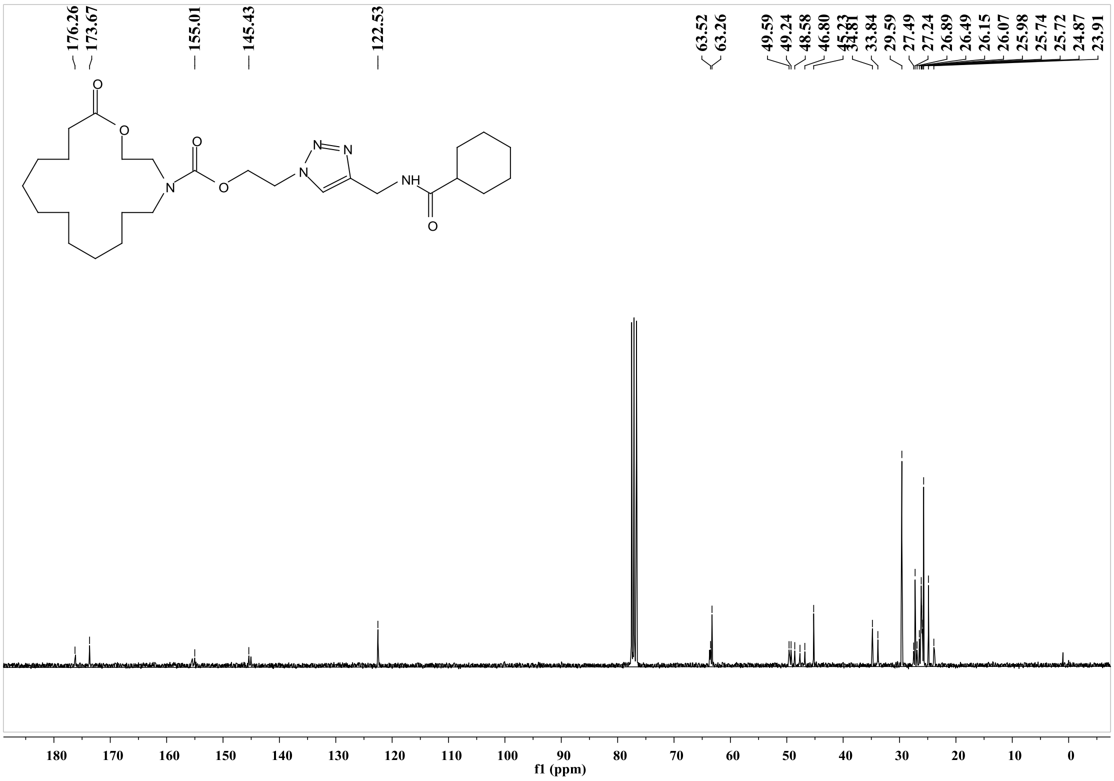
176.26
173.67
155.01
145.43
122.53
63.52
63.26
49.59
49.24
48.58
46.80
45.23
34.81
33.84
29.59
27.49
27.24
26.89
26.49
26.15
26.07
25.98
25.74
25.72
24.87
23.91
f1 (ppm)

Z16-6

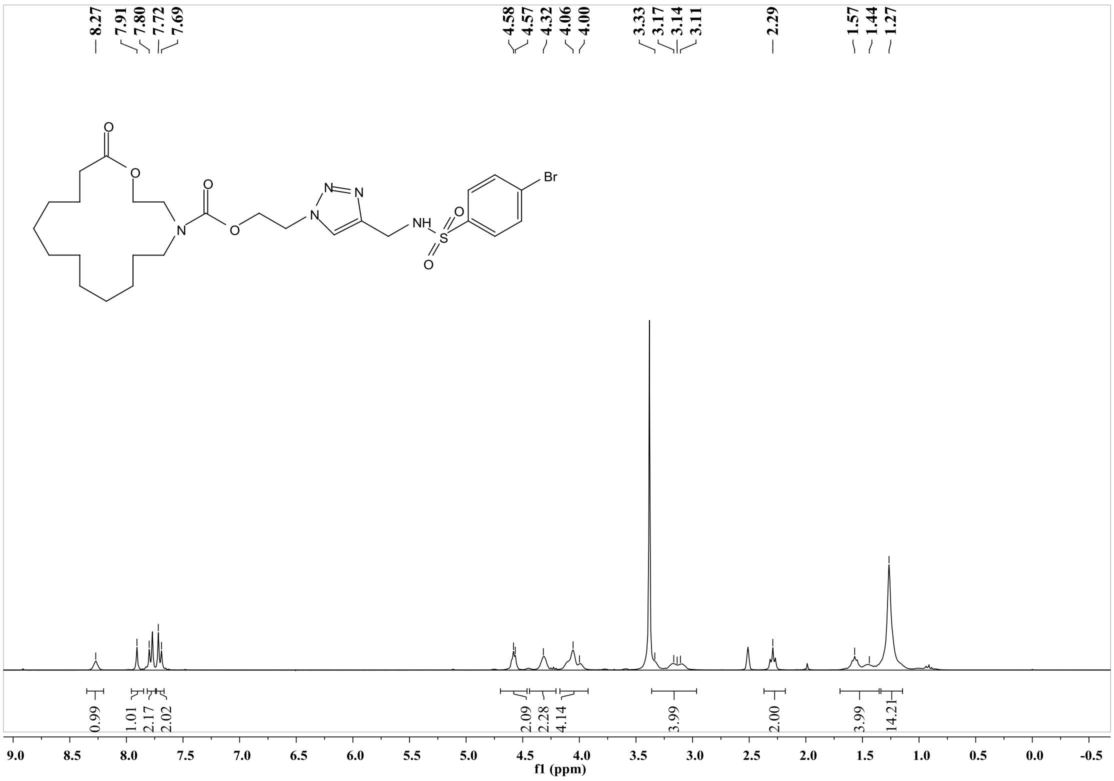
8.27
7.91
7.80
7.72
7.69
4.58
4.57
4.32
4.06
4.00
3.33
3.17
3.14
3.11
2.29
1.57
1.44
1.27
Br
0.99
1.01
2.17
2.02
2.09
2.28
4.14
3.99
2.00
3.99
14.21
f1 (ppm)

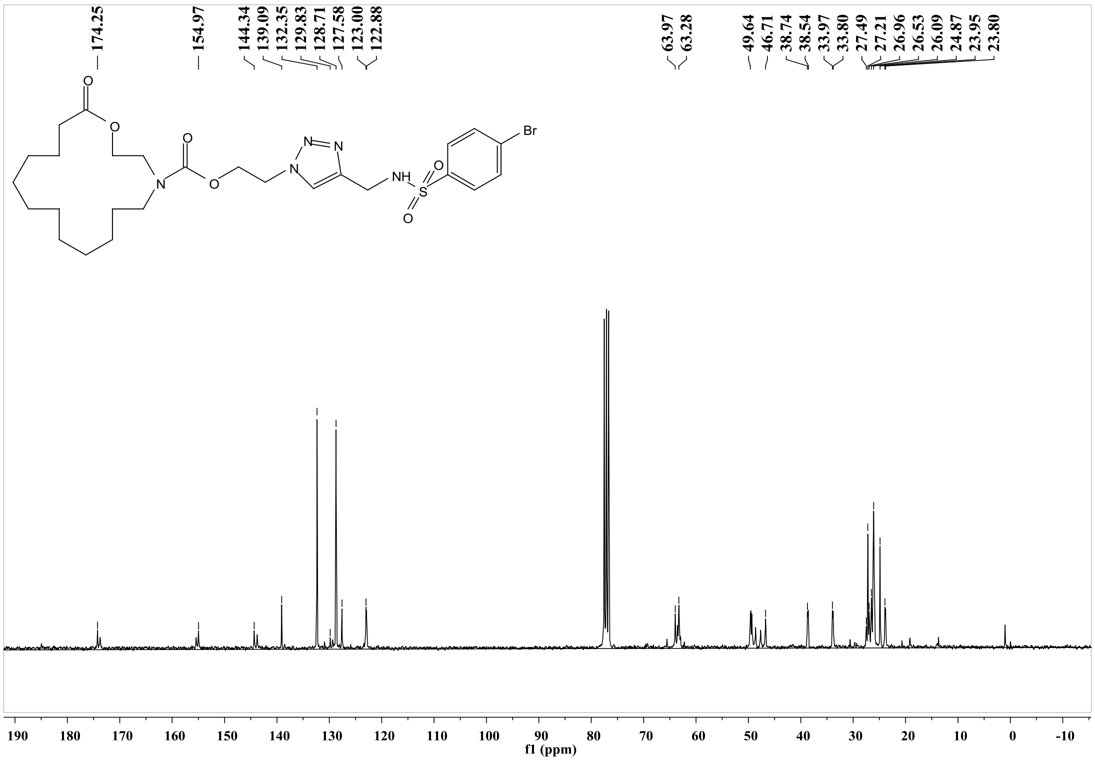
174.25
154.97
144.34
139.09
132.35
129.83
128.71
127.58
123.00
122.88
63.97
63.28
49.64
46.71
38.74
38.54
33.97
33.80
27.49
27.21
26.96
26.53
26.09
24.87
23.95
23.80
Br
f1 (ppm)

Z16-7

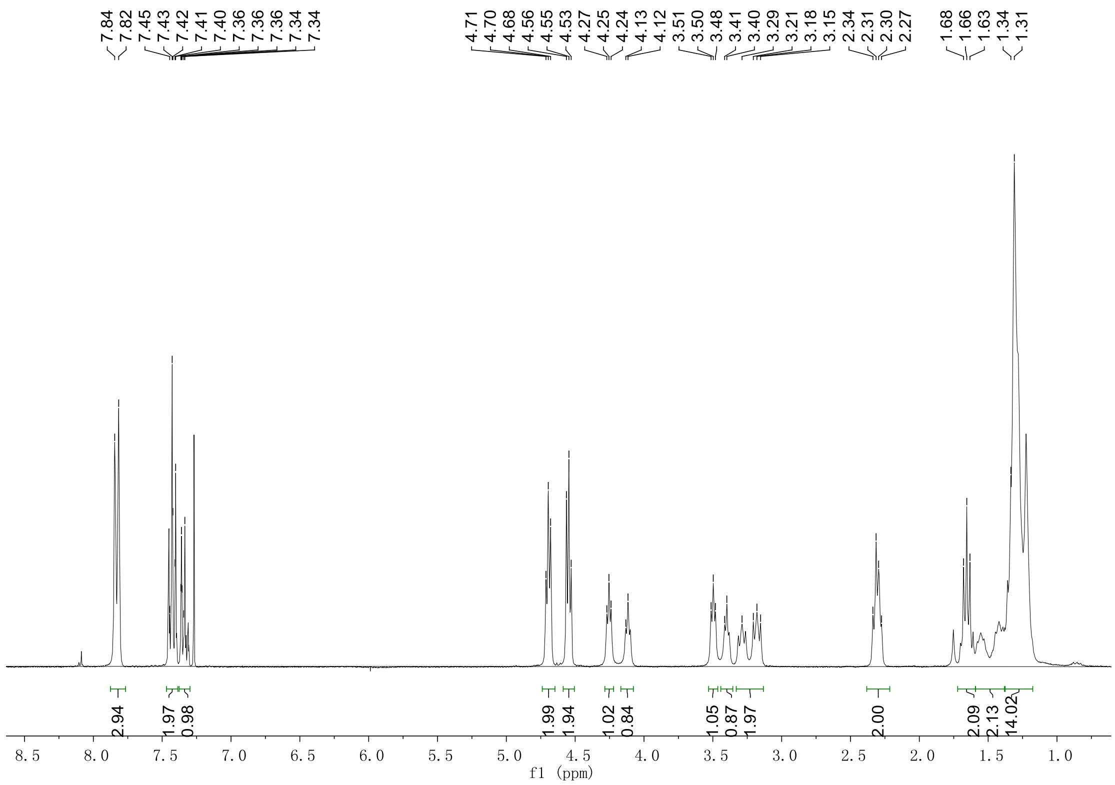
7.84
7.82
7.45
7.43
7.42
7.41
7.40
7.36
7.36
7.36
7.34
7.34
4.71
4.70
4.68
4.56
4.55
4.53
4.27
4.25
4.24
4.13
4.12
3.51
3.50
3.48
3.41
3.40
3.29
3.21
3.18
3.15
2.34
2.31
2.30
2.27
1.68
1.66
1.63
1.34
1.31
2.94
1.97
0.98
1.99
1.94
1.02
0.84
1.05
0.87
1.97
2.00
2.09
2.13
14.02
f1 (ppm)

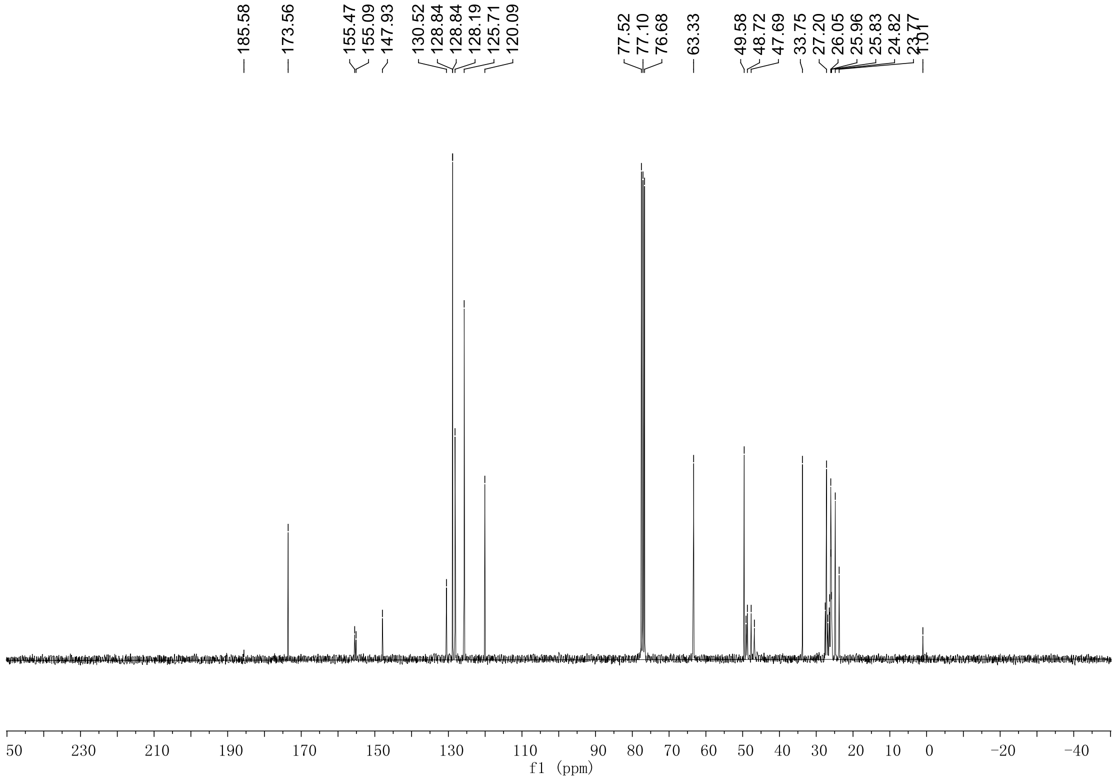
185.58
173.56
155.47
155.09
147.93
130.52
128.84
128.84
128.19
125.71
120.09
77.52
77.10
76.68
63.33
49.58
48.72
47.69
33.75
27.20
26.05
25.96
25.83
24.82
23.77
1.01
f1 (ppm)

Z16-8

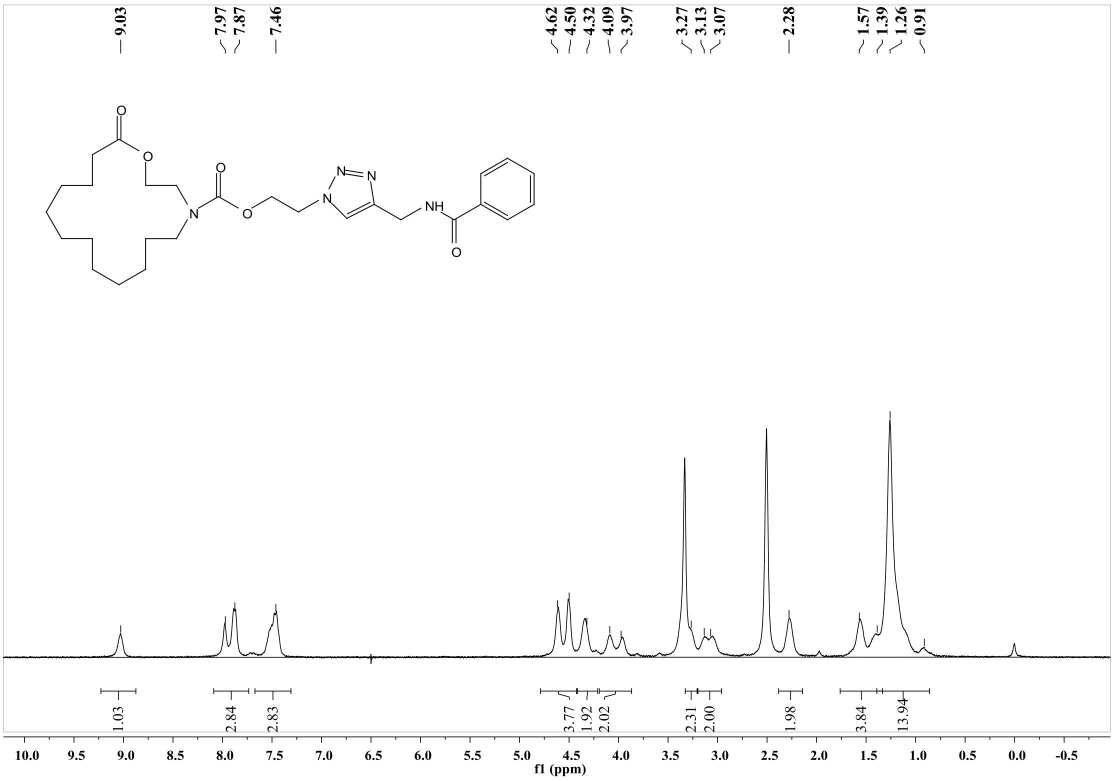

9.03
7.97
7.87
7.46
4.62
4.50
4.32
4.09
3.97
3.27
3.13
3.07
2.28
1.57
1.39
1.26
0.91
O
O
O
O
N
N
N
N
NH
O
1.03
2.84
2.83
3.77
1.92
2.02
2.31
2.00
1.98
3.84
13.94
10.0 9.5 9.0 8.5 8.0 7.5 7.0 6.5 6.0 5.5 5.0 4.5 4.0 3.5 3.0 2.5 2.0 1.5 1.0 0.5 0.0 -0.5
f1 (ppm)

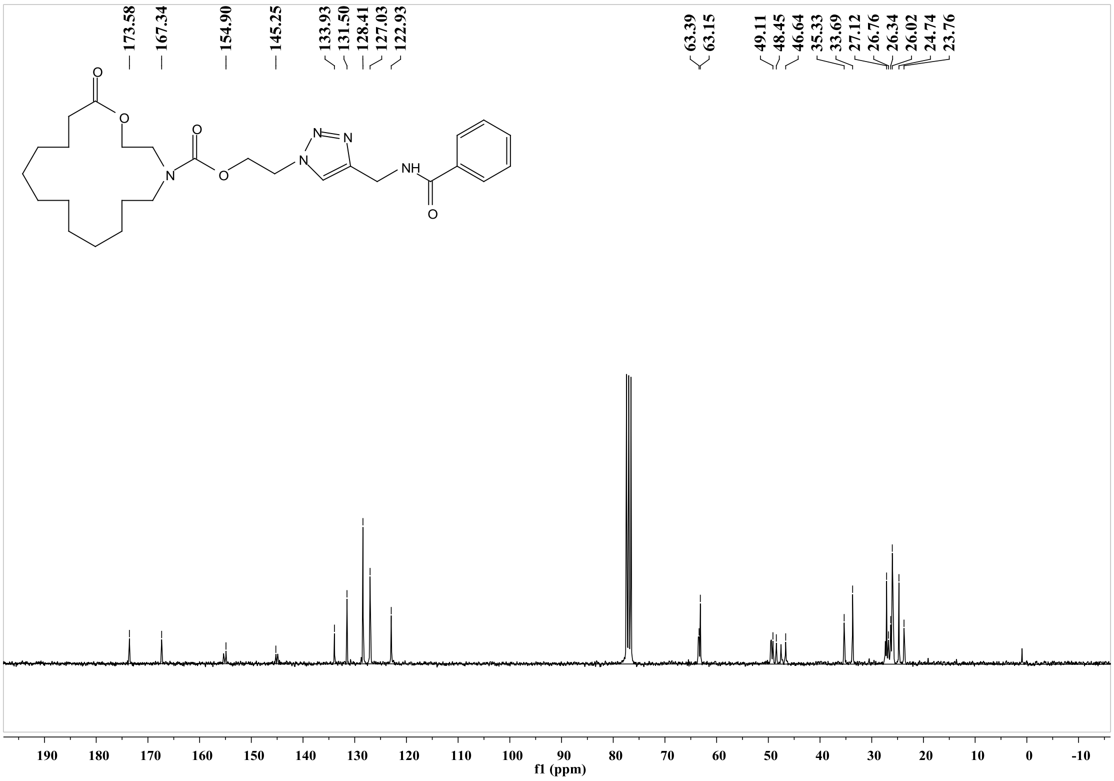

173.58
167.34
154.90
145.25
133.93
131.50
128.41
127.03
122.93
63.39
63.15
49.11
48.45
46.64
35.33
33.69
27.12
26.76
26.34
26.02
24.74
23.76
O
O
O
O
N
N
N
N
NH
O
190 180 170 160 150 140 130 120 110 100 90 80 70 60 50 40 30 20 10 0 -10
f1 (ppm)

Z16-9

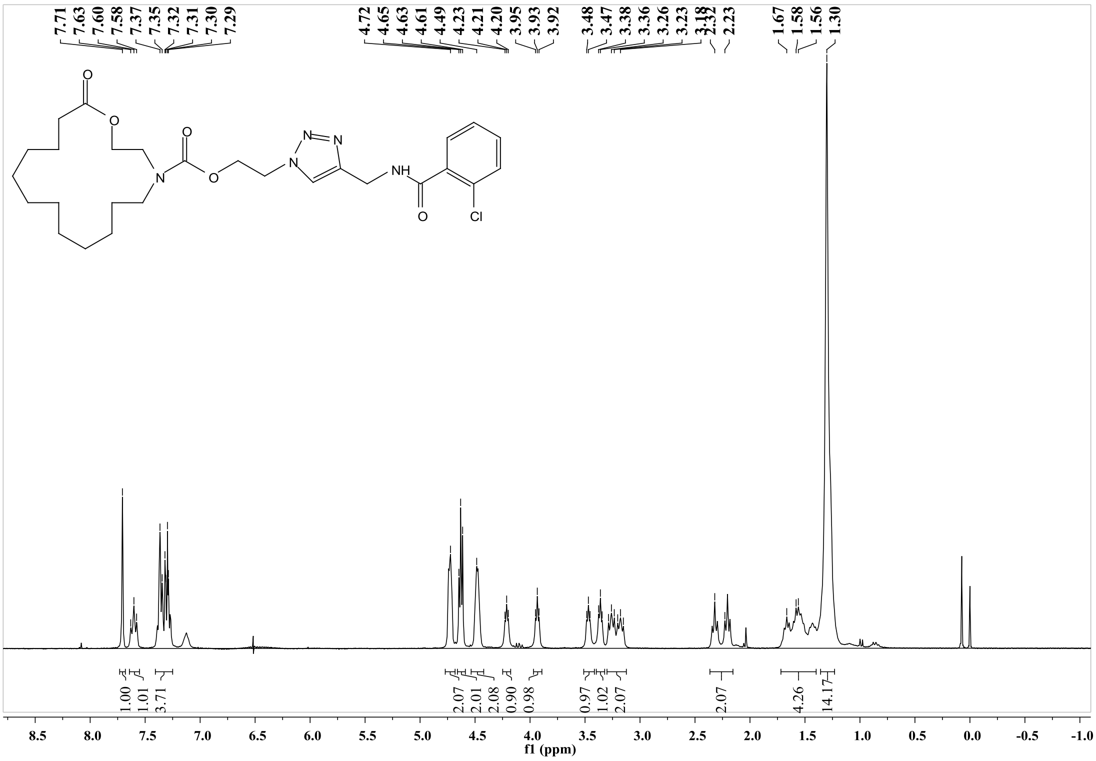
7.71
7.63
7.60
7.58
7.37
7.35
7.32
7.31
7.30
7.29
4.72
4.65
4.63
4.61
4.49
4.23
4.21
4.20
3.95
3.93
3.92
3.48
3.47
3.38
3.36
3.26
3.23
3.18
2.32
2.23
1.67
1.58
1.56
1.30
1.00
1.01
3.71
2.07
2.01
2.08
0.90
0.98
0.97
1.02
2.07
2.07
4.26
14.17
f1 (ppm)

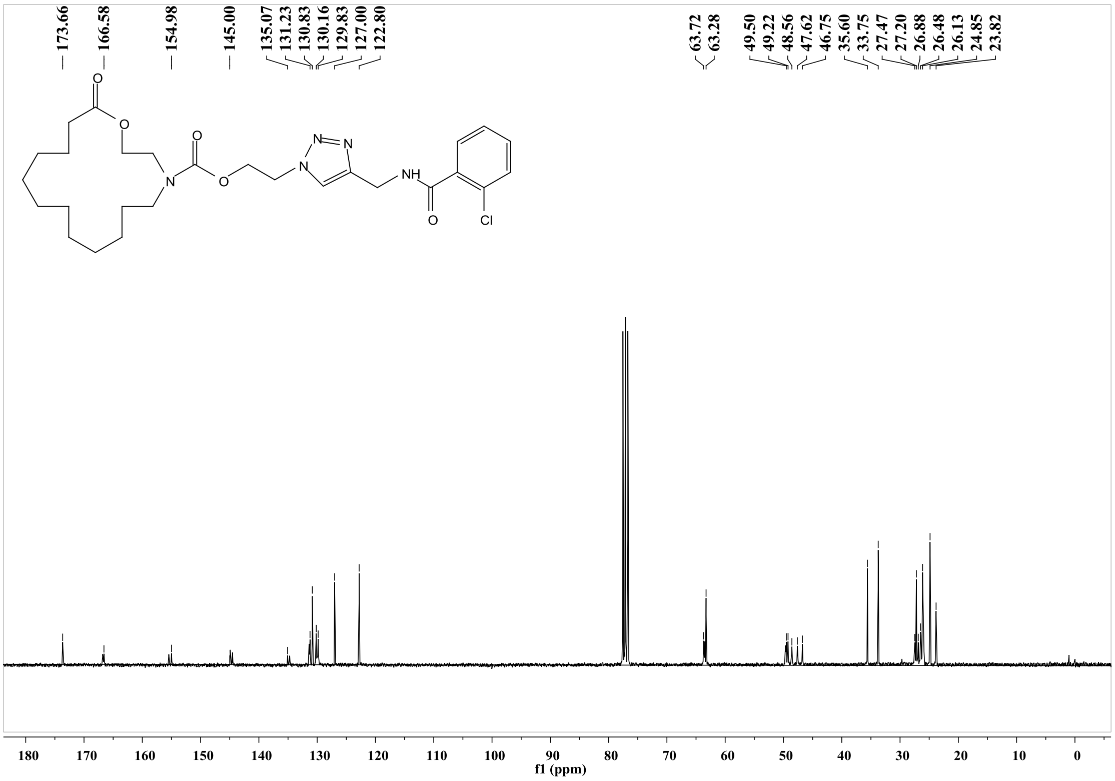
173.66
166.58
154.98
145.00
135.07
131.23
130.83
130.16
129.83
127.00
122.80
63.72
63.28
49.50
49.22
48.56
47.62
46.75
35.60
33.75
27.47
27.20
26.88
26.48
26.13
24.85
23.82
f1 (ppm)

Z16-10

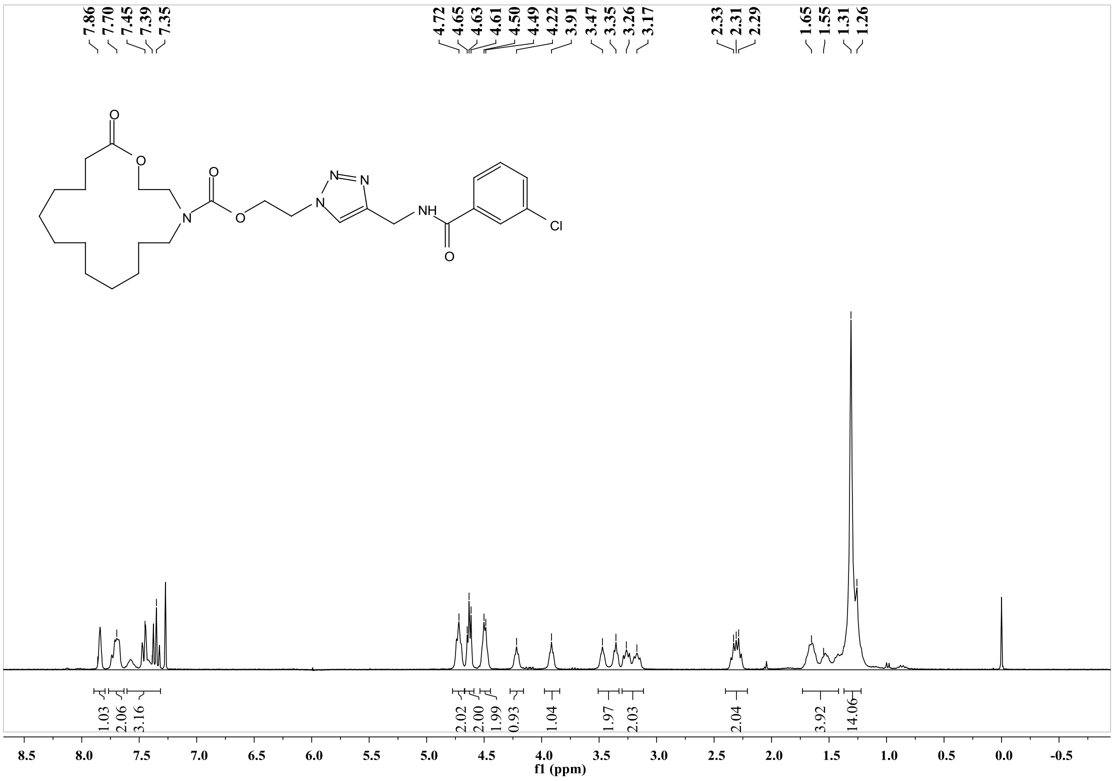
7.86
7.70
7.45
7.39
7.35
4.72
4.65
4.63
4.61
4.50
4.49
4.22
3.91
3.47
3.35
3.26
3.17
2.33
2.31
2.29
1.65
1.55
1.31
1.26
O
O
O
N
O
N
N
N
N
NH
O
Cl
1.03
2.06
3.16
2.02
2.00
1.99
0.93
1.04
1.97
2.03
2.04
3.92
14.06
f1 (ppm)

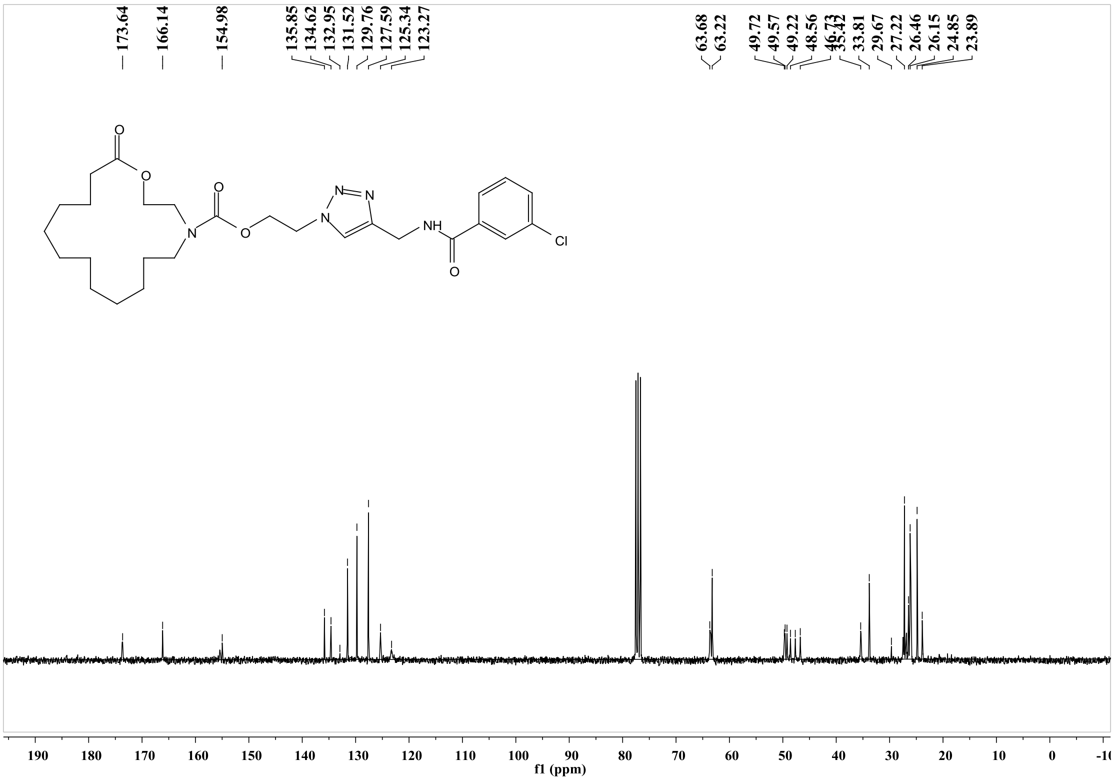
173.64
166.14
154.98
135.85
134.62
132.95
131.52
129.76
127.59
125.34
123.27
63.68
63.22
49.72
49.57
49.22
48.56
33.81
29.67
27.22
26.46
26.15
24.85
23.89
O
O
O
N
O
N
N
N
N
NH
O
Cl
f1 (ppm)

Z16-11

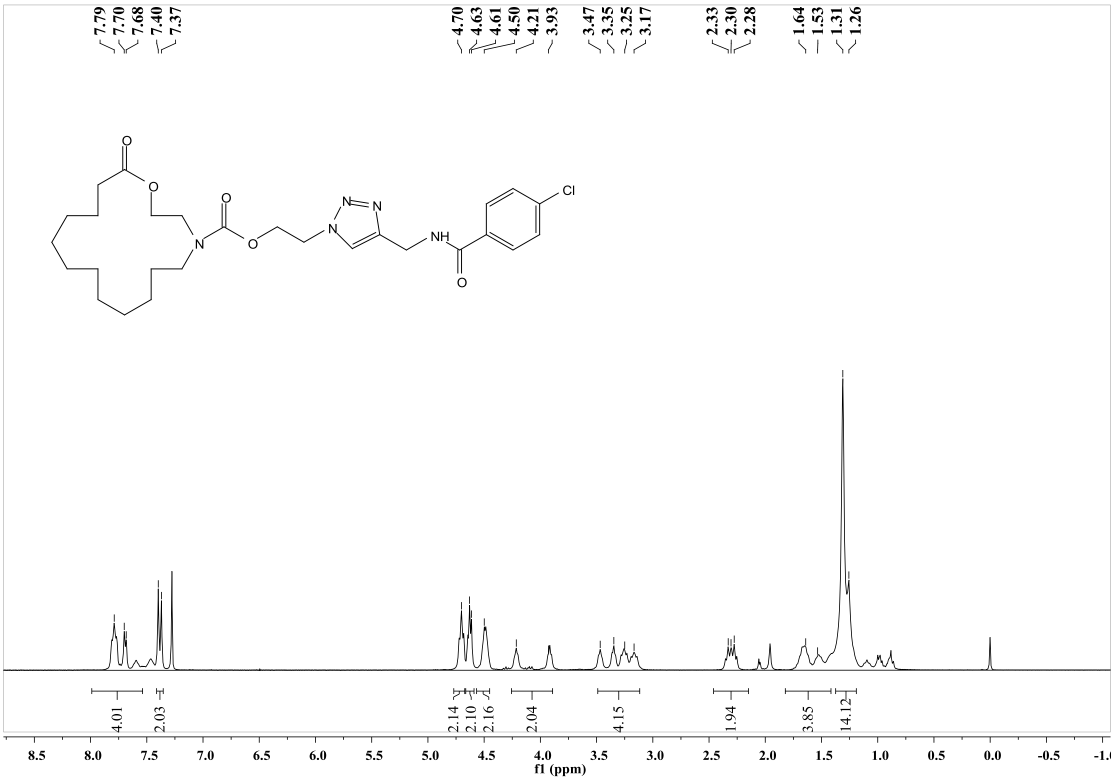
7.79
7.70
7.68
7.40
7.37
4.70
4.63
4.61
4.50
4.21
3.93
3.47
3.35
3.25
3.17
2.33
2.30
2.28
1.64
1.53
1.31
1.26
O
O
N
O
O
N
N
N
NH
O
Cl
4.01
2.03
2.14
2.10
2.16
2.04
4.15
1.94
3.85
14.12
8.5
8.0
7.5
7.0
6.5
6.0
5.5
5.0
4.5
4.0
3.5
3.0
2.5
2.0
1.5
1.0
0.5
0.0
-0.5
f1 (ppm)

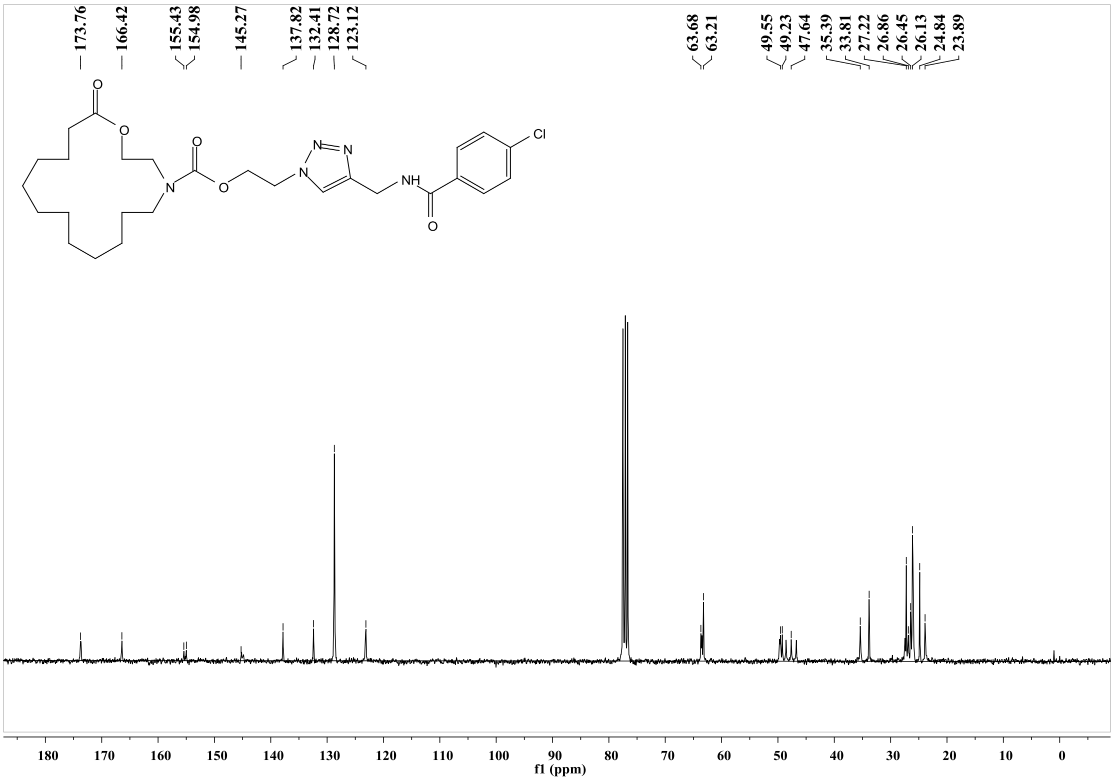
173.76
166.42
155.43
154.98
145.27
137.82
132.41
128.72
123.12
63.68
63.21
49.55
49.23
47.64
35.39
33.81
27.22
26.86
26.45
26.13
24.84
23.89
O
O
N
O
O
N
N
N
NH
O
Cl
180
170
160
150
140
130
120
110
100
90
80
70
60
50
40
30
20
10
0
f1 (ppm)

Z16-12

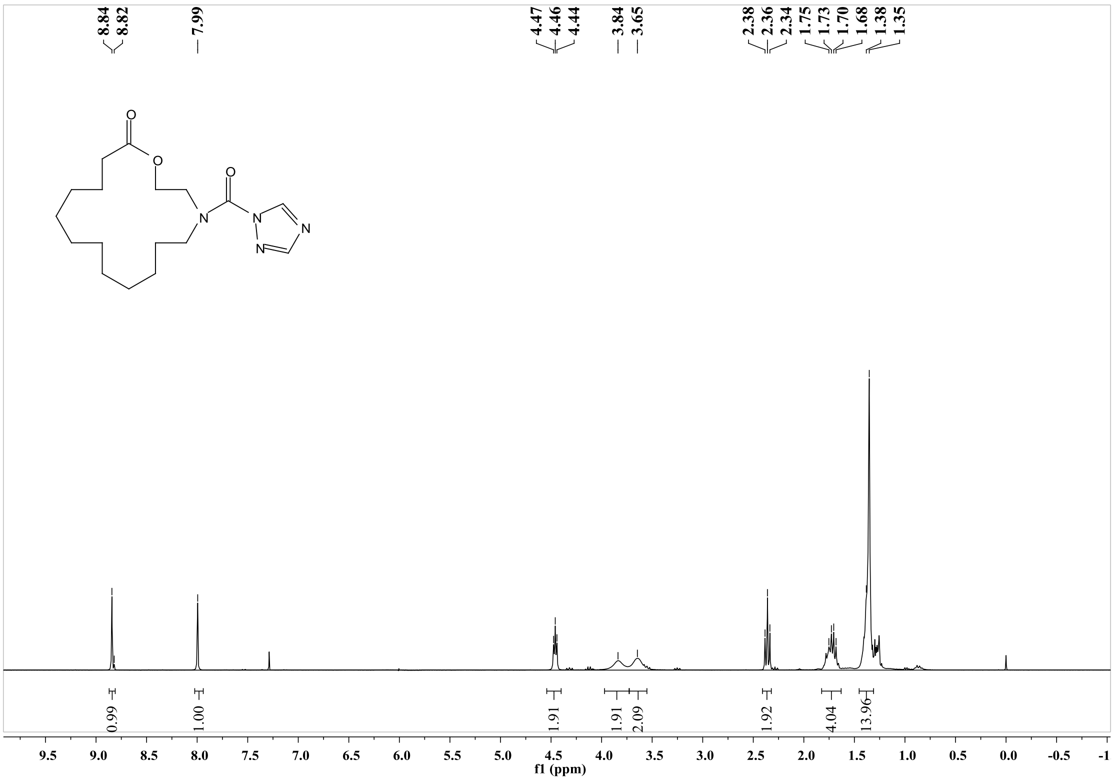

8.84
8.82
7.99
4.47
4.46
4.44
3.84
3.65
2.38
2.36
2.34
1.75
1.73
1.70
1.68
1.38
1.35
0.99
1.00
1.91
1.91
2.09
1.92
4.04
13.96
f1 (ppm)

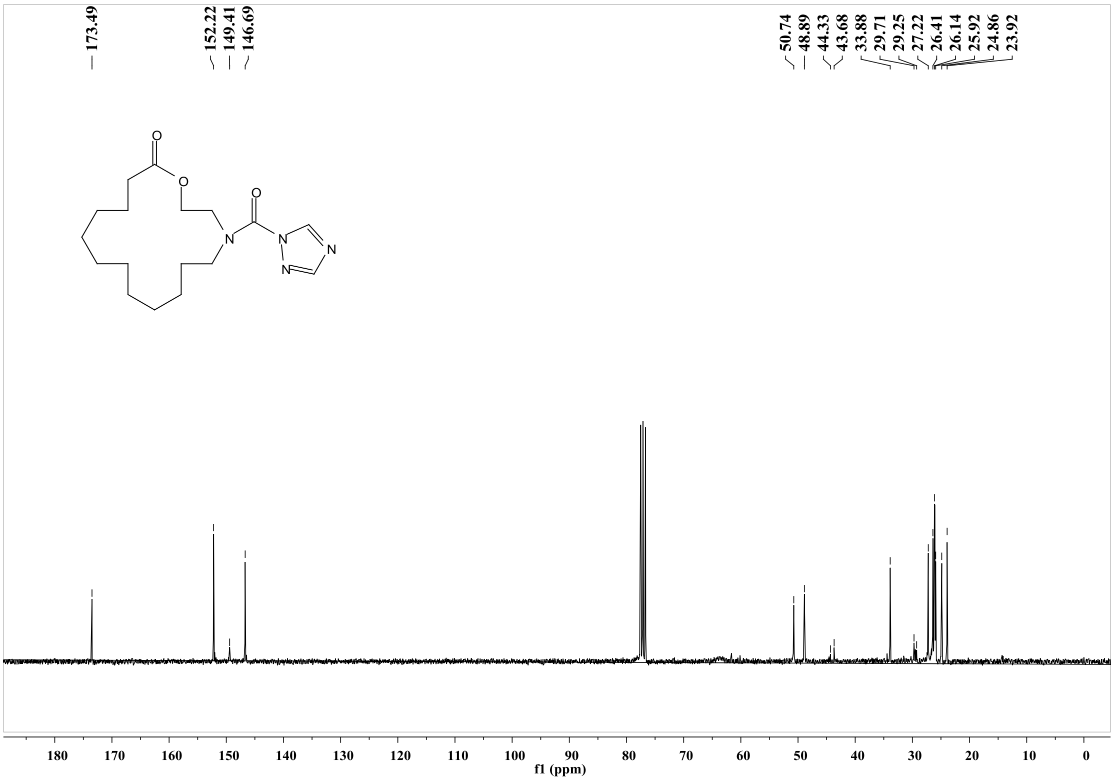

173.49
152.22
149.41
146.69
50.74
48.89
44.33
43.68
33.88
29.71
29.25
27.22
26.41
26.14
25.92
24.86
23.92
f1 (ppm)

Z16-13

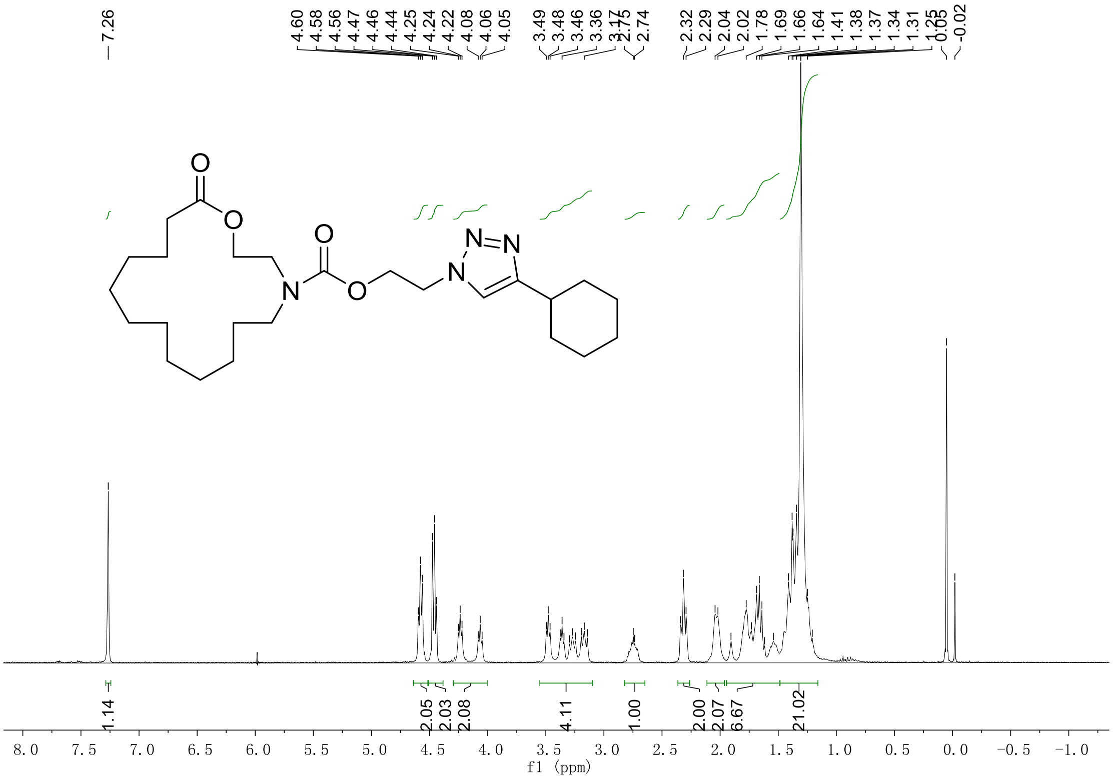
7.26
4.60 4.58 4.56 4.47 4.46 4.44 4.25 4.24 4.22 4.08 4.06 4.05
3.49 3.48 3.46 3.36 2.74
2.32 2.29 2.04 2.02 1.78 1.69 1.66 1.64 1.41 1.38 1.37 1.34 1.31 -0.02
O
O
O
O
N
N
N=N
1.14
2.05
2.03
2.08
4.11
1.00
2.00
2.07
6.67
21.02
8.0 7.5 7.0 6.5 6.0 5.5 5.0 4.5 4.0 3.5 3.0 2.5 2.0 1.5 1.0 0.5 0.0 -0.5 -1.0
f1 (ppm)

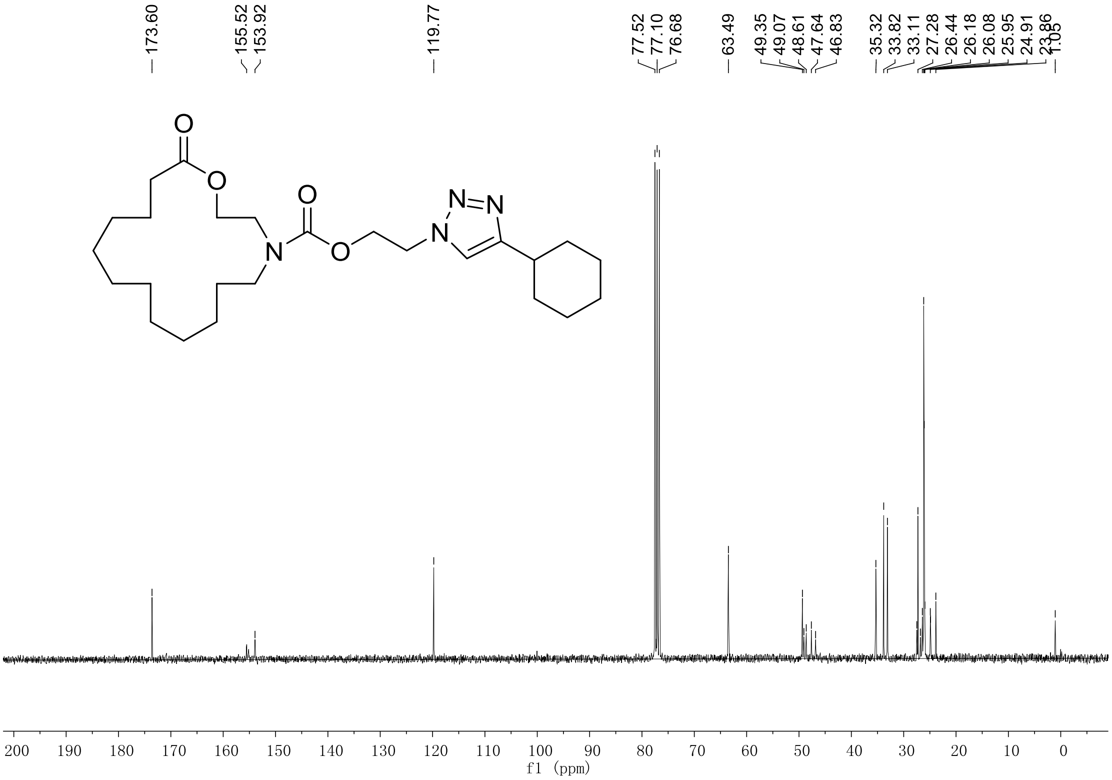
173.60
155.52 153.92
119.77
77.52 77.10 76.68
63.49
49.35 49.07 48.61 47.64 46.83
35.32 33.82 33.11 27.28 26.44 26.18 26.08 25.95 24.91
O
O
O
O
N
N
N=N
200 190 180 170 160 150 140 130 120 110 100 90 80 70 60 50 40 30 20 10 0
f1 (ppm)

Z16-14

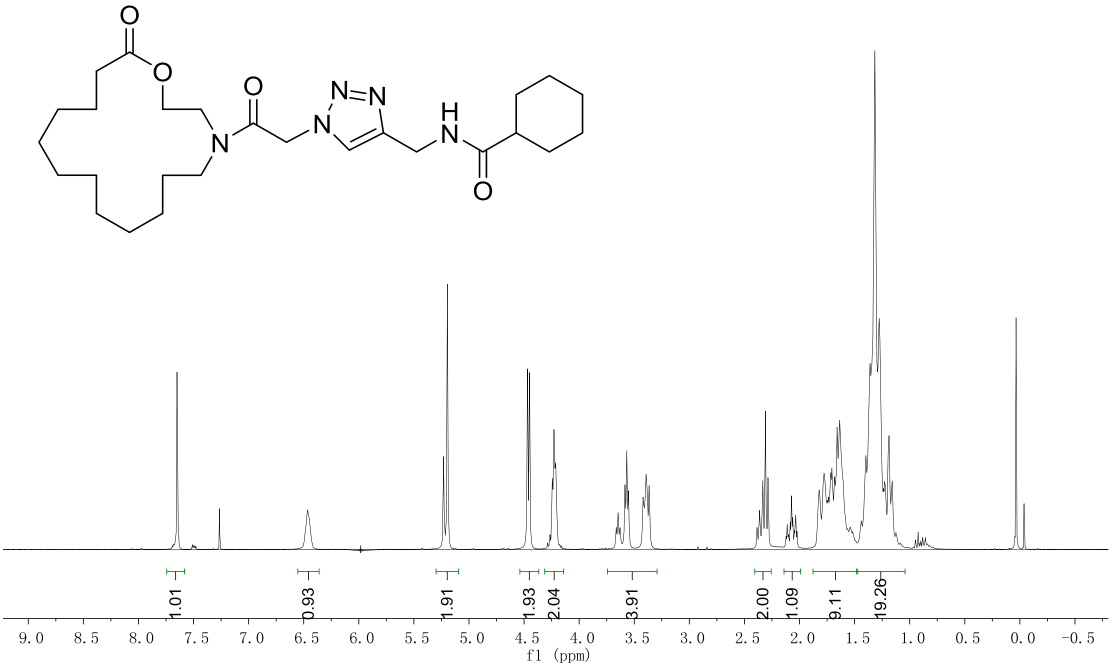

O
O
O
N
N
N=N
H
N
O
1.01
0.93
1.91
1.93
2.04
3.91
2.00
1.09
9.11
19.26
9.0 8.5 8.0 7.5 7.0 6.5 6.0 5.5 5.0 4.5 4.0 3.5 3.0 2.5 2.0 1.5 1.0 0.5 0.0 -0.5
f1 (ppm)

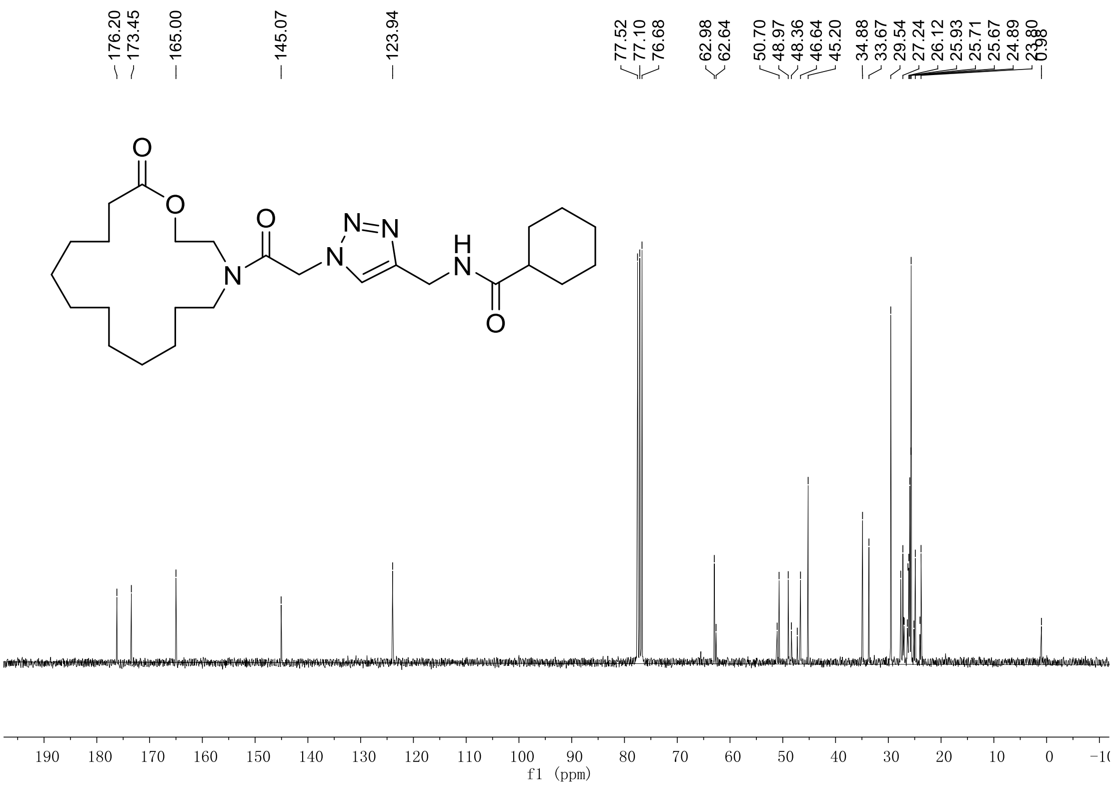

176.20
173.45
165.00
145.07
123.94
77.52
77.10
76.68
62.98
62.64
50.70
48.97
48.36
46.64
45.20
34.88
33.67
29.54
27.24
26.12
25.93
25.71
25.67
24.89
23.80
0.98
190 180 170 160 150 140 130 120 110 100 90 80 70 60 50 40 30 20 10 0
f1 (ppm)
